# TRAIL splice variant TRAILshort disrupts T cell receptor signaling and promotes immune tolerance in vivo

**DOI:** 10.1172/JCI194449

**Published:** 2026-08-03

**Authors:** Shahrzad Jalali, Sekar Natesampillai, Zilin Nie, Ying Zhang, Ismail Can, Aswath P. Chandrasekar, Brianna M. Hameister, Cristina Correia, Tuantuan V. Zhao, Dong-Gi Mun, Enrique Garcia-Rivera, Robert Matson, Ashton Krogman, Mark A. Maynes, Robin Batchelor, Dileep D. Monie, Hu Li, Atta Behfar, Saad S. Kenderian, Akhilesh Pandey, Stephen M. Ansell, Timucin Taner, Cornelia Weyand, Daniel D. Billadeau, Andrew D. Badley

**Affiliations:** 1 Division of Infectious Diseases,; 2 Division of Hematology,; 3 Department of Laboratory Medicine and Pathology, and; 4 Molecular Pharmacology and Experimental Therapeutics, Mayo Clinic, Rochester, Minnesota, USA.; 5 Department of Medicine, Mayo Clinic College of Medicine and Science, Rochester, Minnesota, USA.; 6 Formation Bio, New York, New York, USA.; 7 nference Inc., Cambridge, Massachusetts, USA.; 8 Department of Neurological Surgery, Albert Einstein College of Medicine-Montefiore Medical Center, Bronx, New York, USA.; 9 Department of Cardiovascular Medicine,; 10 Department of Molecular Medicine, and; 11 Center for Individualized Medicine, Mayo Clinic, Rochester, Minnesota, USA.; 12 Manipal Academy of Higher Education, Manipal, Karnataka, India.; 13 Department of Immunology, and; 14 Department of Surgery, Mayo Clinic, Rochester, Minnesota, USA.

**Keywords:** AIDS/HIV, Immunology, Oncology, Cellular immune response

## Abstract

TRAIL is a TNF family ligand that trimerizes TRAIL-R1 (DR4) or TRAIL-R2 (DR5) to induce apoptosis, necroptosis, and/or NF-κB activation in receptor-bearing cells. We previously identified TRAILshort as a splice variant of TRAIL that lacks cysteine 230, cannot trimerize, and acts as a dominant-negative ligand that blocks TRAIL-mediated apoptosis. TRAILshort is expressed on cell surfaces and within extracellular vesicles, enabling it to confer TRAIL resistance to both producing and bystander cells. In this study, we showed that elevated TRAILshort levels were associated with chronic viral infections, cancer, and autoimmune diseases, suggesting a link to impaired immune regulation. Using unbiased phosphoproteomics and mechanistic studies, we demonstrated that TRAILshort binding to DR5 recruited and activated the phosphatase Src homology region 2 domain–containing phosphatase 1 (SHP-1), leading to zeta-chain-associated protein kinase 70 (ZAP-70) dephosphorylation, disruption of ZAP-70–CD3ζ interactions, and impaired T cell receptor signaling, thereby reducing T cell activation, proliferation, and cytokine production in response to antigen or CD3/CD28 ligation. Genetic or pharmacologic SHP-1 inhibition reverses these effects. In humanized mouse models, TRAILshort promoted the persistence of transformed mouse embryonic fibroblasts (MEFs) and L428 and antagonized CD19-directed CAR T cell activity, revealing TRAILshort as an immunomodulator of T cell function with therapeutic implications, including blocking TRAILshort to restore T cell immunity or delivering TRAILshort to enforce tolerance.

## Abstract 2

TRAILshort, a variant of TRAIL, suppresses T cell function and immune responses in cancer and infectious diseases, highlighting its potential as a therapeutic target.

## Graphical abstract

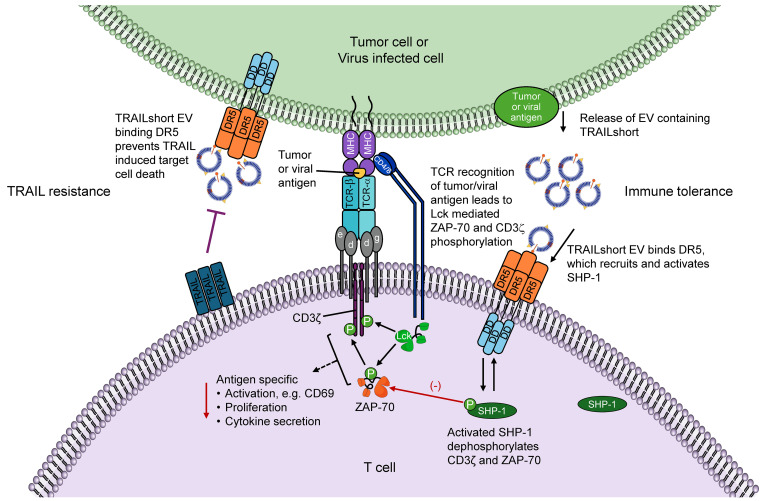

## Introduction

Virus-infected cells and cancer cells have evolved overlapping mechanisms to evade immune surveillance. HIV, hepatitis C virus, and CMV upregulate programmed death–ligand 1 (PD-L1) to suppress T cell activation through the programmed cell death protein 1/PD-L1 (PD-1/PD-L1) axis, driving T cell exhaustion and enabling chronic infection (1–3). Cancers including melanoma, lung cancer, and breast cancer exploit the same pathway (4–6). Both settings also feature secretion of immunosuppressive factors such as TGF-β and IL-10, which suppress effector T cell function and expand Tregs (7–10). Understanding these shared mechanisms has illuminated the pathophysiology of cancer and chronic infections and has guided the development of targeted strategies to overcome immune escape.

TRAIL is a death-inducing TNF superfamily ligand expressed primarily on activated T and NK cells, which use it to kill virally infected and transformed cells while sparing healthy tissue (11). TRAIL binds 5 receptors (TRAIL-R1 through R4 and soluble osteoprotegerin); TRAIL-R1 and TRAIL-R2, also known as DR4 and DR5, transmit apoptotic signals (12), while R3 and R4 do not (13). Beyond apoptosis, TRAIL can also induce necroptosis (14), activate NF-κB signaling (15), and modulate TCR signaling (16). Although TRAIL resulted in tumor regression in animal models (17, 18), clinical trials of TRAIL agonists failed to show significant anticancer activity (19, 20), underscoring the complexity of the TRAIL:TRAIL/receptor axis and shifting the focus toward understanding the determinants of TRAIL sensitivity and resistance (21–23).

The TRAIL/TRAIL receptor axis also exerts protumorigenic effects under specific conditions. In TRAIL-resistant tumor cells, receptor engagement activates nonapoptotic NF-κB and MAPK/p38 signaling, promoting tumor cell survival, invasion, metastasis, and a proinflammatory microenvironment (20, 24), potentially explaining the limited clinical success of TRAIL agonists. TRAIL signaling also modulates T cell activation: TRAIL receptor–deficient mice exhibit heightened T cell responses, and chronic TRAIL blockade provokes autoimmunity, suggesting that sustained receptor engagement dampens adaptive immunity and, in the cancer setting, may further favor immune evasion (25). These observations raise the possibility that endogenous modulators of TRAIL signaling could serve as previously unrecognized mediators of immune escape.

We previously identified TRAILshort, a splice variant of TRAIL in which a splicing event excises exons 3 and 4, introducing a frameshift in exon 5 and generating a novel 11–amino acid C-terminus (22). Full-length TRAIL initiates apoptosis signaling through trimerization, whereby 3 cysteine 230 residues coordinate a zinc ion to stabilize the trimer; receptor binding then drives TRAIL receptor trimerization, which is essential for effective apoptosis signaling (21). The splicing event that generates TRAILshort eliminates cysteine 230, preventing trimerization and abolishing apoptosis signaling. Nevertheless, TRAILshort retains the ability to bind death-inducing TRAIL receptors, with preferential binding to DR5, and functions as a dominant-negative ligand that competitively blocks full-length TRAIL binding, thereby conferring resistance to TRAIL-mediated apoptosis (26).

TRAILshort expression is induced by type I IFNs and TLR-7, -8, and -9 agonists (26), receptors activated by both pathogen-associated molecular patterns (PAMPs) and damage-associated molecular patterns (DAMPs), implicating TRAILshort in immune contexts beyond infection alone. Consistent with the known roles of type I IFNs and TLR signaling in cancer, we found that diverse primary human malignancies, including HPV-associated tumors and nonviral cancers such as ovarian and pancreatic cancer, express elevated TRAILshort levels (27).

Building on these observations, this study identifies TRAILshort as a mediator of T cell hypofunction by impairing proximal TCR signaling in response to CD3/CD28 ligation or cognate antigen exposure, suppressing T cell activation, cytokine secretion, and proliferation in a Src homology region 2 domain–containing phosphatase 1–dependent (SHP-1–dependent) manner. In humanized mouse models, TRAILshort was sufficient to induce tolerance to xenografts, antagonized the antigen-specific cytotoxicity of CAR-T cells against human tumors, and imparted a growth advantage to tumors implanted into CD34 humanized mice.

These findings provide a mechanistic framework for how TRAILshort, expressed within the microenvironment of virus-infected cells and human tumors, antagonizes antigen-specific T cell recognition and effector function. Blocking TRAILshort may therefore represent a strategy to restore T cell–mediated immunity in malignancies and chronic infections, while delivery of TRAILshort may represent a strategy to induce tolerance.

## Results

### Increased TRAILshort expression is linked to infectious viral diseases and cancer.

We previously identified TRAILshort as a dominant-negative TRAIL receptor ligand that confers resistance to full-length TRAIL-induced apoptosis, which is detected in HIV-infected cell cultures and plasma from HIV^+^ individuals (22). Genetic inhibition or antibody blockade of TRAILshort increases death of HIV-infected cells in vitro, reducing HIV p24^+^ cells and viral loads (28). Subsequently, we showed that in malignant B cells, TRAILshort expression is similarly detected, and anti-TRAILshort antibodies enhance T cell–mediated killing of autologous B cell tumors (27).

**Figure 1:**
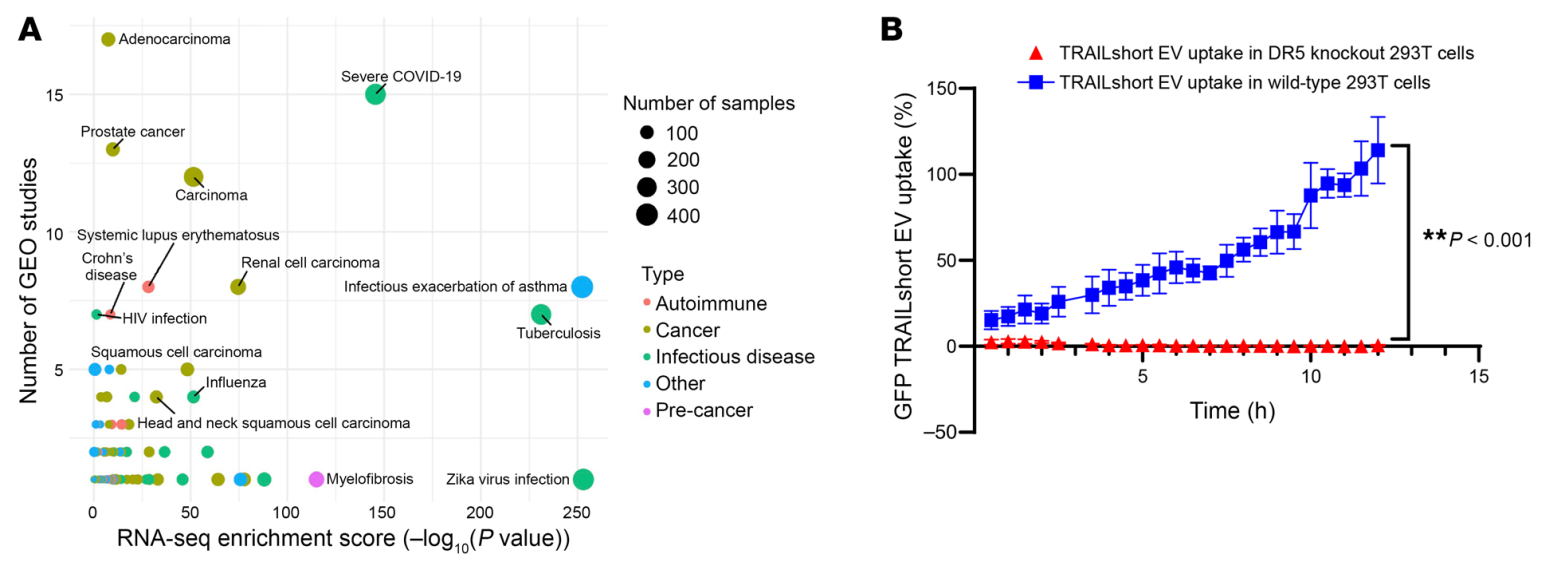
TRAILshort expression and DR5-dependent uptake of TRAILshort-containing EVs. (**A**) Analysis of human RNA-seq datasets obtained from the GEO database showing disease states associated with high TRAILshort expression. RNA-seq enrichment scores were determined by hypergeometric test with Benjamini-Hochberg FDR correction across high (top 5%) versus low (bottom 5%) TRAILshort-expressing samples. (**B**) DR5-dependent internalization of TRAILshort EVs. GFP-TRAILshort EVs were first labeled with an anti-TRAILshort antibody conjugated to a pH-sensitive dye and then incubated with wild-type or DR5-knockout 293T cells to monitor EV internalization. Representative line graph showing the kinetics of EV uptake by wild-type (blue) and DR5-knockout (red) 293T cells. **P < 0.001, by 2-way ANOVA. Data are presented as mean ± SEM.

To define the broader disease context of TRAILshort expression, we performed unbiased enrichment analysis of RNA-seq data from the Gene Expression Omnibus (GEO), revealing elevated TRAILshort RNA in viral infections (COVID-19, Zika), various human neoplasms, chronic infections (tuberculosis), premalignant conditions (myelofibrosis), and autoimmune diseases (Crohn’s disease, systemic lupus erythematosus) (Figure 1A and Supplemental Table 1; supplemental material available online with this article; https://doi.org/10.1172/JCI194449DS1). Although HIV showed modest enrichment in this global screen, prior work from our group has confirmed TRAILshort expression in HIV-infected cells and plasma of HIV^+^ individuals (22, 28), validating HIV as one of a broad spectrum of TRAILshort-high, disease states. Given that TRAILshort is elevated across such diverse disease states, all of which are characterized by varying degrees of immune dysfunction, we hypothesized that TRAILshort may directly modulate immune function.

### Cellular effects of TRAILshort-containing extracellular vesicles are mediated by the DR5 receptor.

We have previously shown that TRAILshort preferentially binds DR5 and that TRAILshort-bearing extracellular vesicles (EVs) convert TRAIL-sensitive cells to a resistant phenotype (26). To determine whether EV-associated TRAILshort can engage DR5 and initiate intracellular signaling, we generated GFP-tagged TRAILshort EVs from TRAIL-KO 293T cells (Supplemental Figure 1, A and B) and monitored their uptake by WT or DR5-KO 293T cells over 12 hours (Figure 1B and Supplemental Figure 1D). TRAILshort EVs were internalized by WT but not DR5-KO cells (*P* < 0.0001), indicating that EV binding and uptake was DR5 dependent and suggesting that TRAILshort-DR5 engagement may initiate downstream signaling events.

### TRAILshort-induced changes in phosphoproteomics pathways.

**Figure 2:**
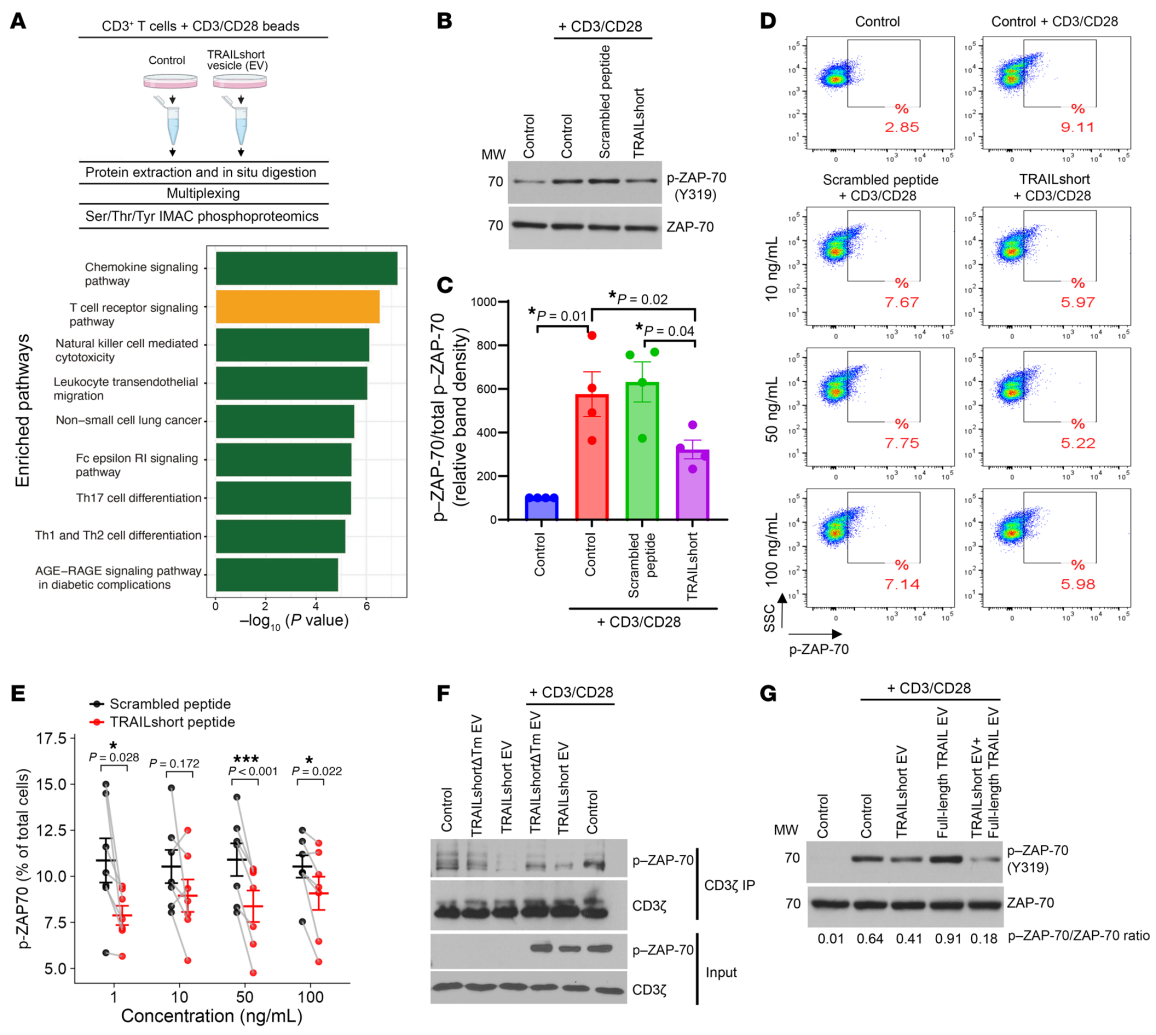
TRAILshort induces changes in the TCR phosphoproteome and inhibits proximal events in the TCR signaling pathway. (**A**) Schematic of the experimental design: CD3^+^ T cells were treated with TRAILshort EVs, stimulated with CD3/CD28 beads, and analyzed by phosphoproteomics. Bar graph shows differentially expressed phosphoproteins in TRAILshort EV–treated T cells versus untreated T cells. KEGG pathway enrichment analysis of phosphoproteins with a log_2_FC of greater than 1.5 or less than –0.9, performed using a hypergeometric test with Benjamini-Hochberg correction (adjusted *P* < 0.05), identified TCR signaling pathways as preferentially affected. (**B**) Western blot analysis of p–ZAP-70 following TRAILshort treatment. (**C**) Quantification showing reduced p–ZAP-70 levels in TRAILshort peptide–treated cells versus scrambled peptide controls (*n* = 4; *P < 0.05, by 2-tailed, paired *t* test with Holm correction for multiple comparisons, except control vs. +CD3/CD28, which was analyzed using a 1-sample *t* test against the normalized control value of 100). (**D**) Representative flow cytometry plots of CD3^+^ T cells treated with increasing concentrations (10–100 ng/mL) of TRAILshort or scrambled peptide, followed by CD3/CD28 stimulation and intracellular p–ZAP-70 staining. (**E**) Paired dot plots showing reduced p–ZAP-70 levels in TRAILshort-treated cells across concentrations (*n* = 7; **P < 0.01, by paired 2-tailed *t* test with Holm-adjusted *P* values). (**F**) CD3^+^ cells treated with TRAILshortΔTm or TRAILshort EVs with or without CD3/CD28 beads were subjected to CD3ζ immunoprecipitation and p–ZAP-70 immunoblotting. (**G**) Western blot analysis of total and p–ZAP-70 in CD3^+^ cells treated with full-length TRAIL EVs, TRAILshort EVs, or both following CD3/CD28 stimulation. Data are presented as the mean ± SEM.

Given the power of large-scale phosphoproteomics as an unbiased, hypothesis-generating tool for exploring signal transduction pathways (29), we used quantitative phosphoproteomics analysis to comprehensively evaluate the effect of TRAILshort on primary T cells. Primary CD4^+^, CD8^+^, and CD3^+^ T cells were treated with TRAILshort peptide or EVs, with or without CD3/CD28 stimulation, and phosphopeptides were quantified across thousands of sites (Figure 2A, Supplemental Figure 2, A and B, and Supplemental Table 2). Kyoto Encyclopedia of Genes and Genomes (KEGG) pathway enrichment analysis revealed significant and reproducible alterations in TCR signaling pathways across independent experiments and T cell populations (Figure 2A and Supplemental Figure 2, A and B), suggesting that TRAILshort broadly affects TCR signaling.

### TRAILshort attenuates ZAP-70 phosphorylation and TCR signaling following CD3/CD28 ligation.

T cells recognize peptide-MHC complexes via the TCR: CD8^+^ T cells engage MHC class I and CD4^+^ T cells engage MHC class II, triggering downstream signaling that drives T cell activation, proliferation, and cytokine production including IFN-γ. A central mediator is ZAP-70 (a TCRζ chain–associated phosphoprotein), whose deficiency abrogates downstream TCR signaling and causes severe combined immunodeficiency in humans (30). Upon TCR ligation, Lck phosphorylates ZAP-70 at Y492/Y493, enabling its interaction with CD3ζ and propagation of downstream signaling (31). This leads to phosphorylation of linker for activation of T cells (LAT) (32), which recruits and activates phospholipase C gamma 1 (PLCγ1) and other signaling effectors (33).

To test whether TRAILshort modulates proximal TCR signaling, primary CD3^+^ T cells were pretreated with TRAILshort peptide or BSA and stimulated with CD3/CD28 beads. CD3/CD28 ligation increased phosphorylated ZAP-70 (p–ZAP-70), p-LAT, and p-PLCγ as expected, whereas TRAILshort peptide alone had no effect. However, TRAILshort pretreatment diminished the phosphorylation of all 3 signaling molecules upon CD3/CD28 stimulation (Supplemental Figure 2C). We then evaluated TRAILshort effects under lower, more physiologically relevant concentrations, consistent with prior studies of immunomodulatory TNF superfamily ligands in disease settings. For example, serum B-cell activating factor levels range from 1–20 ng/mL in patients with autoimmune disorders (34), and soluble TRAIL is present at low ng/mL concentrations in individuals with HIV infection or cancer (35). To assess the effect of reduced TRAILshort concentrations on ZAP-70 phosphorylation, using an appropriate control, T cells were treated with TRAILshort or a scrambled peptide of an equivalent amino acid length prior to CD3/CD28 ligation, and then p–ZAP-70 levels were measured. Exposure to TRAILshort decreased p–ZAP-70 levels compared with levels in scrambled peptide–treated T cells (Figure 2, B and C; *P* < 0.05). Importantly, TRAILshort also attenuated ZAP-70 phosphorylation across a range of lower doses (10–100 ng/mL) relative to matched concentrations of the scrambled control peptide (Figure 2, D and E; *P* < 0.05).

Since TRAILshort is naturally secreted in EV form, we treated CD4^+^ and CD8^+^ T cells with TRAILshort EVs or TRAILshortΔTm EVs, a transmembrane-deficient variant in which TRAILshort is absent from the EV surface and is therefore unable to engage DR5; this construct was used as negative control (Supplemental Figure 1B). TRAILshort EVs suppressed CD3/CD28-induced ZAP-70 phosphorylation in both CD4^+^ and CD8^+^ T cell populations (including effector memory subsets), as assessed by Western blotting and flow cytometry (Supplemental Figure 2, D and E), whereas TRAILshortΔTm EVs did not.

To assess whether TRAILshort disrupts the p–ZAP-70–CD3ζ interaction critical for TCR signal propagation, we performed co-immunoprecipitation following CD3/CD28 stimulation with or without TRAILshort EV treatment. We found that TRAILshort EVs reduced the p–ZAP-70 association with CD3ζ compared with TRAILshortΔTm EVs or untreated controls (Figure 2F). Since physiological EVs probably carry both full-length TRAIL and TRAILshort, we also tested their combined effect on p–ZAP-70; TRAILshort retained its inhibitory activity even in the presence of full-length TRAIL (Figure 2G). Together, these findings demonstrate that TRAILshort suppressed the phosphorylation of proximal TCR signaling molecules, indicating an inhibitory effect on antigen-specific T cell function.

### TRAILshort reduces antigen-specific ELISpot responses, activation, and proliferation in primary T cells.

**Figure 3:**
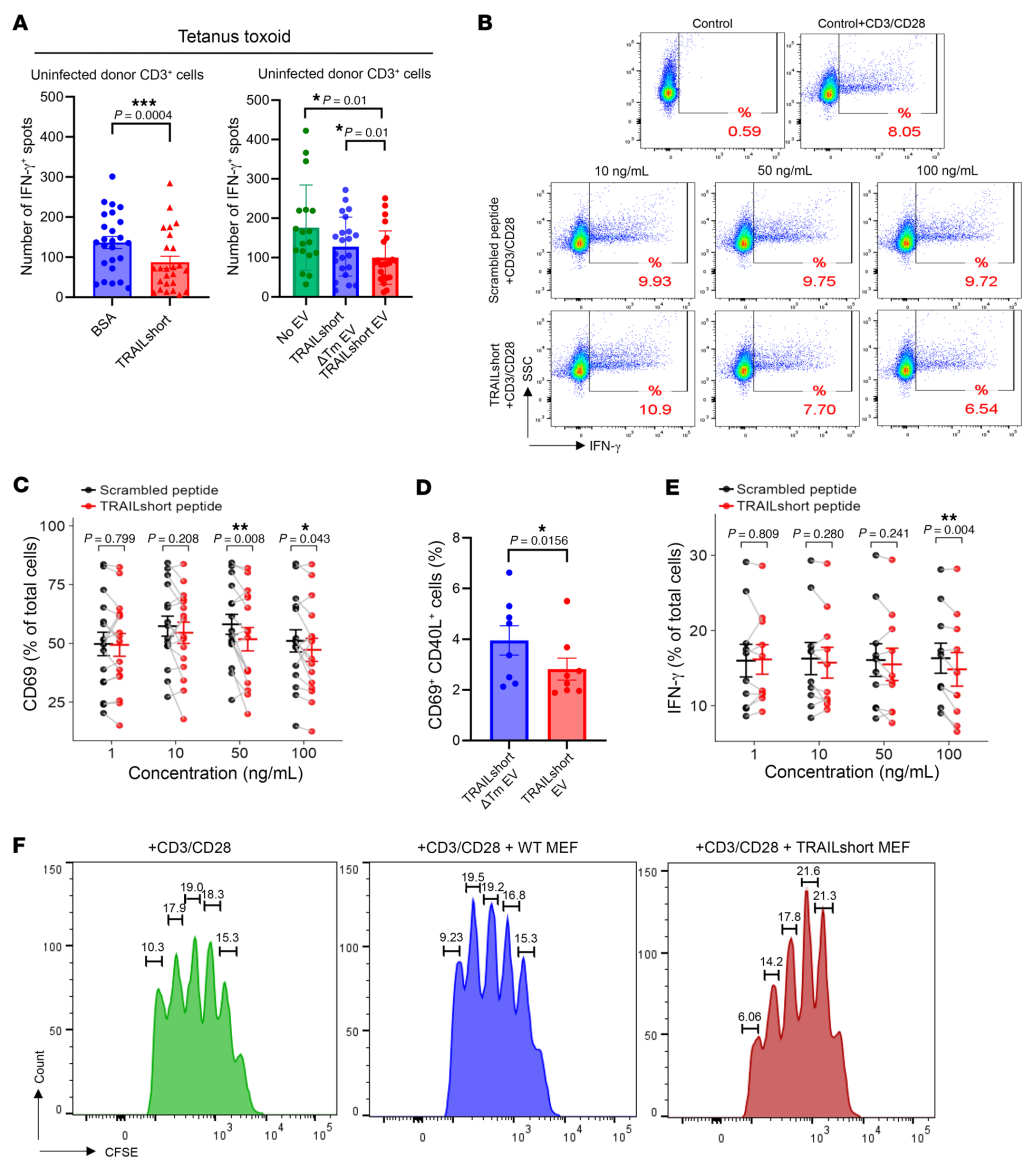
TRAILshort exerts inhibitory effects on the antigen-specific T cell response, cytokine secretion, T cell activation, and primary T cell proliferation. (**A**) IFN-γ ELISpot assays of CD3^+^ T cells from uninfected donors (*n* = 25) stimulated with tetanus toxoid in the presence of TRAILshort peptide or EVs. A Wilcoxon matched-pairs rank test showed that TRAILshort EVs significantly reduced spot formation compared with BSA or TRAILshortΔTm EV controls (*P < 0.05 and ***P < 0.001). (**B**) Flow cytometric analysis of intracellular IFN-γ in T cells treated with increasing doses (10–100 ng/mL) of scrambled or TRAILshort peptide following CD3/CD28 stimulation. (**C**) Quantification showing reduced IFN-γ levels in TRAILshort peptide–treated T cells versus scrambled peptide controls (*n* = 9; *P < 0.05 and **P < 0.01, by paired 2-tailed *t* test with Holm adjustment). (**D**) T cells exposed to EBV gp350 and cocultured with EBV antigen–loaded macrophages were treated with TRAILshort or TRAILshortΔTm EVs, resulting in reduced CD4^+^CD69^+^CD40L^+^ activation markers (*n* = 6; *P < 0.05, by paired *t* test). (**E**) Paired dot plots showing reduced CD69 expression in TRAILshort peptide–treated T cells (10–100 ng/mL) versus scrambled peptide controls (*n* = 15; **P < 0.01, by paired 2-tailed *t* test with Holm adjustment). (**F**) Representative CFSE proliferation histograms showing reduced T cell proliferation in cocultures with TRAILshort-expressing MEFs following CD3/CD28 stimulation. Data are presented as the mean ± SEM.

To examine the effect of TRAILshort on antigen-specific T cell function, we performed ELISpot assays measuring IFN-γ secretion in response to recall antigens. TRAILshort peptide pretreatment reduced IFN-γ–secreting cell frequencies in healthy donor T cells stimulated with tetanus toxoid (Figure 3A; *P* < 0.001) and in T cells from virally suppressed HIV-infected donors stimulated with HIV recall peptides (Supplemental Figure 3A; *P* < 0.01). These suppressive effects were replicated with TRAILshort EVs but not TRAILshortΔTm EVs (Figure 3A; *P* < 0.05; Supplemental Figure 3, A and B; *P* < 0.01). At lower concentrations (1–100 ng/mL), TRAILshort peptide reduced IFN-γ compared with scrambled control at 100 ng/mL (Figure 3, B and C; *P* < 0.01). TRAILshort EV treatment also reduced IL-2 secretion in CD3^+^ T cells cocultured with tetanus toxoid–loaded antigen-presenting cells (APCs) compared with empty EVs (Supplemental Figure 3C; *P* < 0.05).

CD40L and CD69 are rapidly upregulated following antigen-specific T cell activation (36), and treatment with TRAILshort EVs reduced IFN-γ secretion and the proportion of CD4^+^ T cells expressing CD40L and CD69 in response to EBV gp350 (37), compared with TRAILshortΔTm EVs (Figure 3D; *P* < 0.05; Supplemental Figure 3D; *P* < 0.05). TRAILshort peptide (50 and 100 ng/mL) also reduced CD69 expression compared with scrambled controls (Figure 3E; *P* < 0.01 and *P* < 0.05, respectively), confirming that TRAILshort inhibited both cytokine secretion and surface markers of T cell activation.

To assess the effect of TRAILshort on T cell proliferation, CFSE-labeled T cells were stimulated with CD3/CD28 beads in a Transwell coculture system with WT or TRAILshort-knockin mouse embryonic fibroblasts (MEFs) (Supplemental Figure 5B) in the lower chamber, providing continuous EV delivery without direct cell contact. T cells exposed to TRAILshort-containing MEF-derived EVs exhibited delayed proliferation compared with those cocultured with WT MEFs (Figure 3F and Supplemental Figure 3E; *P* < 0.05). Consistent with this, treatment of CD3/CD28-stimulated CD3^+^ T cells with TRAILshort EVs purified from TRAIL-KO HEK293 cells (Supplemental Figure 1A) also reduced proliferation compared with empty EVs or untreated cells (Supplemental Figure 3F; *P* < 0.001).

### Neutralizing TRAILshort with specific antibodies restores TCR signaling and enhances T cell function.

We previously showed that anti-TRAILshort antibodies reverse TRAILshort-mediated resistance to TRAIL-induced apoptosis in HIV-infected CD4^+^ T cells (26, 28), cancer cell lines, and primary B cell malignancies (27). The TRAILshort-specific antibody targets the novel C terminus of TRAILshort which is absent from full-length TRAIL and all other known human proteins (22, 26, 28). Based on our new understanding that TRAILshort functions both to cause TRAIL resistance and T cell hyporesponsiveness, we investigated whether neutralization of TRAILshort using specific antibodies could enhance T cell effector functions during disease states characterized by endogenous TRAILshort production.

Using splenocyte suspensions from patients with primary B cell malignancies — in which malignant B cells express TRAILshort (27) (Supplemental Figure 3G) — we cocultured T cells with autologous TRAILshort-expressing B cells in the presence of anti-TRAILshort antibody or isotype control. Anti-TRAILshort antibody increased IFN-γ secretion across multiple patient groups, including marginal zone lymphoma, diffuse, large B cell lymphoma (DLBCL), and florid lymphoid hyperplasia (Supplemental Figure 3H; *P* < 0.05), consistent with TRAILshort neutralization enhancement of antitumor T cell function.

### TRAILshort binding to DR5 does not trigger apoptosis, necroptosis, NF-κB, or nuclear factor of activated T cells (NFAT) activation.

Full-length TRAIL ligation of death receptors can result in apoptosis, necroptosis (14) or activation of the transcription factors NF-κB and/or NFAT (38). Trimerized TRAIL leads to the clustering of TRAIL death receptors, which in turn recruits Fas-associated protein with death domain (FADD) to the death domain of DR5, forming death-inducing signaling complex (DISC, which subsequently recruits pro–caspase 8, causing its autoactivation into caspase 8 (21, 39). Furthermore, trimerized TRAIL binding to DR5 can trigger necroptosis by activating receptor-interacting protein kinase 1 (RIPK1) and receptor-interacting protein kinase 3 (RIPK3) (14).

**Figure 4:**
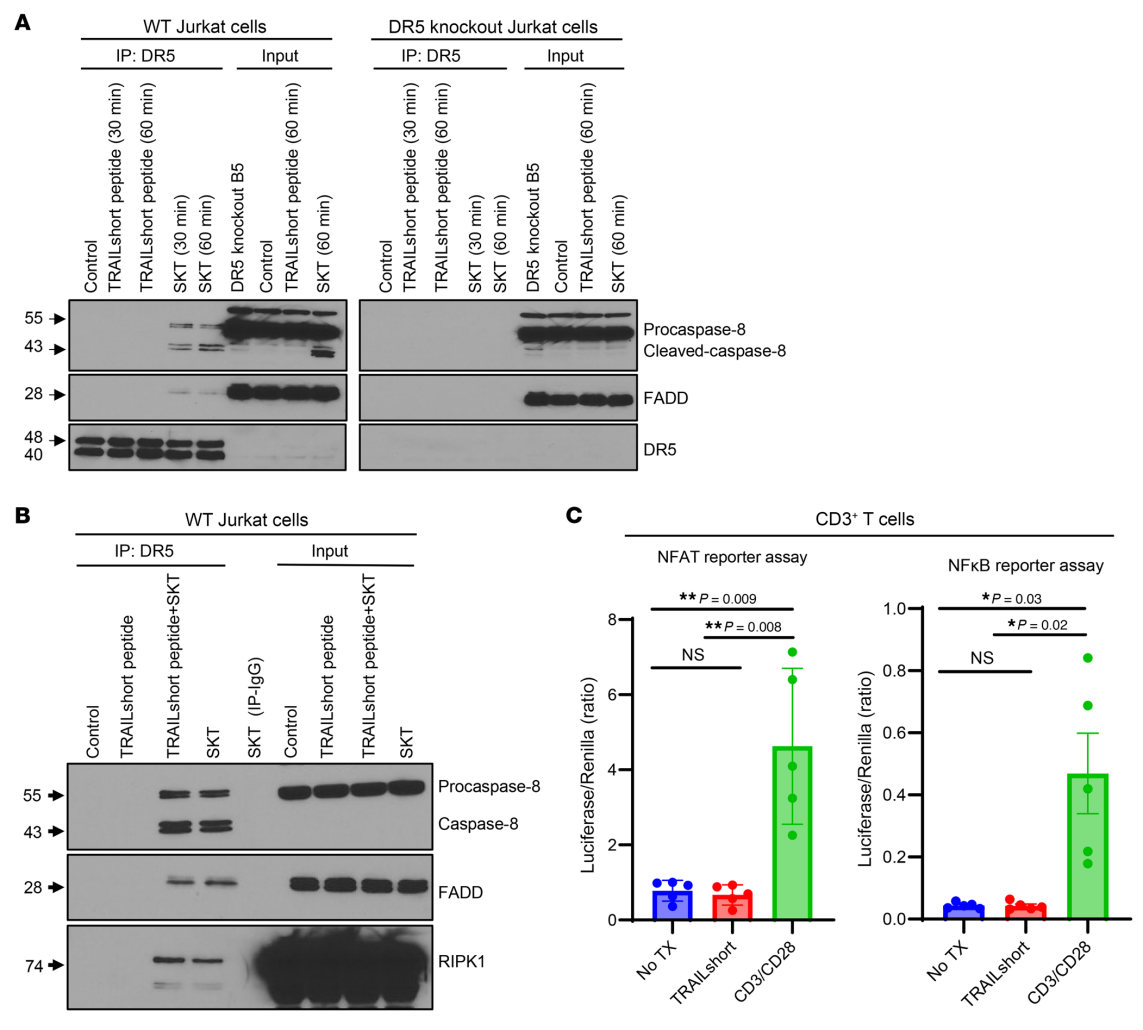
TRAILshort does not induce apoptosis, necroptosis, or NF-κB or NFAT activation in T cells. (**A**) WT or DR5-deficient Jurkat cells were treated with TRAILshort peptide or super killer TRAIL (SKT) for 30 or 60 minutes. DR5 immunoprecipitated lysates were immunoblotted for FADD, caspase 8, and DR5. FADD recruitment was detected with SKT but not TRAILshort. (**B**) Jurkat cells treated with SKT, TRAILshort, or both were subjected to DR5 immunoprecipitation. Caspase 8, FADD, and RIPK1 associated with DR5 following SKT but not TRAILshort treatment. (**C**) NF-κB and NFAT reporter activities were measured in primary CD3^+^ T cells following TRAILshort treatment. CD3/CD28 stimulation served as a positive control. *P < 0.05 and **P < 0.01, by 2-tailed, paired *t* test with Holm adjustment. Paired *t* test analysis showed a significant increase between untreated and TRAILshort-treated groups compared with CD3/CD28-stimulated groups (*P < 0.05). TRAILshort alone slightly reduced NFAT reporter activity. Data are presented as the mean ± SEM.

To determine whether TRAILshort-mediated T cell suppression occurs through apoptosis or necroptosis of T cells, we evaluated DISC formation, caspase 8/FADD recruitment, and RIPK1/MLKL phosphorylation. TRAILshort neither induced apoptosis or necroptosis, nor promoted DISC assembly (Figure 4, A and B, and Supplemental Figure 4, A and B), indicating that TRAILshort-mediated T cell hyporesponsiveness was independent of cell death and these pathways. TRAILshort also did not activate NF-κB, but slightly reduced NFAT signaling (Figure 4C).

### TRAILshort binding to DR5 recruits SHP-1.

**Figure 5:**
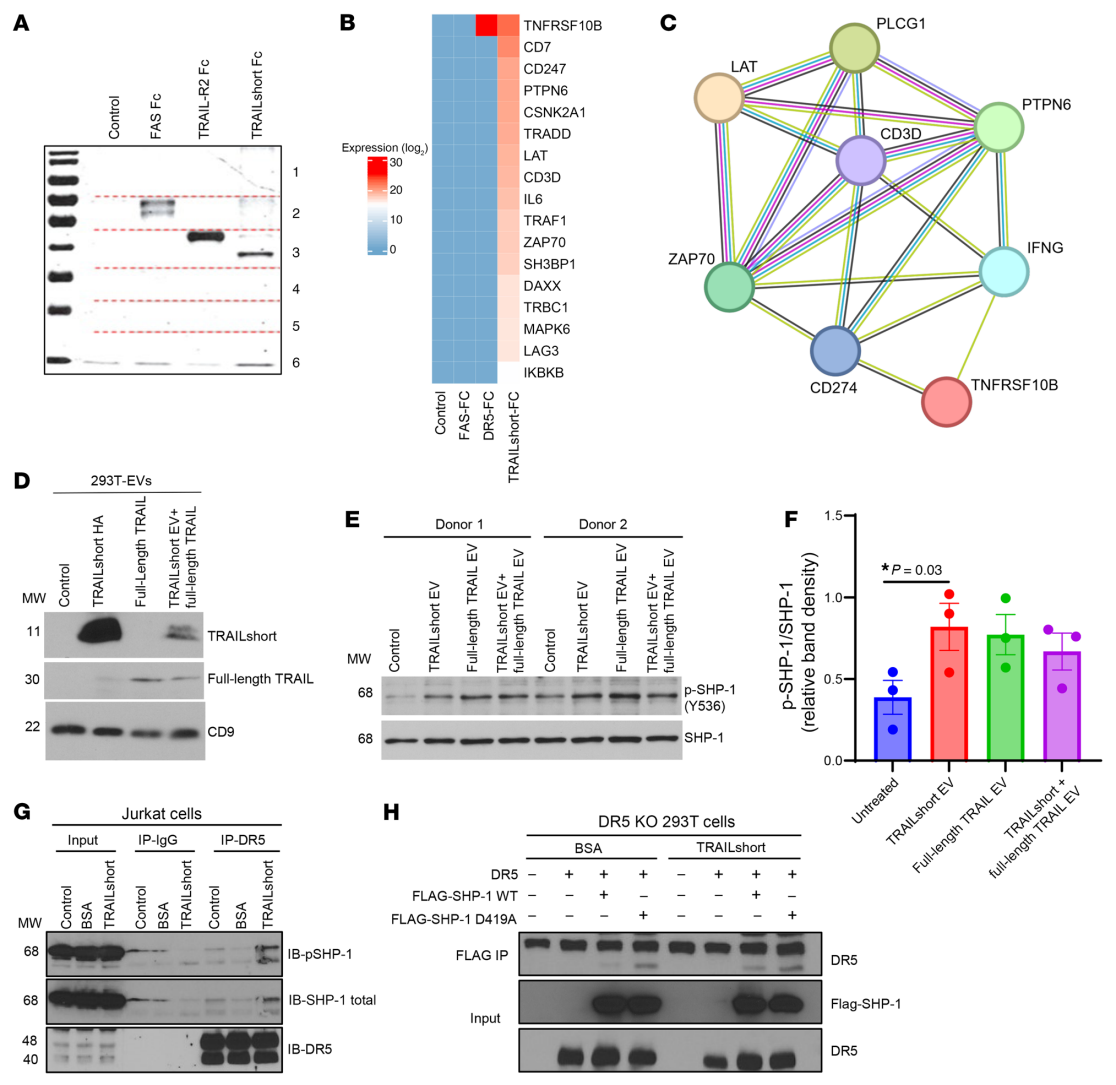
TRAILshort treatment induces the recruitment and activation of SHP-1 at the DR5 receptor. (**A**) CD3^+^ T cell lysates were immunoprecipitated with FAS-Fc, DR5-Fc, or TRAILshort-Fc, resolved by SDS-PAGE, and analyzed by in-gel digestion and proteomics. (**B**) Proteomics analysis identified DR5, ZAP-70, and SHP-1 as proteins pulled down by TRAILshort-Fc. (**C**) STRING (Search Tool for the Retrieval of Interacting Genes/Proteins) analysis revealed interactions among DR5, SHP-1, and TCR signaling molecules associated with TRAILshort-Fc. (**D**) Western blot showing full-length TRAIL and TRAILshort in EVs isolated from 293T cells transfected with TRAIL or TRAILshort constructs. (**E**) Western blot analysis of T cells from 2 healthy donors treated with purified EVs (**D**) at a 1:1,000 cell-to-EV ratio and probed for total SHP-1 and p–SHP-1 (Y536). (**F**) Western blot band quantification demonstrated increased p–SHP-1 expression in cells treated with TRAILshort EV (*P < 0.05, by paired, 2-tailed *t* test with Holm adjustment). Data are presented as the mean ± SEM. (**G**) Jurkat cells were treated with no stimulus, BSA, or TRAILshort, followed by immunoprecipitation with DR5 or isotype control antibodies. Immunoblotting for p–SHP-1 and total SHP-1 showed increased DR5-associated p–SHP-1 after TRAILshort treatment. (**H**) DR5-KO 293T cells were transfected with DR5 alone, DR5 plus Flag-tagged WT SHP-1, or Flag-tagged mutant SHP-1 (D419A). Cells were treated with TRAILshort peptide or BSA, followed by anti-Flag immunoprecipitation. Immunoblotting shows mutant SHP-1 (D419A) associated with DR5 under both conditions, whereas WT SHP-1 interacted with DR5 only after TRAILshort treatment, consistent with TRAILshort-induced SHP-1 recruitment and activation through DR5.

To identify mechanisms by which TRAILshort impairs TCR signaling, we performed Fc fusion pull-down assays using TRAILshort-Fc, Fas-Fc, and DR5-Fc constructs, followed by mass spectrometry. ZAP-70, CD3ζ, and SHP-1 (also known as protein tyrosine phosphatase nonreceptor type 6 [PTPN6]) were uniquely identified as TRAILshort-interacting proteins (Figure 5, A–C, and Supplemental Table 3). DR5 was detected in both the TRAILshort-Fc and DR5-Fc pull-downs; its presence in the TRAILshort-Fc assay further supported preferential TRAILshort-DR5 binding (22, 26), while in the DR5-Fc assay, it likely reflected the input fusion protein.

SHP-1, also known as PTPN6, is a cytoplasmic tyrosine phosphatase expressed predominantly in hematopoietic cells that acts as a negative regulator of T cell, B cell, and NK cell signaling (40). In T cells, SHP-1 dephosphorylates ZAP-70, CD3ζ, and linker for activation of T cells (LAT), thereby dampening TCR signaling, whereas SHP-1–deficient thymocytes display prolonged TCR-induced phosphorylation, enhanced proliferation, and increased ZAP-70 activity (41). SHP-1 is autoinhibited in its basal state through intramolecular N-terminal SH2-phosphatase domain interactions (42) and is activated upon engagement with immunoreceptor tyrosine-based inhibitory motif-containing receptors or immunoreceptor tyrosine-based switch motif-containing receptors, leading to autophosphorylation at Y536 (43). Death receptors of the TNF receptor (TNFR) family, including Fas, can recruit and activate SHP-1 via cytoplasmic phosphotyrosine motifs (44, 45), although DR5-specific SHP-1 engagement in human T cells had not been fully defined. Our identification of TRAILshort–DR5–SHP-1 complex formation (Figure 5, A–C) led us to hypothesize that TRAILshort binding to DR5 recruits and activates SHP-1, thereby antagonizing T cell receptor (TCR) signaling.

Both TRAILshort peptide (100–5000 ng/mL) and TRAILshort EVs induced SHP-1 phosphorylation in T cells at Y536 (Supplemental Figure 4, C and D). To model a physiological immune microenvironment containing TRAILshort and full-length TRAIL, we generated EVs from 293T cells transfected with TRAILshort, full-length TRAIL, or both (Figure 5D). Primary T cells treated with all 3 EV conditions exhibited significant SHP-1 phosphorylation (Figure 5, E and F; *P* < 0.05), demonstrating that TRAILshort-driven SHP-1 activation was preserved in the presence of full-length TRAIL. Notably, EVs containing full-length TRAIL alone were also sufficient to induce SHP-1 phosphorylation, indicating that SHP-1 activation via DR5 was not exclusive to TRAILshort but rather represents a shared signaling property of the TRAIL/DR5 axis.

Fas recruits and activates SHP-1 via a cytoplasmic YxxL motif (44). Since DR5 harbors a similar YxxL motif at position 383, we hypothesized that TRAILshort binding to DR5 recruits SHP-1 to this domain, triggering its activation. To test this idea, Jurkat T cells were treated with TRAILshort peptide, DR5 was immunoprecipitated, and the precipitate was probed for total and p–SHP-1 (Y536) levels. SHP-1–DR5 interaction was detected exclusively in TRAILshort-treated cells, and the associated SHP-1 was predominantly in the phosphorylated (activated) form, indicating that TRAILshort engagement of DR5 recruited and activated SHP-1 (Figure 5G).

To further assess the putative interaction of SHP-1 with TRAILshort and DR5, we constructed expression vectors containing the Flag-tagged WT SHP-1 and a mutant SHP-1 with an alanine substitution at aspartic acid 419 (D419A). This substitution stabilizes the interaction of SHP-1 with its binding partners and is referred to as a substrate-trapping mutant (46). These constructs were coexpressed with the DR5 in 293T cells lacking the DR5 gene, and the cells were subsequently treated with TRAILshort peptide and subjected to immunoprecipitation using an anti-Flag antibody. Our results revealed that the substrate-trapping mutant of SHP-1 was associated with DR5 in both BSA- and TRAILshort-treated cells, but that WT SHP-1 was associated with DR5 only when pretreated with TRAILshort (Figure 5H).

### Blocking SHP-1 reverts the TRAILshort-induced inhibition of TCR signaling.

**Figure 6:**
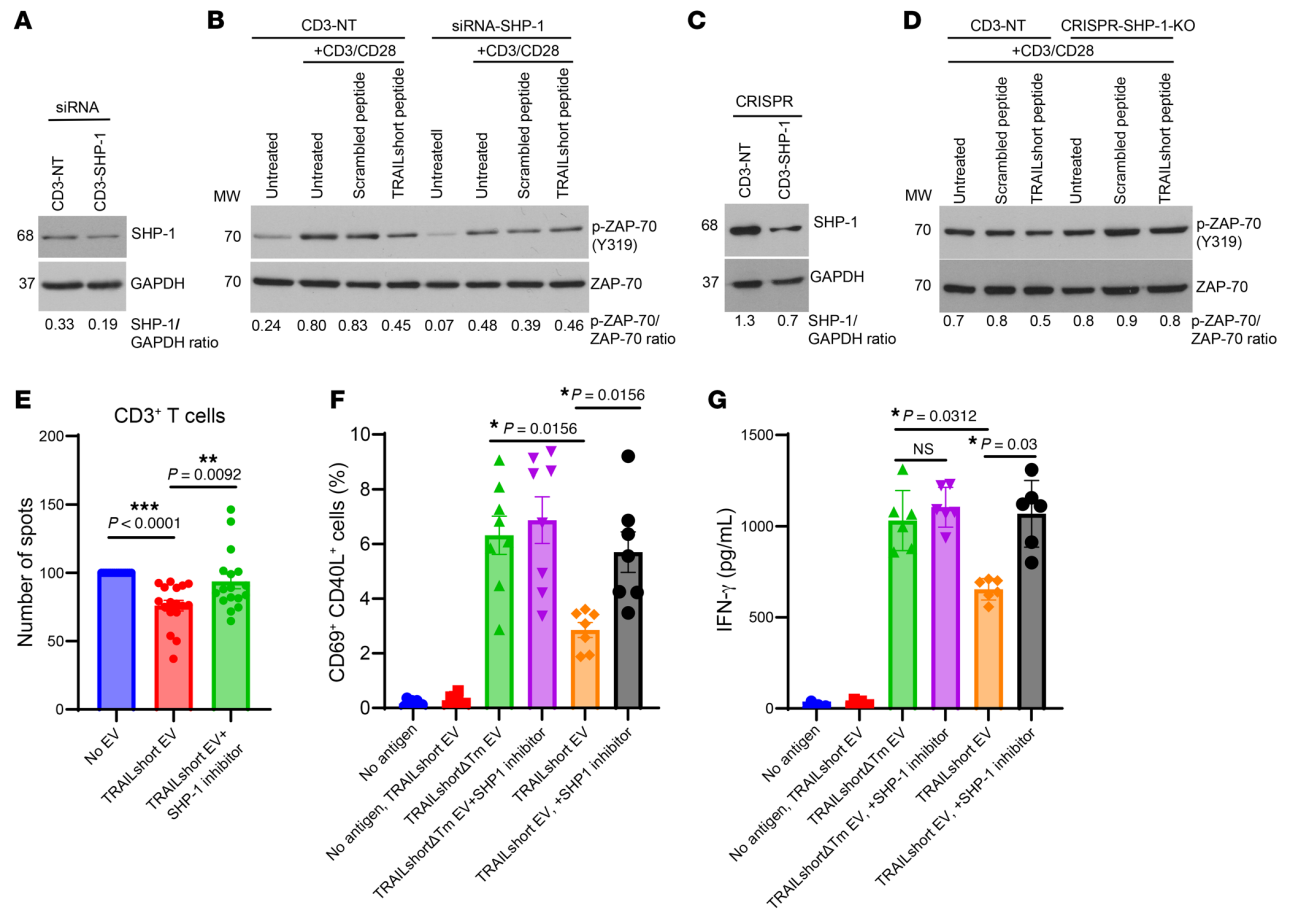
SHP-1 inhibition reverses TRAILshort-mediated suppression of T cell function. (**A**–**D**) Primary CD3^+^ T cells were transfected with nontargeting (NT) or SHP-1 siRNA to reduce SHP-1 expression. Cells were then pretreated with scrambled peptide or TRAILshort peptide and stimulated with CD3/CD28 beads (1:2 bead-to-cell ratio) for 30 minutes. (**A** and **B**). Alternatively, SHP-1 was knocked out using CRISPR/Cas9 and under the same experimental conditions (**C** and **D**). Western blotting was performed to assess total and phosphorylated ZAP-70. (**E**) Primary CD3^+^ T cells were analyzed by IFN-γ ELISpot analysis in response to recall antigen tetanus toxoid, either alone, in the presence of TRAILshort, or with TRAILshort plus the SHP-1 inhibitor (0.5 μM), showing that SHP-1 inhibition restored IFN-γ spot formation suppressed by TRAILshort. **P < 0.01 and ****P < 0.0001, by 1-sample *t* test against the normalized control value of 100%) for No EV versus TRAILshort EV, and 2-tailed, paired *t* test for TRAILshort EV versus TRAILshort EV plus the SHP-1 inhibitor. *P* values were adjusted for multiple comparisons using the Holm method. (**F** and **G**) PBMCs were exposed to EBV gp350 for 5 days and then cocultured with syngeneic EBV antigen–loaded macrophages and treated with either TRAILshort or TRAILshortΔTm EVs, alone or with the SHP-1 inhibitor. The proportion of CD4^+^CD69^+^CD40L^+^ T cells was assessed by flow cytometry (**F**), and IFN-γ secretion was measured by ELISA (**G**), demonstrating that SHP-1 inhibition reversed TRAILshort-mediated reductions in T cell activation and cytokine production. *P < 0.05, **P < 0.01, and ****P < 0.001, by Wilcoxon matched-pairs, signed-rank test for comparisons between 2 groups. data are presented as the mean ± SEM.

Next, we evaluated whether SHP-1 inhibition could reverse TRAILshort-mediated suppression of TCR signaling using 3 independent approaches: (a) a pharmacologic SHP-1 inhibitor, (b) CRISPR-mediated SHP-1 KO, and (c) siRNA-mediated SHP-1 knockdown. All 3 strategies restored ZAP-70 phosphorylation following TCR ligation in the presence of TRAILshort (Figure 6, A–D, and Supplemental Figure 4, E and F). Moreover, SHP-1 inhibition increased IFN-γ secretion by T cells in ELISpot assays (Figure 6E) and enhanced both the frequency of activated CD69^+^CD40L^+^CD4^+^ T cells and IFN-γ secretion in TRAILshort-treated T cells exposed to EBV antigen (Figure 6, F and G). Additionally, SHP-1 inhibition promoted the proliferation of CD3^+^ T cells in response to TRAILshort EVs (Supplemental Figure 4G). The pharmacological inhibition of SHP-1 produced only a modest reversal of TRAILshort-induced suppression of ZAP-70 phosphorylation, in contrast to the more pronounced effects observed with CRISPR-mediated KO or siRNA knockdown of SHP-1. This difference is most consistent with the limited potency and specificity of the small-molecule inhibitor, which targets SHP-1 as well as related phosphatases such as SHP-2, thereby constraining its ability to fully restore TCR signaling and effector functions (47). These findings collectively underscore the pivotal role of SHP-1 in modulating the effects of TRAILshort on T cell function.

### MEFs derived from TRAILshort-knockin mice are resistant to TRAIL-induced apoptosis but remain sensitive to Fas-mediated cell death.

Since murine TRAIL genomic organization precludes TRAILshort generation, we generated a TRAILshort-knockin mouse using a Cre-LoxP strategy (Supplemental Figure 5A). MEFs derived from WT or homozygous TRAILshort embryos were transformed with SV40 large T antigen and transduced with a luciferase-GFP vector; TRAILshort expression and EV-associated secretion were confirmed by immunofluorescence and Western blotting (Supplemental Figure 5, B and C). When treated with murine super killer TRAIL or anti-Fas antibody and monitored by live-cell caspase 3/-7 imaging over 40 hours, we observed that TRAILshort MEFs were resistant to TRAIL-induced apoptosis (*P* < 0.01) but remained sensitive to Fas-mediated apoptosis (Supplemental Figure 5, D and E).

### Evaluating the effect of TRAILshort in vivo.

**Figure 7:**
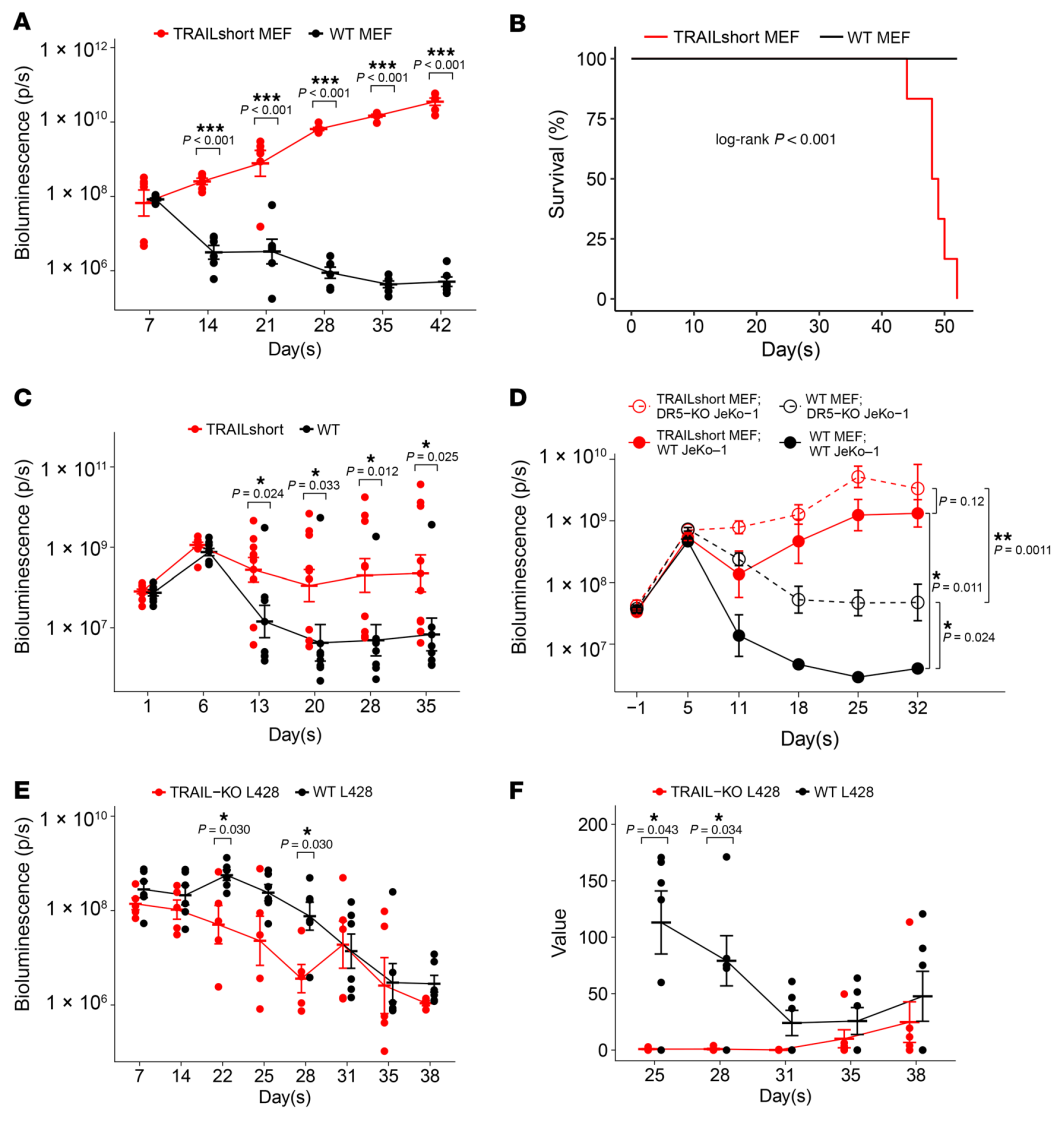
Effect of TRAILshort in vivo. (**A**) Luciferase-expressing immortalized WT or TRAILshort MEFs were implanted s.c. into Hu-CD34^+^ NSG mice (6 mice/group), and tumor growth was monitored weekly by bioluminescence imaging, with flux measured in photons/second (p/s). *P* values were calculated using 2-sample *t* tests on log-transformed flux at each prespecified time point. (**B**) Survival of humanized CD34^+^ mice implanted with WT or TRAILshort MEFs. WT MEFs showed limited expansion, whereas TRAILshort MEFs displayed increased and sustained bioluminescent signal. *P* < 0.001, by log-rank (Mantel-Cox) test. (**C** and **D**) NSG mice were s.c. implanted on both flanks with WT or TRAILshort MEFs (non-luciferase-expressing), followed by i.v. injection of luciferase-expressing JeKo-1 cells. Mice were treated with CD19 CAR-T cells, and JeKo-1 tumor growth was monitored weekly by bioluminescence imaging. TRAILshort MEFs impaired CD19 CAR-T–mediated control of both WT and DR5-KO JeKo-1 tumors compared with WT MEFs. *P* values were calculated using (**C**) 2-sample *t* tests on log-transformed flux at each prespecified time point and (**D**) Tukey’s honestly significant difference (HSD) pairwise comparisons on log-transformed flux at the final time point. (**E** and **F**) WT and TRAIL/TRAILshort-KO L428 cells were implanted s.c. into Hu-CD34^+^ NSG mice (6 mice/group), and tumor growth was monitored for 38 days by bioluminescence imaging. TRAIL/TRAILshort-deficient L428 tumors showed lower total flux than did WT L428 tumors at days 7 and 28 (**E**), and caliper measurements beginning on day 25 (**F**) likewise demonstrated reduced tumor volume in the TRAIL/TRAILshort-KO group. *P < 0.05, **P < 0.01, and ***P < 0.001, by 2-sample *t* test on log-transformed flux at each time point (**E**) and Wilcoxon rank-sum test on tumor volume at each time point (**F**). Data are presented as the mean ± SEM.

To evaluate the effect of TRAILshort on human T cell function in vivo, we used 2 complementary mouse models with transformed MEFs derived from either WT or TRAILshort-knockin mice. In this system, tumors required several weeks to reach a reliably measurable size, allowing differences driven by partial, sustained impairment of T cell function — rather than complete immune failure — to emerge and accumulate progressively over time. In Hu-CD34^+^ NOD scid gamma mice bearing a functional human immune system and engrafted with luciferase^+^ MEFs, WT MEFs were progressively controlled, whereas TRAILshort-expressing MEFs showed greater early expansion and higher bioluminescent signal, indicating that MEF-derived TRAILshort dampened human T cell–mediated clearance (Figure 7, A and B, and Supplemental Figure 6, A–C).

We next adapted an established NSG CD19 CAR-T/JeKo-1 mantle cell lymphoma model (48) by s.c. preimplanting WT or TRAILshort MEFs (not expressing luciferase) prior to i.v. injection of luciferase-labeled CD19^+^ JeKo-1 cells, followed by CD19 CAR-T cells (Supplemental Figure 6D). This model allowed us to determine if TRAILshort supplied by MEFs affect CAR-T control of tumors, and by implanting the tumors and MEFs only, it allowed us to determine if TRAILshort-expressing MEFs directly affect tumor cells, independent of their effects on T cells. Because TRAILshort MEFs release TRAILshort-containing EVs, any immunosuppressive effects of TRAILshort in this setting would be expected to act systemically rather than being confined to the local implantation site. In this system, WT MEFs did not impair CAR-T–mediated control of WT JeKo-1 cells, nor did TRAILshort MEFs alter JeKo-1 growth in the absence of CAR-T cells (Figure 7C and Supplemental Figure 6, E and F), indicating that MEF-derived TRAILshort did not affect lymphoma cell outgrowth and that WT MEF did not impair CAR-T control of tumors.

As expected, CD19 CAR-T cells efficiently eliminated WT JeKo-1 tumors in the presence of WT MEFs, and because CAR-T–mediated killing is in part driven by TRAIL-DR5 interactions (49, 50), this killing was attenuated when DR5 was deleted in JeKo-1 cells (Figure 7D). In the presence of TRAILshort MEFs, CD19 CAR-T–mediated killing of WT JeKo-1 cells was reduced compared with WT MEFs (*P* = 0.0012), and CD19 CAR-T–mediated killing of DR5-KO JeKo-1 cells was likewise diminished in mice bearing TRAILshort MEFs compared with mice with WT MEFs (Figure 7D; *P* < 0.01). Together, these results indicate that MEF-derived TRAILshort impaired CAR-T effector function rather than acting by making JeKo-1 tumor cells themselves less sensitive to DR5-dependent cytotoxicity.

To assess the effect of TRAILshort using an in vivo humanized tumor model, we used L428 Hodgkin lymphoma cells, which endogenously express high levels of TRAILshort (Supplemental Figure 7), in the same Hu-CD34 NSG model used in Figure 7, A and B, to mimic a native human immune system. Using CRISPR/Cas9, we generated TRAIL-KO L428 cells, in which both full-length TRAIL and TRAILshort were deleted, as TRAILshort is encoded within the same gene and cannot be selectively ablated. Equal numbers of WT L428 cells and TRAIL- and TRAILshort-deficient L428 cells were implanted on day 1, and tumor burden was quantified longitudinally by bioluminescence every 7 days. Over the course of 4 weeks, TRAIL/TRAILshort-deficient L428 tumors maintained lower total flux values than did WT L428 tumors at days 7 and 28 (Figure 7, E and F; *P* < 0.05), reflecting impaired tumor expansion in the absence of the immunosuppressive effects of TRAILshort. By day 28, both WT and TRAIL/TRAILshort-deficient tumors were controlled, suggesting that TRAILshort primarily delays, rather than prevents, immune-mediated tumor clearance in this setting. It is not surprising that deletion of TRAILshort alone did not lead to tumor eradication, as additional oncogenic and tumor-intrinsic survival pathways remained intact.

## Discussion

TRAIL-mediated apoptosis of virally infected cells is well established. HIV-infected T cells upregulate DR4 and DR5 (51) and are more susceptible to TRAIL-induced death, with TRAIL also implicated in bystander CD4^+^ T cell depletion in humanized HIV models (52). In murine influenza infection, TRAIL expressed by NK and T cells promotes viral clearance, and TRAIL or TRAIL receptor deficiency worsens outcomes (53). Similar selective killing of infected cells by TRAIL has been reported for CMV, respiratory syncytial virus (RSV), and reovirus (54–56).

Beyond antiviral immunity, TRAIL/TRAIL receptor signaling contributes to tumor immunosurveillance: loss or blockade of TRAIL or its receptors accelerates tumor development and metastasis in mouse models (57), and tumor-infiltrating lymphocytes show elevated TRAIL expression (58). In human cancers, mutations in key TRAIL pathway components suggest that escaping TRAIL-mediated apoptosis may promote tumor development and progression (59). Recombinant TRAIL and agonistic TRAIL antibodies were developed as cancer therapies with favorable safety profiles in early trials, but limited antitumor efficacy precluded registrational studies (60), potentially because of paradoxical protumor effect (61). Efforts to boost TRAIL agonist potency with preclustered hexameric agonists such as eftozanermin alfa (ABBV-621) and APG350 have been pursued, but eftozanermin showed hepatotoxicity and only modest clinical response rates in phase I (62), indicating that additional resistance mechanisms, potentially including TRAILshort, must also be involved.

Our findings reveal what we believe to be a new dimension of TRAILshort biology: it inhibits antigen-specific T cell activation and suppresses TCR-mediated proliferation and cytokine production within the microenvironment where it is expressed. Immune escape is a central driver of disease progression in both chronic infection and cancer, enabling pathogen and tumor survival, while complicating treatment (63). Here, we provide evidence that TRAILshort contributed to immune escape by functionally disabling T cells — a significant finding, given that tumor-infiltrating T cells are favorable prognostic markers in cancer (64) and that defective HIV-specific T cell activation and cytokine production in lymphoid tissues impair viral reservoir clearance (65). These findings add a new regulatory layer to T cell activity within tumor niches and HIV-infected tissues and highlight how alternative splicing of TRAIL extends the functional repertoire of the TRAIL system beyond apoptosis. Mechanistically, death receptors have previously been linked to T cell regulation — Fas crosslinking inhibits CD3 signaling via SHP-1 recruitment (66), and TRAIL ligation recruits SHP-1 to the murine TRAIL receptor in T cells (45), but whether this occurs in primary human T cells remained undefined. The relationship between SHP-1 and TRAIL receptors has been less well defined. In hepatocellular cancer cell lines, treatment with sorafenib and dovitinib can convert TRAIL-resistant cells into TRAIL-sensitive ones, an effect that is blocked by SHP-1 inhibition (67, 68). We now demonstrate that in primary human T cells, TRAILshort binding to DR5 recruited and activated SHP-1, as confirmed by unbiased pulldown and reciprocal immunoprecipitation assays, and that SHP-1 inhibitors, or siRNA, or CRISPR depletion of SHP-1 reversed TRAILshort-mediated suppression of T cell activation and cytokine secretion. These data support a model in which TRAILshort-DR5 engagement activates SHP-1, which dephosphorylates ZAP-70 and impairs its association with CD3ζ, thereby disrupting proximal TCR signaling.

Our data add a new layer to the emerging view of TRAIL as protumorigenic, identifying TRAILshort as contributing to that protumorigenic effect by attenuating the cytotoxic potential of full-length TRAIL and delivering an immunosuppressive mechanism within the microenvironment. Together with survival-promoting NF-κB and stress kinase pathway signaling by full-length TRAIL in tumor cells, the additive effect of TRAILshort-mediated immune suppression may collectively define the net protumorigenic effect of the TRAIL system in human disease, as evidenced by the persistence of TRAILshort-expressing MEFs and Hodgkin lymphoma L428 xenografts in humanized mice and impaired CAR-T cell control of JeKo-1 lymphoma in the presence of TRAILshort-expressing stromal cells.

Prior work suggested that TRAIL ligation can dampen TCR signaling (69), but whether this is mediated by full-length TRAIL, TRAILshort, or both remains unclear. Our data demonstrate that TRAILshort mediated SHP-1–dependent inhibition of proximal TCR signaling. While full-length TRAIL may share this downstream ability (Figure 5, E and F), its systemic delivery was constrained by apoptotic, necroptotic, and NF-κB effects not observed with TRAILshort.

The identification of TRAILshort as a DR5 dependent, SHP-1–mediated inhibitor of proximal TCR signaling has several implications for immunotherapy design in malignancy and chronic infection. In cancer, TRAILshort expression by tumor cells is predicted to blunt endogenous and engineered T cell responses, including CAR-T cells at sites of disease. These findings suggest that TRAILshort blockade, using antibodies that selectively recognize its unique C-terminus, could be combined with CAR-T cells, immune checkpoint inhibitors, or therapeutic cancer vaccines to enhance antigen-specific T cell activity without globally augmenting systemic inflammation. In HIV and other chronic viral infections, TRAILshort is induced in response to type I IFNs and TLR7/-8/-9 agonism and is enriched in tissues characterized by persistent antigen and immune dysfunction. Within these microenvironments, TRAILshort would be expected to dampen recall responses to viral antigens and impair clearance of infected cells, as supported by the observed reduction in IFN-γ and IL-2 secretion and T cell proliferation in the presence of TRAILshort. In this setting, TRAILshort neutralization might synergize with latency-reversing agents, therapeutic vaccination, or broadly neutralizing antibodies to overcome local T cell hyporesponsiveness and improve reservoir targeting.

Conversely, expression or localized delivery of TRAILshort may offer a strategy to induce tissue-restricted T cell hyporesponsiveness in settings in which immune activation is pathogenic. In allogeneic transplantation, gene-modified grafts engineered to express TRAILshort could promote engraftment by attenuating rejection, potentially reducing the need for systemic immunosuppression. Similarly, in autoimmune diseases characterized by deficient TRAILshort expression, selective restoration through localized TRAILshort delivery may reinforce immune tolerance at sites of pathogenic T cell activation. Together, these observations position TRAILshort as both a therapeutic target and a potential tolerogenic tool, depending on disease context.

## Methods

### Sex as a biological variable

This study examined freshly isolated primary human T cells from both female and male individuals, and similar findings were reported for both sexes. Female mice were used exclusively in all animal experiments to avoid sex bias. No evidence from our previous study indicated that sex influences the key functions or molecular mechanisms of TRAILshort.

### Cell lines

HEK293, JeKo-1, Jurkat, and L428 cell lines were obtained from the American Type Culture Collection (ATCC) and cultured in DMEM (HEK293) or RPMI (JeKo-1, Jurkat, and L428) supplemented with 10% FBS, 1% penicillin-streptomycin, and 1% l-glutamine. All cell lines were routinely tested for mycoplasma contamination.

### RNA-seq data analysis

TRAILshort (ENST00000420541) expression enrichment was analyzed using the RNA Explorer tool (nference). Over 400,000 human RNA-Seq samples from GEO were processed against the GRCh38 reference using fastp (70, 71) for preprocessing and salmon for alignment-free quantification with gc and seq bias correction. Sample phenotyping was performed via nference’s NLP tools on the basis of associated metadata. Samples were stratified into high (top 5%) and low (bottom 5%) TRAILshort expressers by transcripts per million, and phenotype differences between groups were assessed by hypergeometric test with Benjamini-Hochberg correction.

### EV uptake assay

WT or DR5-KO 293T cells were cultured overnight and then incubated with GFP-TRAILshort EVs labeled with anti-TRAILshort antibody conjugated to pHrodo pH-sensitive dye (Invitrogen, Thermo Fisher Scientific). Images were captured every 2 hours over a 12-hour period by LSM780 multiphoton confocal microscopy (Zeiss). EV internalization was quantified by the red-to-green pixel ratio (MATLAB) in the acidic intracellular environment, with graphs generated in GraphPad Prism 9 (GraphPad Software).

### Antibodies and peptides

The antibodies and peptides used in this study are listed in Supplemental Table 4.

### Preparation of TRAILshort, TRAILshortΔTm, and empty EVs

TRAIL-KO HeLa cells were transfected with pRuby, pRuby-TRAILshort, or pRuby-TRAILshortΔTm (transmembrane-deficient variant) using polyethylenimine (MW 25,000 Da; Polysciences). Separately, 293T TRAIL-KO cells were transfected with TRAILshort-HA and/or full-length TRAIL-Flag constructs or control vectors. All transfections used 15 μg cDNA and 30 μg PEI in complete DMEM. At 24 hours, media were replaced with serum-free DMEM; conditioned media were collected at 48 and 72 hours, cleared by centrifugation (2,578*g*), 30 minutes), and concentrated via tangential flow filtration (100 kDa MW cutoff [MWCO]) to 30 mL. EVs were purified by RION Inc., resuspended in sterile double-distilled water (ddH_2_O) and stored at –80°C.

### Primary T cell isolation

Apheresis cones were obtained from uninfected or HIV^+^ donors. Primary CD3^+^, CD4^+^, CD8^+^, and/or CD19^+^ cells were isolated from apheresis blood or PBMCs by negative selection (STEMCELL Technologies) and cultured in RPMI with 10% FBS, 1% penicillin-streptomycin, and 1% glutamine. Bulk splenocytes from splenectomy specimens obtained from patients with hematologic malignancies were similarly separated into T and B cell populations by negative selection.

### Mass spectrometry–based phosphoproteome profiling

Primary CD3^+^ T cells were stimulated with CD3/CD28 beads (1:1, 48 hours), serum starved (1% FBS, 6 hours), and incubated on BSA- or TRAILshort peptide–coated plates for 5, 15, or 30 minutes before collection for phosphoproteomics profiling. Beads were resuspended in 2 M urea/50 mM triethylammonium bicarbonate, reduced with 10 mM DTT, alkylated with 40 mM iodoacetamide, and digested overnight with trypsin (1 μg, 37°C). Digests were acidified with 20% trifluoroacetic acid and desalted via C18 stage tip. One-quarter of the peptides were reserved for global proteome profiling; the remainder underwent phosphopeptide enrichment using Fe(III)-NTA resin (Agilent Technologies) on an AssayMap Bravo platform (30). Phosphotyrosine peptides were further enriched using anti-pY magnetic beads (PTMScan HS pY1000, Cell Signaling Technology) in immunoaffinity purification buffer (4°C, 1 hour), washed, and eluted with 0.15% TFA.

Liquid chromatography tandem mass spectrometry (LC-MS/MS) was performed on an Orbitrap Eclipse Tribrid mass spectrometer coupled to an Ultimate 3000 LC system (Thermo Fisher Scientific). Peptides were loaded onto a PepMap C18 trap column and separated over 100 minutes on a PepMap C18 EASY-Spray column (75 μm × 50 cm) (both from Thermo Fisher Scientific). Data were acquired in data-dependent mode at 120,000/30,000 resolution (MS/MS^2^), with 1.2 Th precursor isolation and NCE of 28. Raw data were searched using Andromeda/MaxQuant (version 1.6.17.0) against the UniProt human proteome (20,609 entries), allowing 2 missed cleavages, fixed carbamidomethylation, and variable N-terminal acetylation, methionine oxidation, and phosphorylation of serine, threonine, and tyrosine; mass tolerances were 10 ppm and 0.02 Da with a 1% FDR. Differentially expressed phosphoproteins (log_2_ fold change [FC] >1.5 or <0.9) between control- and TRAILshort-treated CD3^+^, CD4^+^, or CD8^+^ cells were analyzed in RStudio (ggplot2, ComplexHeatmap, RColorBrewer) with KEGG enrichment and Benjamini-Hochberg correction (*P* < 0.05).

### TCR signaling study using Western blot analysis and flow cytometry

Serum-starved primary T cells were pretreated with TRAILshort peptide, scrambled control peptide, or BSA (3–4 hours) and then stimulated with CD3/CD28 Dynabeads (1:2 bead-to-cell ratio, 30 minutes). In separate experiments, cells were treated with EVs containing TRAILshort and/or full-length TRAIL, or TRAILshortΔTm at a 1:1,000 cell-to-EV ratio prior to CD3/CD28 activation. Cells were lysed in 50 mM Tris-HCl (pH 7.5), 150 mM NaCl, and 1% NP-40 with protease and phosphatase inhibitors, and then immunoblotted for expression of ZAP-70, p–ZAP-70 (Y319), LAT, p-LAT (Y191/Y220), PLCγ, p-PLCγ (Y783), p38, p-p38 (T180/Y182), and GAPDH. For flow cytometric analysis of p–ZAP-70, cells pretreated with TRAILshort or scrambled peptide (10–100 ng/mL) were fixed in 4% paraformaldehyde (PFA), permeabilized with ice-cold 90% methanol, and stained with anti–p–ZAP-70 (Y319; 1:1,000), followed by AF488-conjugated anti–rabbit IgG Fab2 antibody (1:1,000). Samples were acquired on a BD FACSCanto X and analyzed with FlowJo software (version 10.10.0).

### CD3ζ immunoprecipitation

Primary T cells were treated with TRAILshortΔTm or TRAILshort EVs (1:1,000) and CD3/CD28 beads (1:2), then lysed as above. Lysates were precleared with A/G agarose (20 μL, 4°C, 1 hour) and immunoprecipitated overnight with agarose-conjugated anti-CD3ζ antibody. Precipitates were washed 3 times, eluted with 0.1 M glycine-HCl (pH 2.6), resolved by SDS-PAGE, and immunoblotted for total ZAP-70, p–ZAP-70 (Y319), and CD3ζ.

### Assessment of T cell responses

#### ELISpot.

Ninety-six-well plates were coated with TRAILshort peptide (5 μg/mL) or BSA, and T cells were activated with CD3/CD28 Dynabeads (1:2, 24 hours) or treated with TRAILshort or TRAILshortΔTm EVs (1:1,000). Cells were then transferred to anti–IFN-γ–coated ELISpot plates (MABTECH) and exposed to recall antigens (tetanus toxoid, 5 μg/mL; HIV peptide mixture, 60 ng/mL) for 24 hours. Spots were detected with biotinylated anti–IFN-γ (MABTECH), avidin-HRP, and AEC substrate and quantified by ELISpot reader (CTL). For samples from patients with lymphoma, CD3^+^ T cells and CD19^+^ tumor B cells were isolated from splenocyte suspensions (STEMCELL Technologies), coincubated after T cell activation (24 hours), and assessed for IFN-γ secretion in the presence of anti-TRAILshort antibody or IgG1 isotype control.

#### Flow cytometry.

CD3^+^ T cells were pretreated with scrambled or TRAILshort peptide (1–100 ng/mL, 3–4 hours), stimulated with CD3/CD28 beads (1:2, 24 hours), and treated with brefeldin A for the final 4 hours. Intracellular IFN-γ was detected using a commercial staining kit (BioLegend, catalog 426803) and analyzed by flow cytometry.

#### IL-2 secretion.

To evaluate IL-2 secretion in response to TRAILshort treatment, a FluoroSpot assay was performed using CD3^+^ T cells cocultured with autologous macrophages. Prior to coculturing, monocytes isolated from PBMCs were differentiated into macrophages by treatment with 50 ng/mL macrophage-CSF (M-CSF) for 5 days. Macrophages were then loaded overnight with tetanus toxoid (5 μg/mL) and cocultured with autologous CD3^+^ T cells in the presence of either empty EVs or TRAILshort EVs (1:1,000 cell-to-EV ratio) for 24 hours. IL-2 secretion was subsequently quantified by FluoroSpot analysis (C.T.L.).

#### T cell activation.

CD3^+^ T cells were treated with scrambled or TRAILshort peptide (1–100 ng/mL), and CD69 expression was assessed by flow cytometry (anti–human CD69, BioLegend) at 6 hours. For antigen-specific responses, PBMCs were primed with EBV gp350 (1 μg/mL; Sino Biological, catalog 40373-V08H) for 5 days, rested for 24 hours in antigen-free media, and then cocultured with autologous gp350-loaded macrophages in the presence of TRAILshort or TRAILshortΔTm EVs (1:1,000). T cell activation (CD69^+^ and CD40L^+^) was assessed by flow cytometry at 6 hours, and IFN-γ secretion was quantified by ELISA (Abcam) at 24 hours.

#### Proliferation.

Primary T cells were pretreated for 3 hours with EVs, stimulated with CD3/CD28 beads (1:2, overnight), and seeded onto poly-l-ornithine–coated plates (20,000 cells/well); phase object confluence was measured on the Incucyte platform (Sartorius) and analyzed using GraphPad Prism 9. Where indicated, cells were pretreated with the SHP-1 inhibitor NSC-87877 (0.5 μM, Calbiochem) for 1 hour prior to TRAILshort EV treatment. In parallel, CFSE-labeled T cells (2.5 μM; Invitrogen, Thermo Fisher Scientific) were cocultured with WT or TRAILshort-expressing MEFs in a Transwell system and stimulated with CD3/CD28 beads (1:2); after 4 days, CFSE dilution in viable cells was assessed by flow cytometry and analyzed with FlowJo software.

### Generation of DR5-KO 293T cells

DR5-KO 293T cells were generated by CRISPR/Cas9 editing (Synthego) and confirmed by Western blotting (Supplemental Figure 1, A and C).

### NF-κB and NFAT reporter assay

Cells were transfected with NF-κB–Luc or NFAT-Luc (Addgene, catalog 111216 or 17870) reporter constructs plus *Renilla* luciferase (pRL-tk) using Lipofectamine LTX with Plus Reagent (Thermo Fisher Scientific), and then left untreated, treated with TRAILshort peptide, or stimulated with CD3/CD28 beads (1:2, positive control). After 24 hours, luciferase activity was measured using the Dual-Luciferase Reporter Assay System (Promega).

### Immunoprecipitation with Fc-fusion proteins and In-gel proteomics analysis

Primary CD3^+^ T cells (450 × 10^6^) were lysed in 50 mM Tris-HCl (pH 7.5), 150 mM NaCl, and 1% NP-40, divided into 3 aliquots, and precleared with A/G beads (50 μL, 4°C, 3 hours). Lysates were incubated overnight at 4°C with FAS-Fc, DR5-Fc (R&D Systems) or TRAILshort-Fc (ABClonal, custom), and then pulled down with A/G beads and eluted with glycine-HCl (pH 2.6) and neutralized with 1.5 M Tris-HCl. Immunoprecipitated proteins were resolved by SDS-PAGE and stained with Coomassie, and excised bands were submitted to the Mayo Clinic Proteomics Core for interactome analysis.

### SHP-1 immunoblotting and functional studies

Primary T cells were treated with TRAILshort peptide (100 ng/mL–5 μg/mL), TRAILshort EVs, full-length TRAIL EVs, or a combination (1:1,000 cell-to-EV ratio), and SHP-1 phosphorylation (Y536) was assessed by Western blotting. Jurkat cells were treated with TRAILshort peptide (5 μg/mL) or BSA (1 hour), incubated with anti-DR5 or IgG control antibody (2 μg, 30 minutes), and then lysed and immunoprecipitated with protein A/G beads; total and p–SHP-1 (Y564) levels were detected by Western blotting. To assess SHP-1 catalytic activity, DR5-KO 293T cells were cotransfected with DR5 and Flag-tagged WT or catalytically inactive (D419A) SHP-1 by electroporation, treated with TRAILshort peptide (1 μg/mL) or BSA for 3 hours, and immunoprecipitated with anti-Flag or anti-DR5 antibodies; precipitates were resolved by SDS-PAGE and probed for DR5 and SHP-1.

### Transfection of primary human T cells with the Accell PTPN6 siRNA pool

Purified CD3^+^ T cells (2 × 10^6^ cells/mL) were transfected with 1.0 μM Accell SMARTpool siRNA targeting PTPN6 or a nontargeting control siRNA (Dharmacon, catalog E-009778-00 or catalog D-001910-10) in the presence of 50 IU/mL IL-2 for 3–4 days (72). SHP-1 knockdown and cell viability were confirmed at 96 hours, after which cells were resuspended in serum-free RPMI for assessment of ZAP-70 phosphorylation as described above.

### CRISPR/Cas9 KO of PTPN6 in primary T cells

Chemically modified Alt-R CRISPR RNAs (crRNAs targeting PTPN6 or negative control crRNAs were duplexed with Alt-R tracrRNA (IDT, catalog 1072534) in Nuclease-Free Duplex Buffer (100 μM, 95°C, 5 minutes, then cooled to room temperature [RT]). Ribonucleoprotein complexes were assembled by combining 150 pmol of each crRNA-tracrRNA duplex (total 9 μL, 3 guides) with 180 pmol Cas9-NLS protein (UC Berkeley Macrolab) for 10 minutes at RT (73). Primary CD3^+^ T cells (1 × 10^7^) were resuspended in 100 μL P3 Nucleofector Solution (Lonza), mixed with RNP complexes, and nucleofected using the 4D-Nucleofector (program EO-115). Cells were immediately transferred to prewarmed medium with IL-2 (50 IU/mL), and functional assays were performed 3–5 days after nucleofection.

### Generation of TRAILshort-knockin mice and MEFs

F1 heterozygous TRAILshort-knockin mice were generated by Ingenious Targeting Laboratory via insertion of human TRAILshort cDNA with a LoxP-Neo-LoxP cassette into the Rosa26 locus under the CAG promoter. Targeted C57BL/6 ES cells were microinjected into BALB/c blastocysts; high-percentage chimeras were mated with C57BL/6N mice, and the F1 offspring were subsequently crossed with B6.C-Tg(CMV-cre)1Cgn/J mice (JAX: 006054, The Jackson Laboratory) to excise the Neo stop cassette and enable TRAILshort expression. MEFs were derived at E13.5 from Cre^+^TRAILshort^+^ intercrosses, yielding 25% WT, 25% homozygous, and 50% heterozygous TRAILshort embryos. Primary MEFs were cultured in DMEM (10% FBS, 1% penicillin-streptomycin, 1% glutamine) for 5 days, immortalized by retroviral transduction with SV40 large T antigen (74, 75), and then passaged at least 5 times. Where indicated, immortalized MEFs were transduced with a luciferase-GFP construct (Addgene, catalog 80389) and single-cell sorted to generate clonal lines (Supplemental Figure 5, B and C).

### MEF-derived EV isolation and apoptosis assays

WT or TRAILshort-expressing MEFs were cultured in DMEM with 2% EV-free FBS for 48 hours; conditioned media were cleared by sequential centrifugation (~200*g* for 5 min., followed by ~450*g* for 10 min.) and EVs pelleted by ultracentrifugation at 98,200*g* for 6 hours (Beckman Coulter, SW32 Ti rotor). For apoptosis assays, WT or homozygous TRAILshort MEFs were seeded overnight in 96-well plates, treated with increasing concentrations of mouse SuperKillerTRAIL (AdipoGen, catalog AG-40T-0004) or anti-Fas antibody (Antibodies Online, catalog ABIN1177304), and caspase 3/-7 activity was monitored every 2 hours for 40 hours using IncuCyte Caspase 3/-7 Dye (Sartorius, catalog 4440).

### Generation of CD19 CAR-T cells

CD19-directed CAR-T cells were generated as previously described (76–78). Briefly, T cells isolated from PBMCs by negative selection (STEMCELL Technologies) were stimulated with CD3/CD28 beads (3:1, 24 hours) and then transduced with lentiviral particles encoding a second-generation CAR19 construct (4-1BB/CD3ζ). T cells were expanded in the T cell medium consisting of X-Vivo 15 and human serum albumin for a total of 8 days. Beads were removed on day 6, and CAR-T cells were cryopreserved on day 8. Prior to use, cells were thawed and rested overnight in T cell medium.

### In vivo tumor models

#### TRAILshort MEF adoptive transfer in humanized CD34 mice.

Hu-CD34^+^ NSG mice were randomized by the percentage of human CD45^+^ engraftment into 2 groups (*n* = 6/group) and s.c. injected with 2 × 10^6^ luciferase-GFP–expressing WT or TRAILshort MEFs. Tumor progression was monitored weekly for 6 weeks by IVIS bioluminescence imaging and caliper measurements.

#### JeKo-1 xenograft and CAR-T therapy.

Female NSG mice (6–8 weeks old; JAX, catalog 005557, The Jackson Laboratory) were s.c. implanted bilaterally with 5 × 10^5^ WT or TRAILshort MEFs or PBS (*n* = 8/group). Three days later, 1 × 10^6^ luciferase-expressing JeKo-1 cells were administered i.v., and tumor engraftment was monitored by IVIS for 25 days. In the CAR-T experiment, WT or DR5-KO JeKo-1 cells were injected 3 days after MEF implantation; mice were grouped by tumor burden on day 6 and received 1.5 × 10^6^ CAR-T19 cells i.v. on day 7. Tumor progression was assessed weekly by IVIS for 32 days.

#### Hodgkin lymphoma model in humanized CD34 mice.

Hu-CD34^+^ NSG mice were randomized by human CD45^+^CD3^+^ engraftment (*n* = 6/group) and s.c. injected with 2 × 10^6^ WT or TRAIL-KO L428 cells. Tumor growth was monitored weekly by IVIS bioluminescence imaging.

### Histology

H&E staining, ISH, and IHC were performed on formalin-fixed, paraffin-embedded (FFPE) tissue sections. TRAILshort expression was detected using anti-TRAILshort antibody (2.2 mouse version) as previously described (27).

### Statistics

Data are presented as the mean ± SEM unless otherwise indicated. All tests were 2-tailed (α = 0.05). Two-group comparisons were performed using a paired or unpaired Student’s *t* test as appropriate, with a Wilcoxon signed-rank or rank-sum test used when parametric assumptions were not met. Analyses involving 3 or more conditions used 1-way ANOVA with post hoc testing for the same pairwise comparisons. A 1-sample *t* test was used for comparisons against a control normalized to 100. Bioluminescence signals were log transformed prior to analysis. Survival was analyzed using the log-rank (Mantel-Cox) test. The AUC compared GFP uptake over time between conditions. Where multiple comparisons were made, *P* values were adjusted to control the family-wise error rate, with the specific method stated in each figure legend. Data were analyzed and visualized using R, version 4.5.2 (rstatix, tidyverse), and GraphPad Prism 9.

### Study approval

All studies involving humans were reviewed and approved by the Mayo Clinic IRB (Rochester, Minnesota, USA). Apheresis cones were obtained from uninfected donors (IRB no. 1039-03) and HIV^+^ donors (IRB no. 13-005646). Bulk splenocytes were obtained from splenectomy specimens from individuals with hematologic malignancies (IRB nos. 118-01 and 1827-00). Written informed consent was obtained from all individuals prior to participation, in accordance with IRB-approved protocols. All animal studies were reviewed and approved by the Mayo Clinic IACUC (protocol no. A00001767-16-R25) and were conducted in accordance with institutional and national guidelines for the care and use of laboratory animals.

### Data availability

The mass spectrometry proteomics data are deposited in the ProteomeXchange Consortium via the PRIDE partner repository (dataset identifier PXD078420).

## Author contributions

SJ, SN, ZN, YZ, IC, BMH, TVZ, AK, MAM, APC, RB, and DGM conducted the experiments. CC, EGR, RM, DDM, and HL were involved in data analysis. AB supplied EV for the study. ADB designed the experiments and supervised the implementation of the work. SJ and ADB wrote the manuscript. APC reviewed histology results and edited the manuscript. SMA provided lymphoma tumor samples. SKK designed the CAR-T cell therapy. TT, CMW, DDB, and AP made conceptual contributions to the manuscript. SJ, SN, and ZN share the first-author position. Author order among these co–first authors was determined by relative contribution to the study, with all three performing critical experiments and SJ listed first because SJ led manuscript drafting and preparation.

## Conflict of interest

This research has been reviewed by the Mayo Clinic Conflict of Interest Review Board and is being conducted in compliance with Mayo Clinic conflict-of-interest policies. ADB, SN, AP, DGM, SJ, IC, SSK, and Mayo Clinic have a financial interest related to this research. ADB is a paid consultant for Abbvie, Gilead, MarPam, Rion, and Symbiosis; owns equity in and conducts scientific advisory work for Zentalis, Rion, Tornado Biosciences, Sendero, and nference. ADB is on the board of directors of Paradox Immunotherapeutics, Sendero, and BioPhyle.

## Funding support

This work is the result of NIH funding, in whole or in part, and is subject to the NIH Public Access Policy. Through acceptance of this federal funding, the NIH has been given a right to make the work publicly available in PubMed Central.

- National Institute of Allergy and Infectious Diseases (NIAID), NIH (AI110173, AI83801, and 1UM1AI164562, to ADB).
- Mayo Clinic HH Sheikh Khalifa Bin Zayed Al Nahyan Professorship of Infectious Diseases (to ADB).
- Skoll Foundation, through the Mayo–Skoll Extracellular Vesicles Collaboration to Advance Novel Therapeutic Approaches for Autoimmune Disease (to ADB).
- National Cancer Institute (NCI), NIH (U01CA271410 and P30CA15083 to AP).

## Supplementary Material

Supplemental data

Unedited blot and gel images

Supplemental table 1

Supplemental table 2

Supplemental table 3

Supplemental table 4

Supporting data values

## Version 1 08/03/2026

Electronic publication

## Version 2 08/12/2026

corrected typos in the funding support section
