# Supplementary material for: TRAIL splice variant TRAILshort disrupts T cell receptor signaling and promotes immune tolerance in vivo
Source: J Clin Invest. 2026 Aug 3;136(15):e194449. doi: 10.1172/JCI194449 (PMC13430026; doi:10.1172/JCI194449)
Supplement: Supplemental data [file jci-136-194449-s051.pdf]

Supplemental Materials

Supplementary Figure Legends

Suppl. Fig. 1

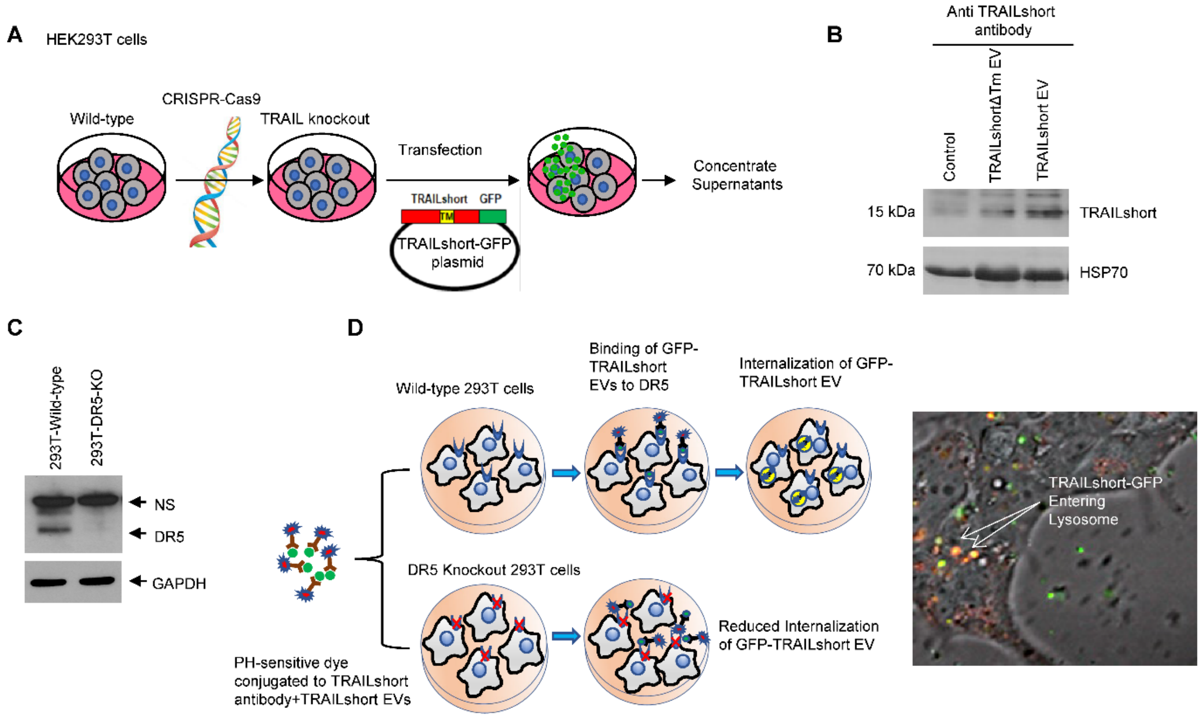

Supplementary Fig. 1. (A) Schematic representation of the generation of GFP-tagged TRAILshort EVs in TRAIL knockout 293T cells. **(B)** Western blot analysis of the isolated EVs confirms the expression of TRAILshort and TRAILshortΔTm, as detected using an anti-TRAILshort antibody, while HSP70 serves as an EV-specific marker. **(C)** Western blotting shows the absence of DR5 expression in 293T DR5 knockout cells. **(D)** Schematic diagram of the experimental setup showing the treatment of wild-type and DR5 knockout 293T cells with GFP-TRAILshort containing EVs pre-labeled with pH-sensitive dye-conjugated antibodies to track EV internalization (left). The representative confocal fluorescence image (right) shows GFP-TRAILshort EV uptake in wild-type 293T cells, visualized as yellow puncta, indicating DR5-dependent EV internalization.

Suppl. Fig. 2

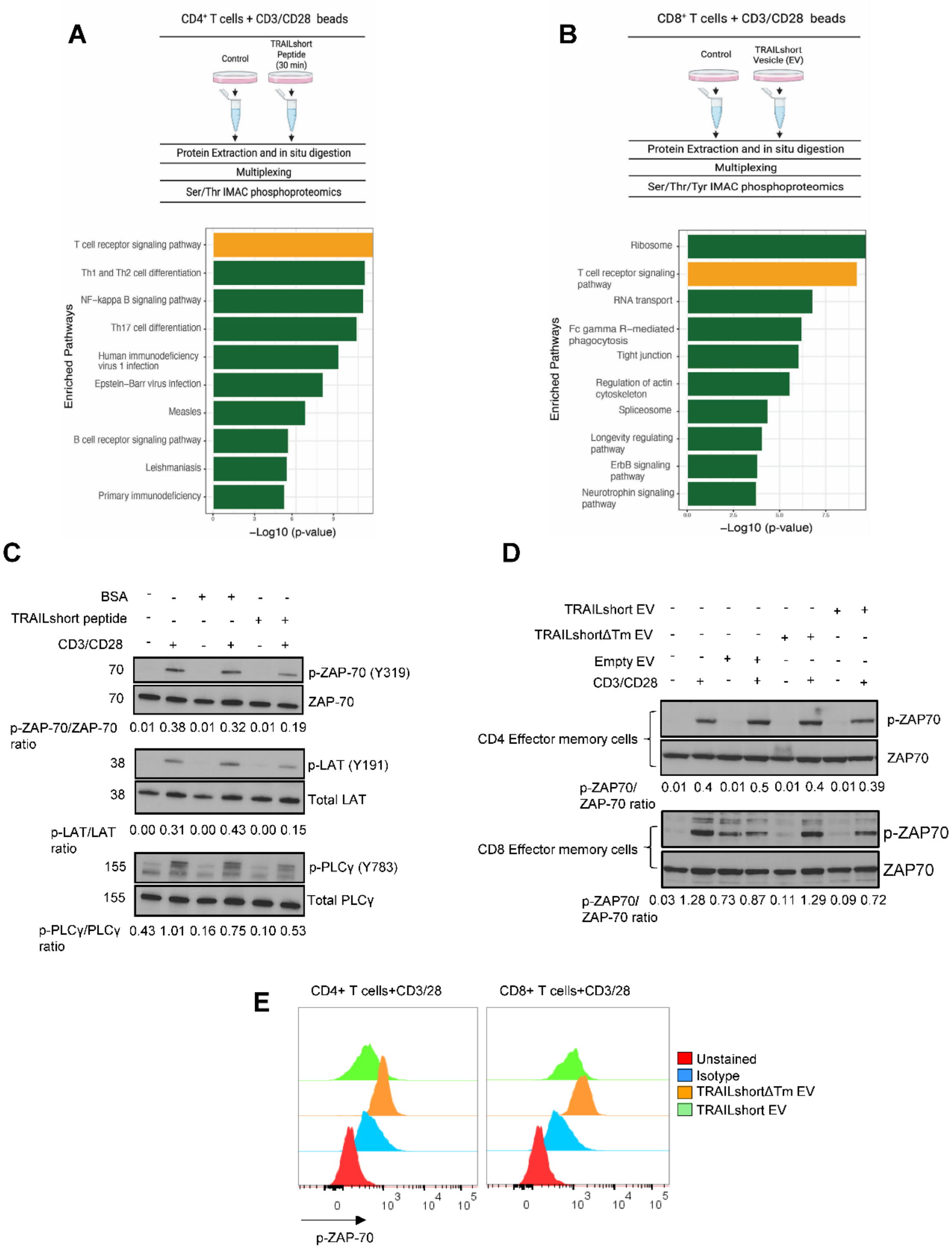

Supplementary Fig. 2.

(A & B) Experimental conditions for CD4$^+$ and CD8$^+$ T cells treated with TRAILshort in peptide or EV form prior to phospho-proteomic analysis. Bar graphs show differentially expressed phospho-

proteins relative to untreated controls. KEGG pathway enrichment was performed on phosphoproteins with log_2 fold change (log_2FC) > 1.5 or < 0.9, identifying significant modulation of TCR signaling pathways under both treatment conditions. **(C & D)** Immunoblot analysis of primary $CD3^+$ T cells pretreated with BSA or TRAILshort peptide (5 μg/mL) **(C)**, or with TRAILshortΔTm or TRAILshort EVs (1000:1 EV:cell ratio) **(D)** for 2-3 hours, followed by stimulation with CD3/CD28 beads (1:2 bead:cell ratio) for 30 min. Blots were probed for total and phosphorylated ZAP-70, LAT, or PLCγ. Values indicate the ratio of phosphorylated to total protein. (**E)** Histograms showing flow cytometry analysis of p-ZAP-70 in CD4+ and CD8+ T cells treated with TRAILshort or TRAILshortΔTm EVs and stimulated with CD3/CD28 beads, as indicated above.

Suppl. Fig. 3

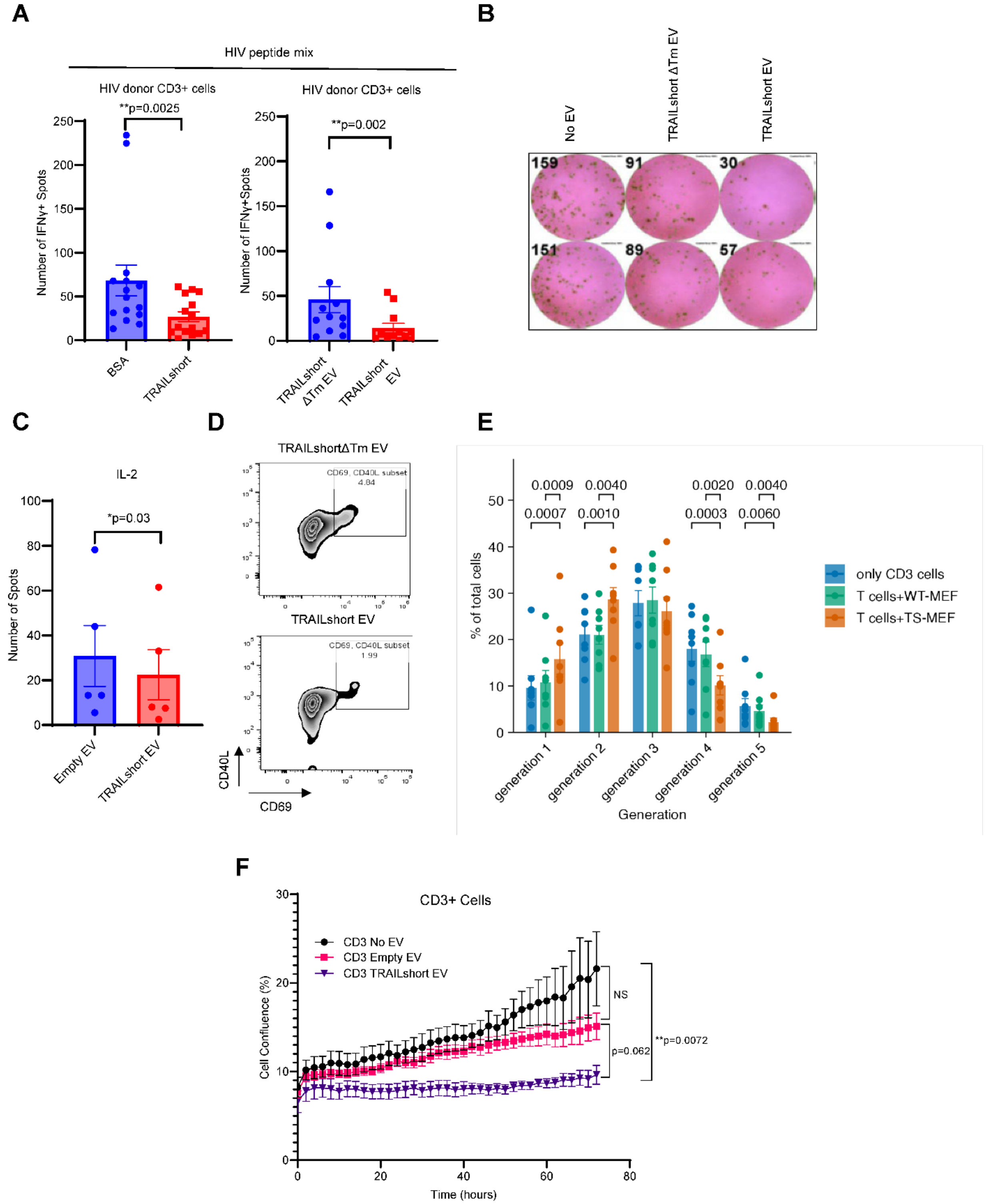
A
HIV peptide mix
HIV donor CD3+ cells
**p=0.0025
Number of IFNγ+ Spots
0
50
100
150
200
250
BSA
TRAILshort
HIV donor CD3+ cells
**p=0.002
Number of IFNγ+Spots
TRAILshort ΔTm EV
TRAILshort EV
B
No EV
TRAILshort ΔTm EV
TRAILshort EV
159
91
30
151
89
57
C
IL-2
*p=0.03
Number of Spots
0
20
40
60
80
100
Empty EV
TRAILshort EV
D
TRAILshortΔTm EV
CD69, CD40L subset
4.84
TRAILshort EV
CD69, CD40L subset
1.99
CD40L
CD69
E
0.0009
0.0040
0.0020
0.0040
0.0007
0.0010
0.0003
0.0060
% of total cells
0
10
20
30
40
50
generation 1
generation 2
generation 3
generation 4
generation 5
Generation
only CD3 cells
T cells+WT-MEF
T cells+TS-MEF
F
CD3+ Cells
CD3 No EV
CD3 Empty EV
CD3 TRAILshort EV
Cell Confluence (%)
0
10
20
30
NS
p=0.062
**p=0.0072
0
20
40
60
80
Time (hours)

Suppl. Fig. 3

G

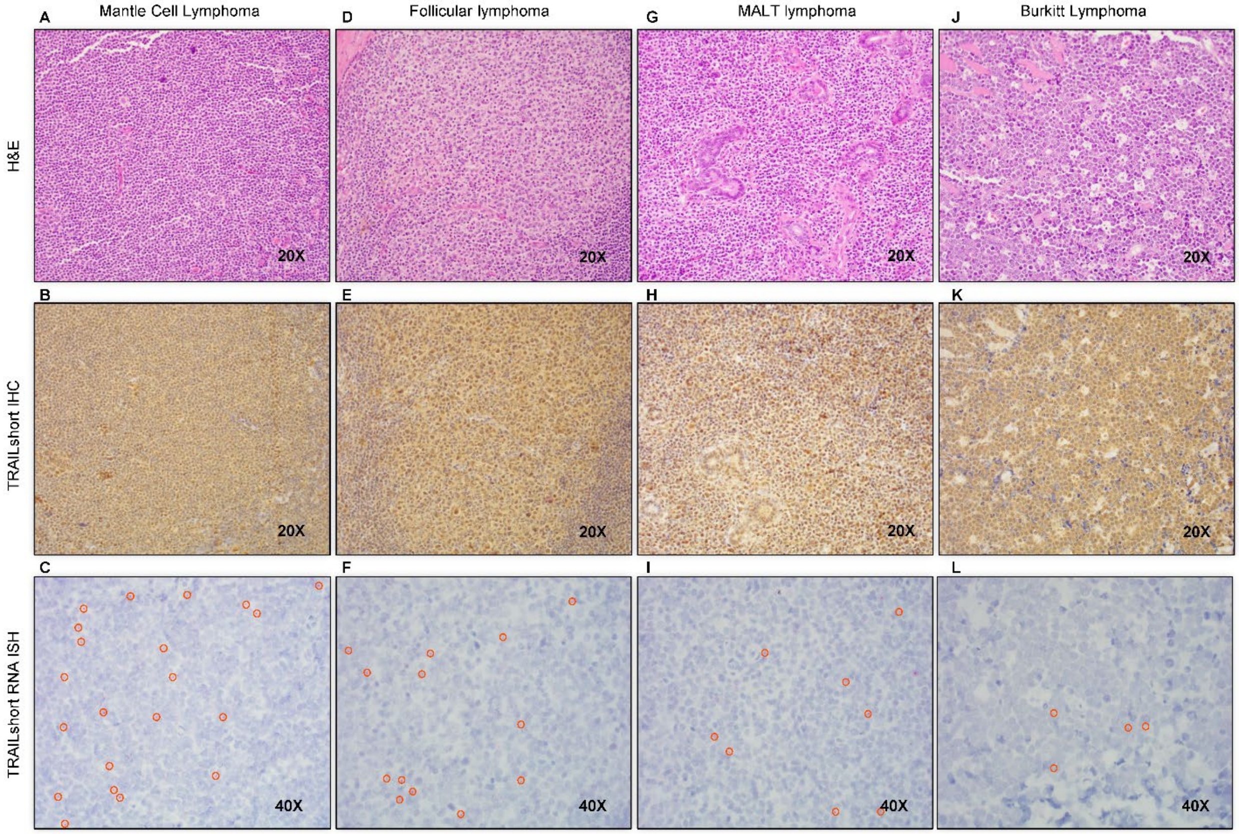

H

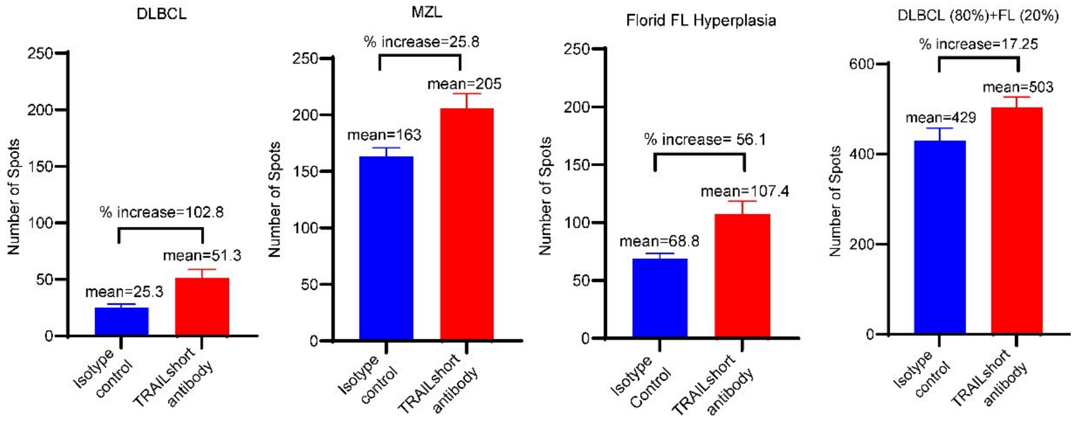

Supplementary Fig. 3.

(A) Bar graph showing ELISpot analysis of IFNγ spot formation in T cells treated with BSA or TRAILshort peptide (5 μg/mL) or exposed to TRAILshortΔTm or TRAILshort EVs (1:1000 cell-to-EV ratio), followed by stimulation with HIV pepmix. Statistical analysis using the Wilcoxon matched-pairs signed-rank test shows a significant difference between TRAILshort-treated samples (EV or peptide) and their corresponding control-treated samples ($p<0.05$). **(B)** Representative ELISpot images obtained using a CTL reader demonstrating reduced IFNγ spot formation following TRAILshort EV treatment. **(C)** Quantification of IL-2 secretion from T cells treated with TRAILshort EVs and co-cultured with syngeneic macrophages loaded with tetanus antigen, as measured using a Human IL-2 FluoroSpot assay kit. TRAILshort EV treatment significantly reduces IL-2 spot formation compared with control conditions (paired t-test; $p < 0.05$). **(D)** Flow cytometry analysis showing a reduced frequency of activated T cells ($CD40L^+/CD69^+$) in response to TRAILshort EV exposure. **(E)** Bar graphs showing statistical analysis (paired t-test; $p < 0.05$) of T-cell proliferation assessed by CFSE dilution following treatment with TRAILshort.

(F) $CD3^+$ T cells were treated with no EVs, empty vector EVs, or TRAILshort EVs and subsequently stimulated with CD3/CD28 beads overnight. Cell proliferation was monitored for 72 hours using IncuCyte live-cell imaging. Cell growth was quantified by calculating the area under the confluence-versus-time curve (AUC). Mean AUC values from technical replicate wells were compared using ordinary one-way ANOVA followed by Tukey's multiple-comparisons test (**$p<0.01$). **(G)** Lymphoid tissues from patients with the indicated B-cell malignancies exhibit TRAILshort positivity by immunohistochemistry (IHC) and *in situ* hybridization (ISH): (a–c) Mantle Cell Lymphoma, (d–f) Follicular Lymphoma, (g–i) MALT Lymphoma, and (j–l) Burkitt Lymphoma.

(H) Bar graphs summarizing IFNγ ELISpot data from T cells isolated from spleens of lymphoma patients. T cells were stimulated with CD3/CD28 beads and co-cultured with autologous B-cell tumors in the presence of either anti-TRAILshort antibody or matched isotype control. Patient cohorts included Chronic Lymphocytic Leukemia (CLL), Follicular Lymphoma (FL), and Diffuse Large B-cell Lymphoma (DLBCL). TRAILshort antibody increases the number of IFN-γ spots.

Suppl. Fig. 4

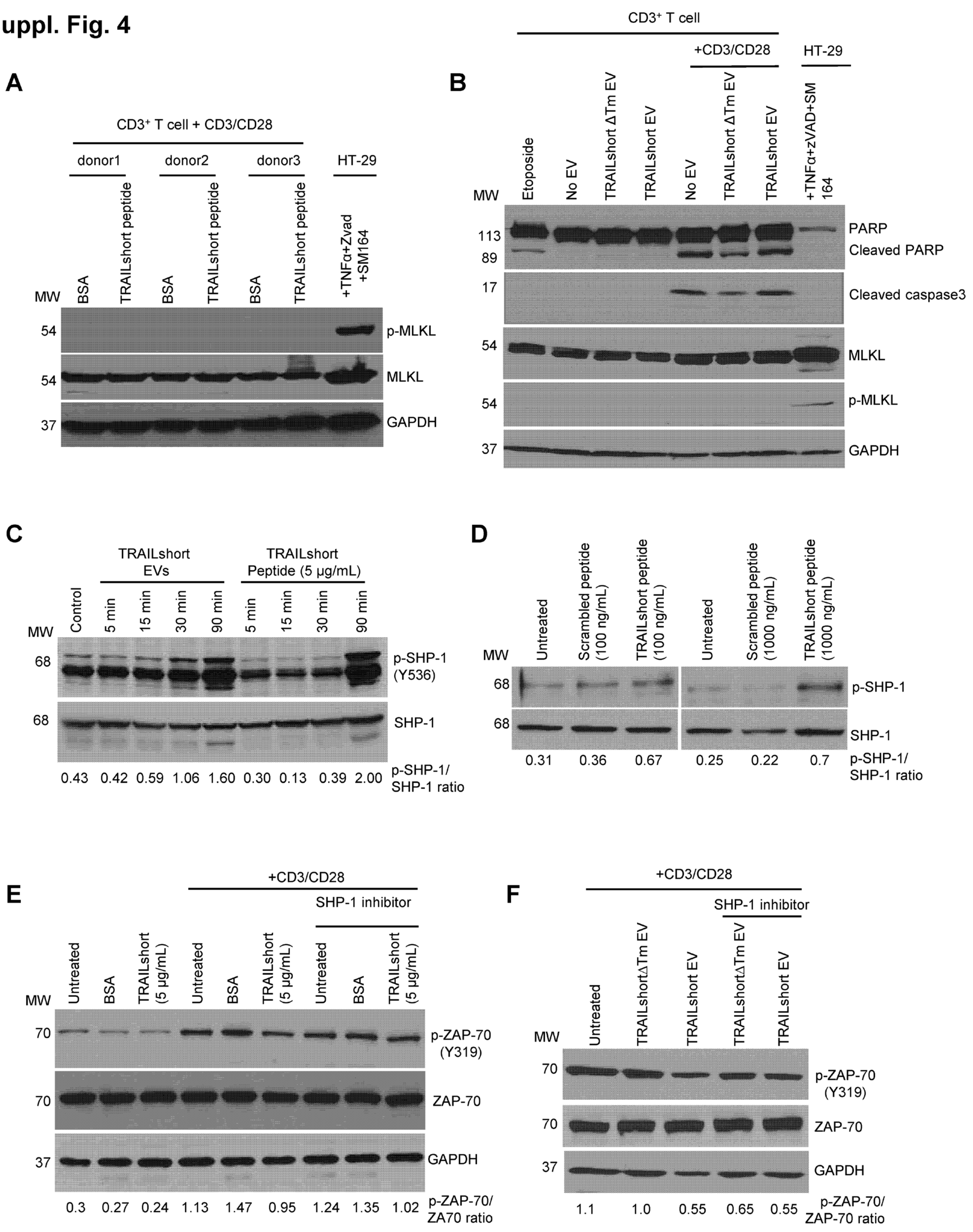
A
CD3+ T cell + CD3/CD28
donor1
donor2
donor3
HT-29
MW
BSA
TRAILshort peptide
BSA
TRAILshort peptide
BSA
TRAILshort peptide
+TNFα+Zvad +SM164
54
54
37
p-MLKL
MLKL
GAPDH
B
CD3+ T cell
+CD3/CD28
HT-29
MW
Etoposide
No EV
TRAILshort ΔTm EV
TRAILshort EV
No EV
TRAILshort ΔTm EV
TRAILshort EV
+TNFα+zVAD+SM 164
113
89
17
54
54
37
PARP
Cleaved PARP
Cleaved caspase3
MLKL
p-MLKL
GAPDH
C
TRAILshort EVs
TRAILshort Peptide (5 µg/mL)
MW
Control
5 min
15 min
30 min
90 min
5 min
15 min
30 min
90 min
68
68
p-SHP-1 (Y536)
SHP-1
0.43 0.42 0.59 1.06 1.60 0.30 0.13 0.39 2.00
p-SHP-1/ SHP-1 ratio
D
MW
Untreated
Scrambled peptide (100 ng/mL)
TRAILshort peptide (100 ng/mL)
Untreated
Scrambled peptide (1000 ng/mL)
TRAILshort peptide (1000 ng/mL)
68
68
p-SHP-1
SHP-1
0.31 0.36 0.67 0.25 0.22 0.7
p-SHP-1/ SHP-1 ratio
E
+CD3/CD28
SHP-1 inhibitor
MW
Untreated
BSA
TRAILshort (5 µg/mL)
Untreated
BSA
TRAILshort (5 µg/mL)
Untreated
BSA
TRAILshort (5 µg/mL)
70
70
37
p-ZAP-70 (Y319)
ZAP-70
GAPDH
0.3 0.27 0.24 1.13 1.47 0.95 1.24 1.35 1.02
p-ZAP-70/ ZA70 ratio
F
+CD3/CD28
SHP-1 inhibitor
MW
Untreated
TRAILshortΔTm EV
TRAILshort EV
TRAILshortΔTm EV
TRAILshort EV
70
70
37
p-ZAP-70 (Y319)
ZAP-70
GAPDH
1.1 1.0 0.55 0.65 0.55
p-ZAP-70/ ZAP-70 ratio

Suppl. Fig. 4G

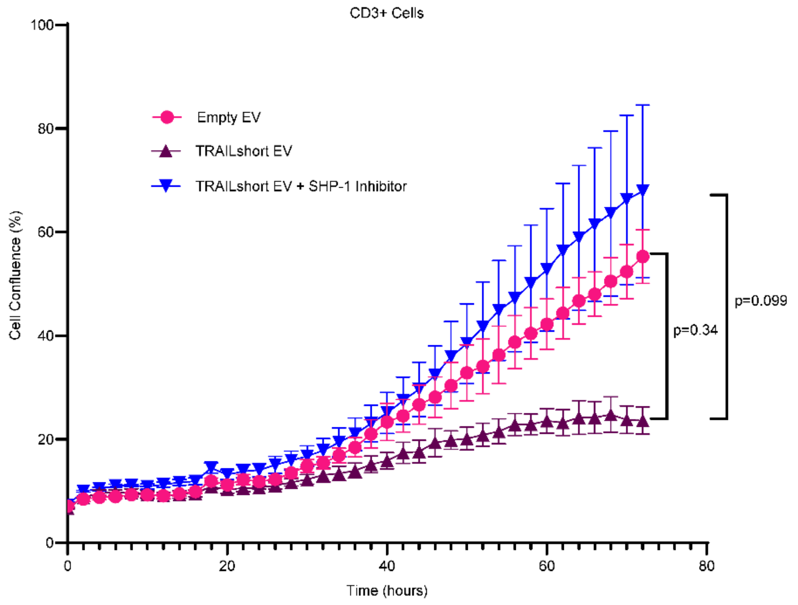

Supplementary Fig. 4.

(A) Primary CD3⁺ T cells were treated with TRAILshort (5 μg/mL) or BSA control and stimulated with CD3/CD28 beads (1:2 bead-to-cell ratio). After 48 h, cell lysates were analyzed by immunoblotting for phosphorylated MLKL (p-MLKL) and total MLKL to assess necroptosis. **(B)** CD3⁺ T cells were treated with TRAILshortΔTm or TRAILshort-containing EVs at a 1:1000 cell-to-EV ratio. Lysates were immunoblotted for cleaved caspase-3 and cleaved PARP (apoptosis markers), as well as p-MLKL and total MLKL (necroptosis markers). GAPDH served as a loading control. HT-29 cells treated with TNFα (20 ng/mL), zVAD (20 μM), and SM164 (100 nM), and CD3⁺ T cells treated with etoposide (10 μM), were used as positive controls. MLKL, mixed lineage kinase domain–like protein; PARP, poly (ADP-ribose) polymerase. **(C)** Primary CD3⁺ T cells were treated with TRAILshort-containing EVs or TRAILshort peptide (5 μg/mL) for the indicated times, followed by immunoblot analysis of total and phosphorylated SHP-1. **(D)** Cells were treated with scrambled or TRAILshort peptide (100 or 1000 ng/mL) for 30 min and analyzed by immunoblotting for total and phosphorylated SHP-1. **(E, F)** Primary CD3⁺ T cells were pretreated with 1 μM SHP-1 inhibitor (NSC-87877) for 1 h, then exposed to either TRAILshortΔTm or TRAILshort EVs **(E)**, or to BSA or TRAILshort peptide (5 μg/mL) for 1 h **(F)**, prior to stimulation with CD3/CD28 beads (1:1 bead-to-cell ratio). Lysates were immunoblotted for total and phosphorylated ZAP70, with GAPDH as a loading control. **(G)** CD3+ T cells were treated with TRAILshort EV or TRAILshort

EV plus an SHP-1 inhibitor (0.5 μM), followed by overnight stimulation with CD3/CD28 beads. T cell proliferation was monitored using IncuCyte Live-Cell analysis over 72 hours. Mean AUC values from technical replicate wells were compared using ordinary one-way ANOVA followed by Tukey's multiple-comparisons test.

Suppl. Fig. 5

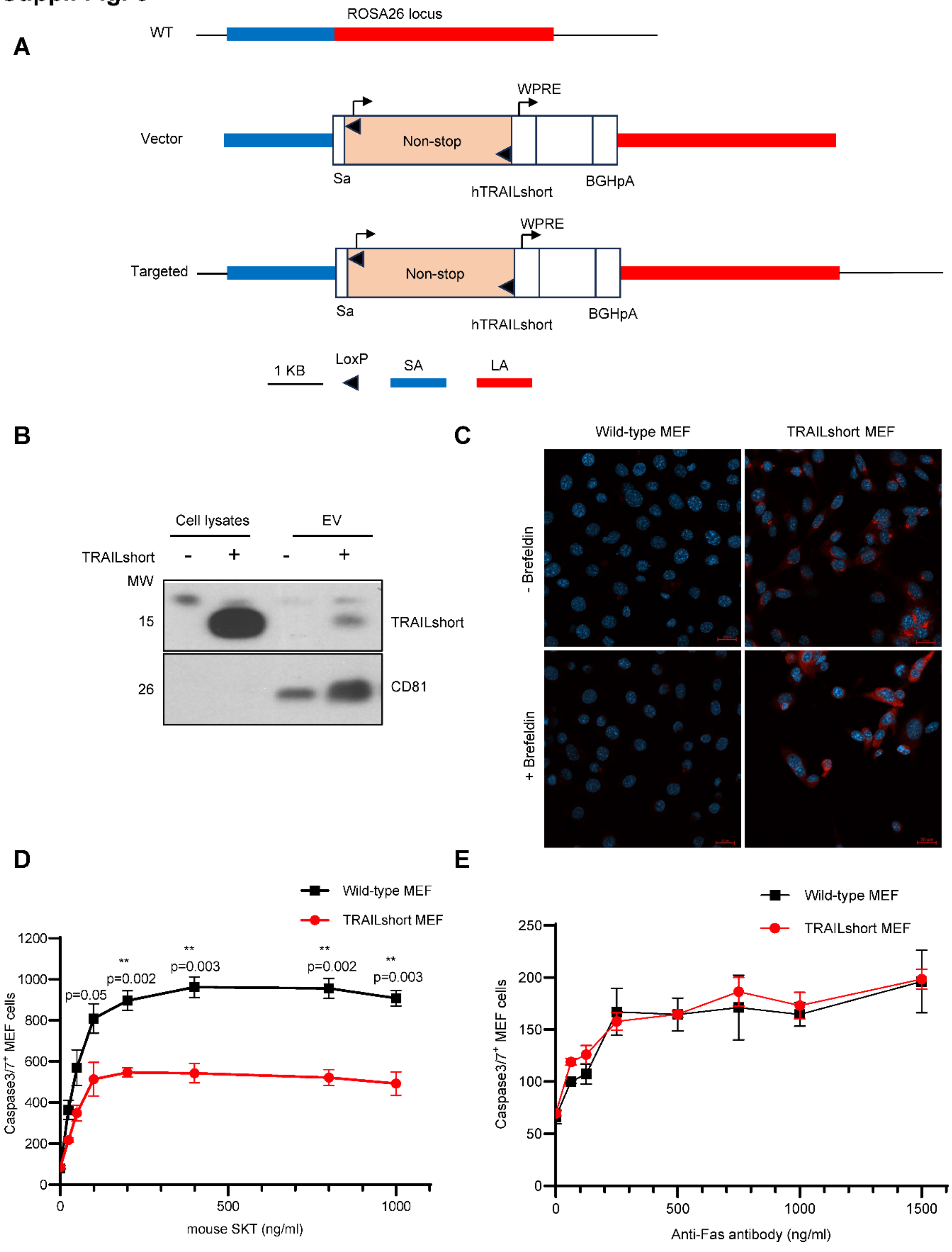

Supplementary Fig. 5.

(A) Schematic representation of the strategy used to generate ROSA26 transgenic human TRAILshort knock-in mice. **(B)** Western blot analysis showing that TRAILshort is expressed in mouse embryonic fibroblasts (MEFs) isolated from TRAILshort knock-in mice and is also secreted in the EVs released by these MEFs. Blots were probed using an anti-TRAILshort antibody (mouse version; 2.2). CD81 is an EV marker. **(C)** Immunofluorescence staining reveals TRAILshort protein expression in TRAILshort MEF cells, but not in wild-type MEF cells. Brefeldin A treatment leads to the accumulation of TRAILshort in the cytosol of TRAILshort MEF cells. **(D & E)** MEFs expressing TRAILshort are resistant to apoptosis induced by mouse super-killer TRAIL (mouse-SKT) but remain susceptible to Fas-mediated cell apoptosis. Wild-type and TRAILshort MEFs were cultured with increasing doses of mouse SKT **(D)** or an anti-Fas antibody **(E)**, and apoptosis was assessed by quantifying positive cells for active caspase-3/7 staining using IncuCyte over a 40-hour period. The graphs show IncuCyte data collected at the 30-hour time point post-treatment. Two-sample t-test shows significant differences between wild-type and TRAILshort MEFs at the indicated concentrations (**p <0.01).

Suppl. Fig. 6

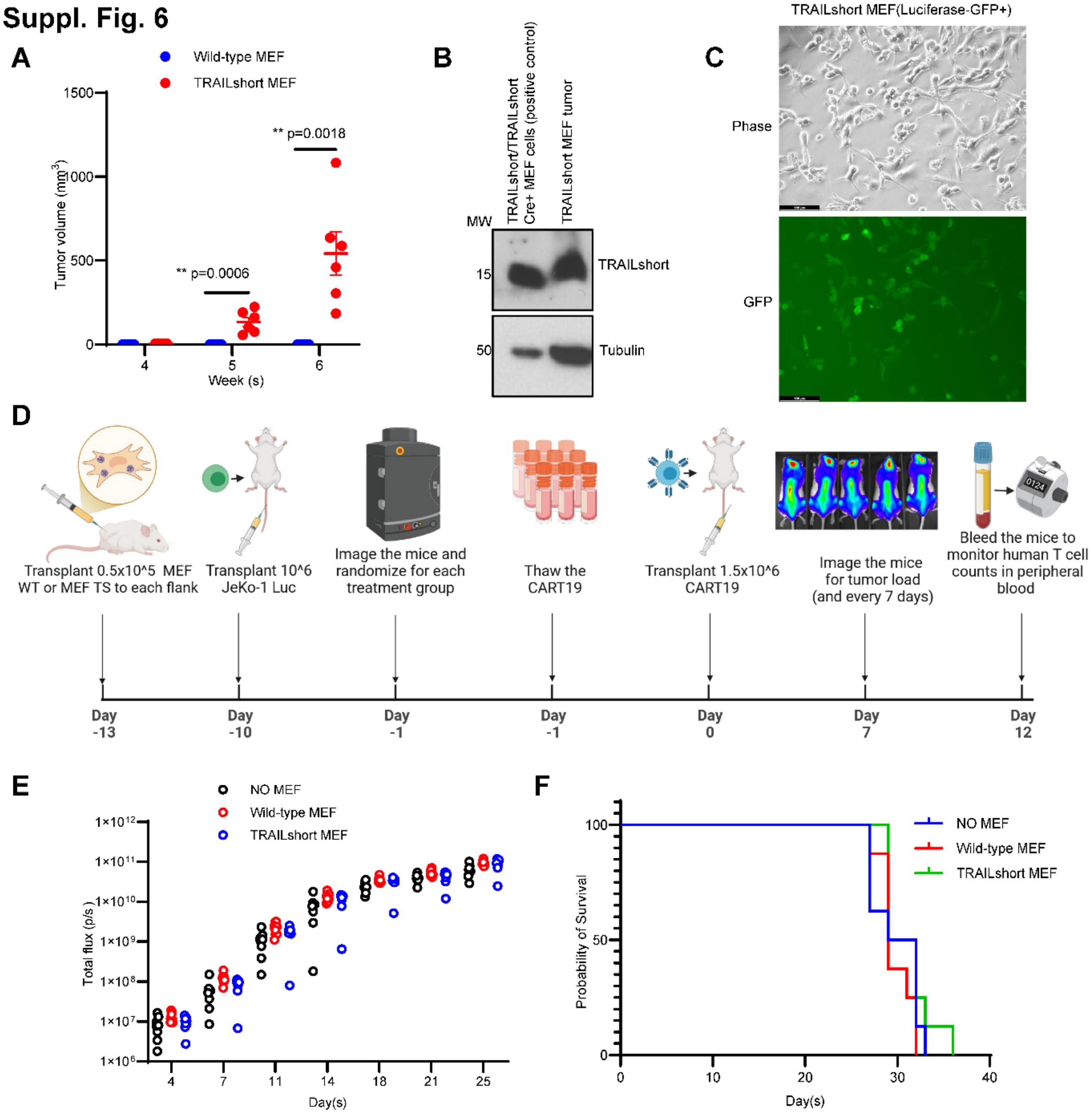

Supplementary Fig. 6.

(A) Graphs showing tumor volume of palpable tumors detected 4 weeks after wild-type or TRAILshort MEF cell transplantation in CD34+ humanized mice. Multiple t-tests with Bonferroni correction were used to compare tumor volume between two animal groups. **p<0.01 denotes significant difference between groups. **(B)** Tumor tissues were dissected from mice, minced, lysed and run on SDS-PAGE gel. Western Blot shows the expression of TRAILshort in the tumors derived from TRAILshort MEFs in CD34+ humanized mice. **(C)** Cell suspensions isolated from dissected TRAILshort MEF tumors were cultured and analyzed for GFP expression using fluorescent microscope (400X magnification, Scale bar=100 μm). **(D)** Diagram illustrating the

detailed procedure for implanting MEF (wild-type and TRAILshort MEF) and JeKo-1 cells, followed by the introduction of CAR-T19 cells into NSG mice. **(E)** Mice were subcutaneously implanted in both flanks with 5 × 10^5 wild-type MEFs (n = 8), TRAILshort-expressing MEFs lacking luciferase constructs (n = 8), or PBS (no MEF control; n = 8). Three days later, 1 × 10^6 luciferase-labeled JeKo-1 cells were injected intravenously. Tumor engraftment and progression were tracked for 25 days by bioluminescence imaging (IVIS). No significant differences in tumor growth were observed among groups. **(F)** Kaplan–Meier survival analysis showing no significant differences in survival across mice corresponding to the experimental groups described in **(E).**

Suppl. Fig. 7

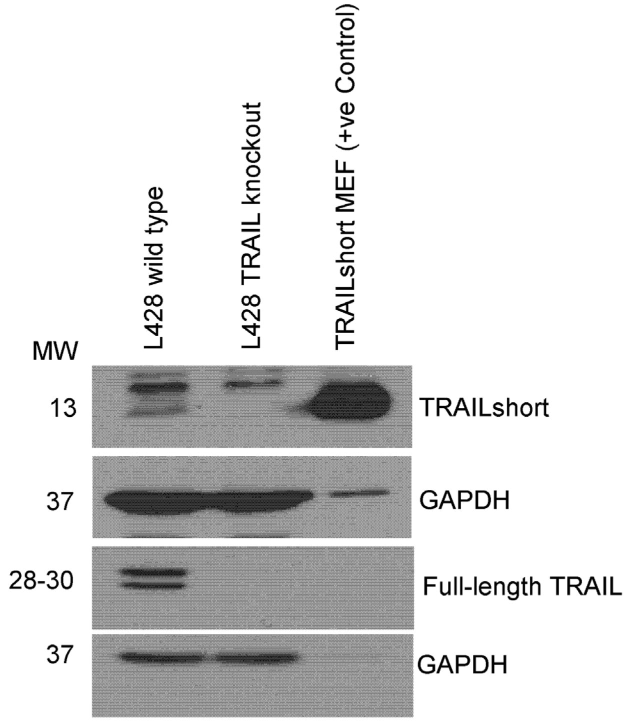

Supplementary Fig. 7.

Western blot analysis shows the levels of full-length TRAIL and TRAILshort protein expression, in both wild-type and TRAIL knockout L428 cells, with GAPDH used as a loading control. TRAILshort expressing MEF cells are used as positive control.
