# Supplementary material for: TRAIL splice variant TRAILshort disrupts T cell receptor signaling and promotes immune tolerance in vivo
Source: J Clin Invest. 2026 Aug 3;136(15):e194449. doi: 10.1172/JCI194449 (PMC13430026; doi:10.1172/JCI194449)

Western Blot Original/Unmodified images used in the manuscript

Manuscript title: TRAILshort disrupts T cell receptor signaling and promotes immune tolerance *in vivo*.

Fig. 2B

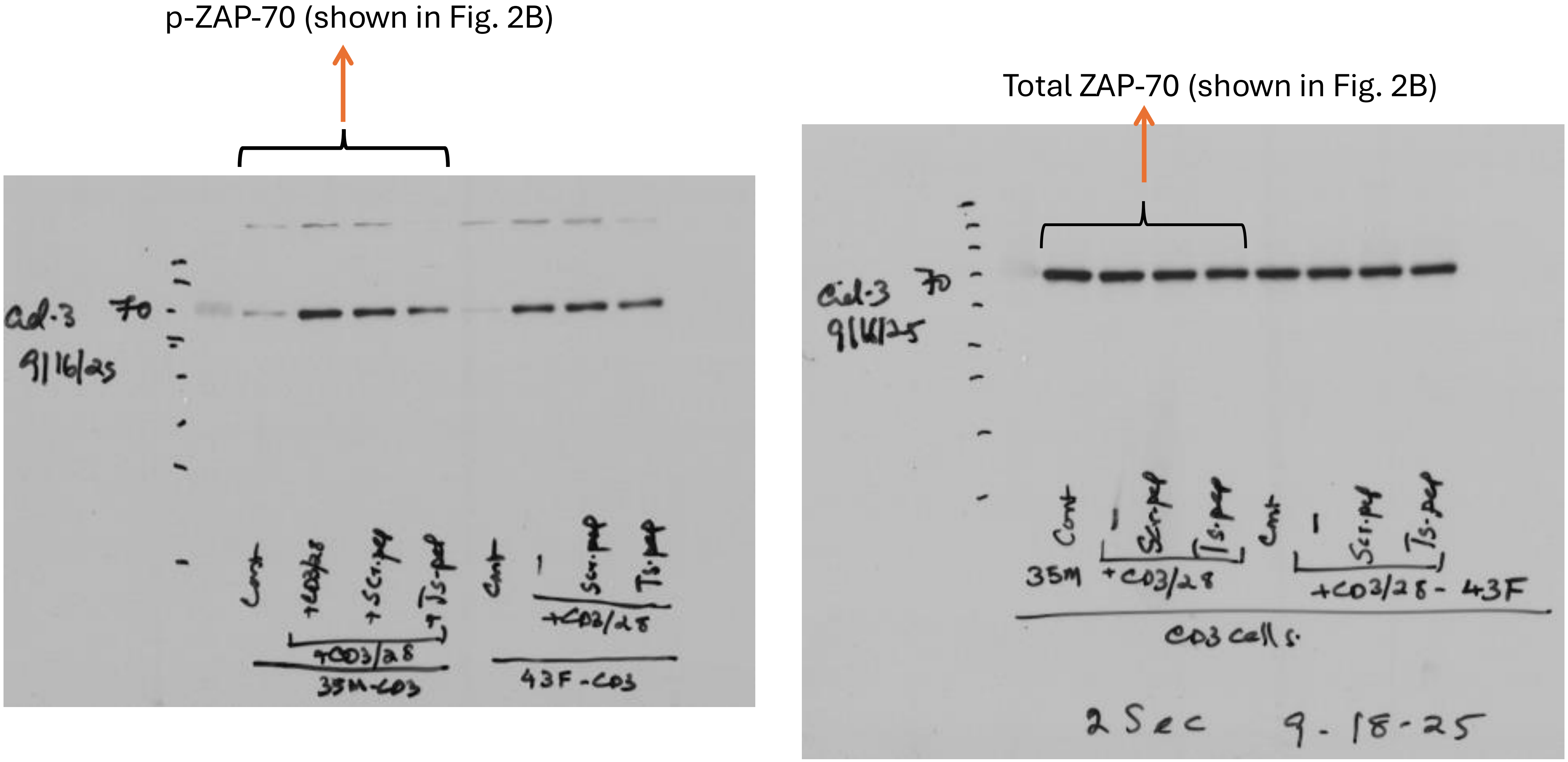

CD3 cells

Fig. 2F

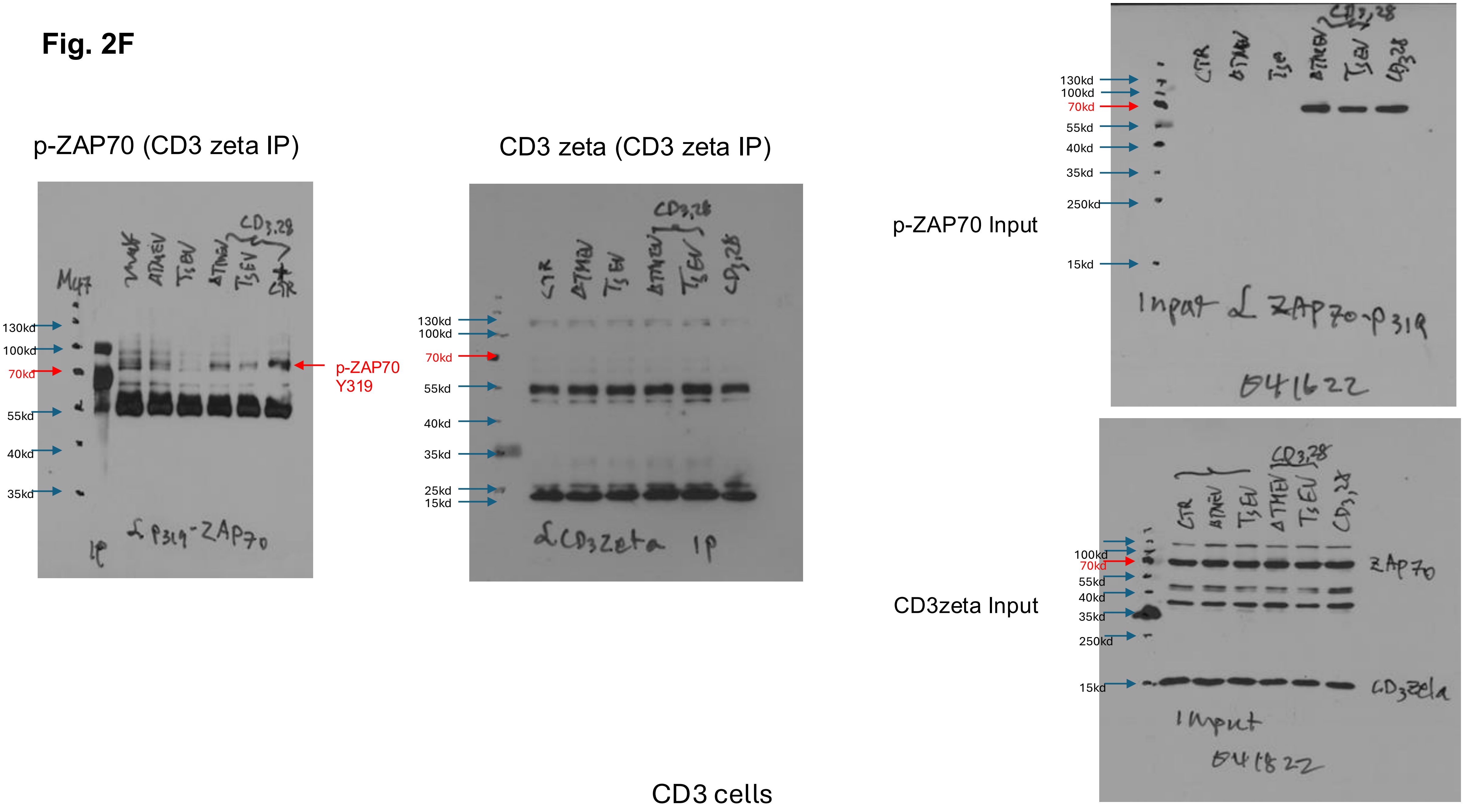

p-ZAP70 (CD3 zeta IP)
130kd
100kd
70kd
55kd
40kd
35kd
p-ZAP70 Y319
CD3 zeta (CD3 zeta IP)
130kd
100kd
70kd
55kd
40kd
35kd
25kd
15kd
p-ZAP70 Input
130kd
100kd
70kd
55kd
40kd
35kd
250kd
15kd
CD3zeta Input
100kd
70kd
55kd
40kd
35kd
250kd
15kd
CD3 cells

Fig. 2G

p-ZAP-70 (shown in Fig. 2G)

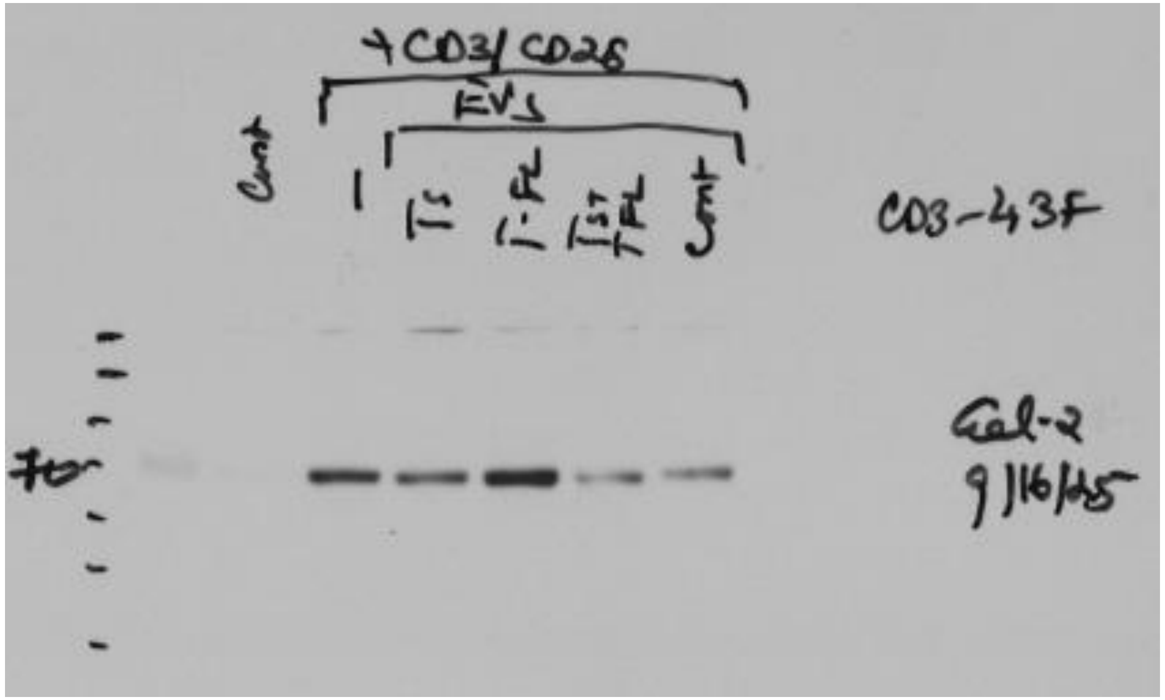

Total ZAP-70 (shown in Fig. 2G)

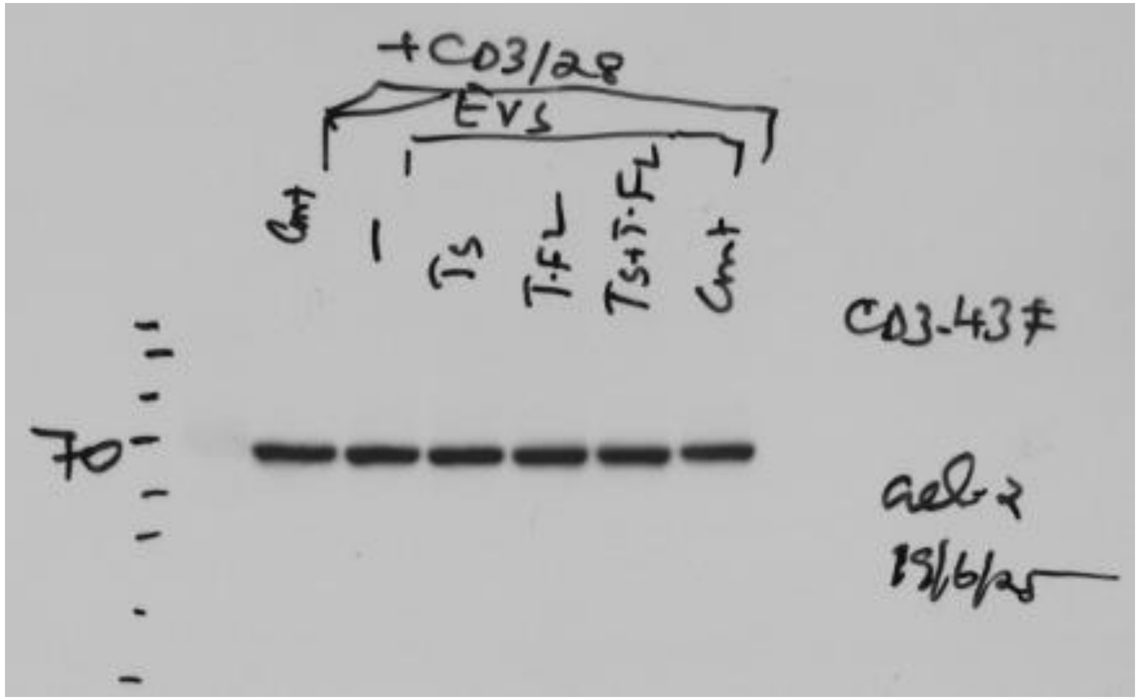

CD3 cells

Fig. 4A

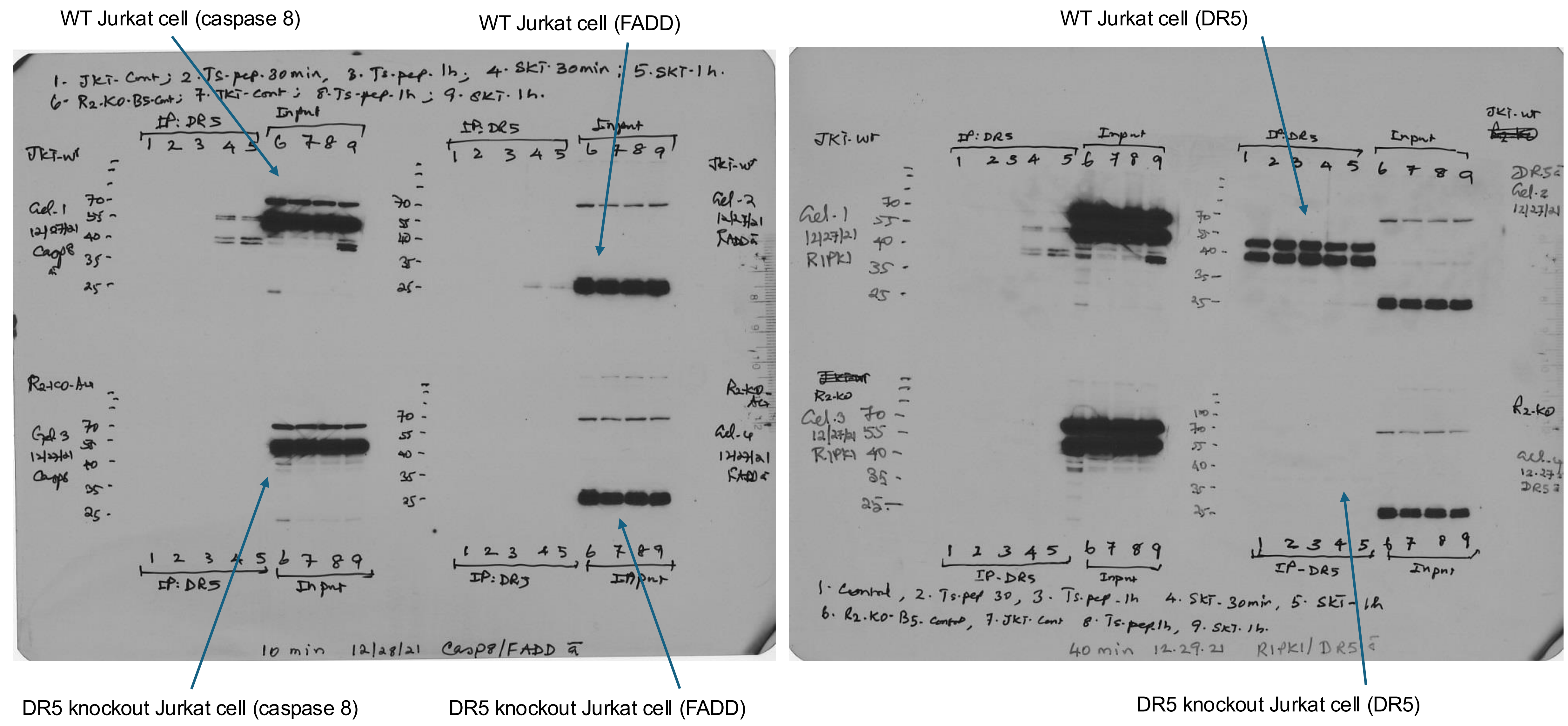
WT Jurkat cell (caspase 8)
WT Jurkat cell (FADD)
WT Jurkat cell (DR5)
DR5 knockout Jurkat cell (caspase 8)
DR5 knockout Jurkat cell (FADD)
DR5 knockout Jurkat cell (DR5)

Fig. 4B

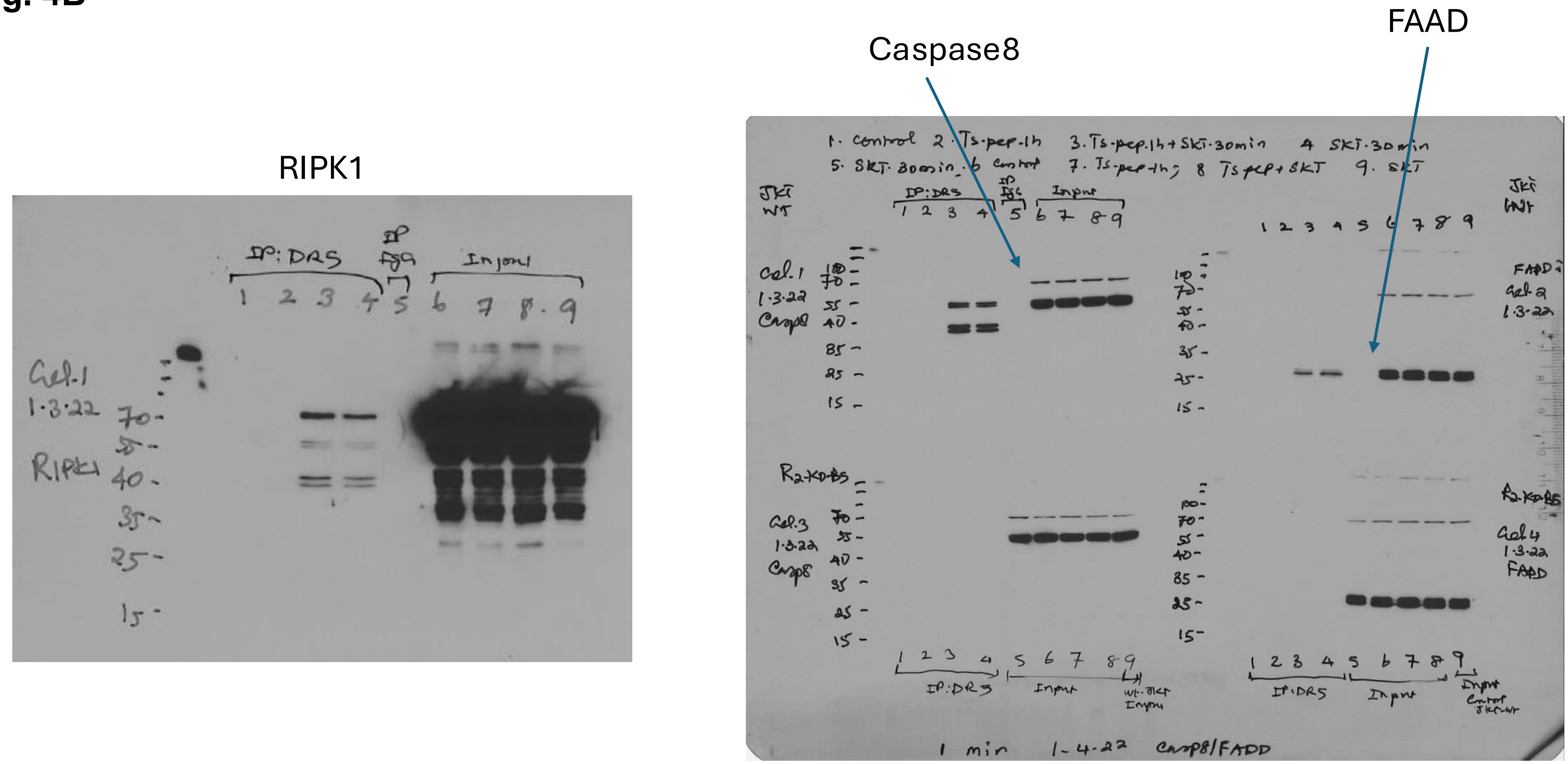

Jurkat cells

Fig. 5D

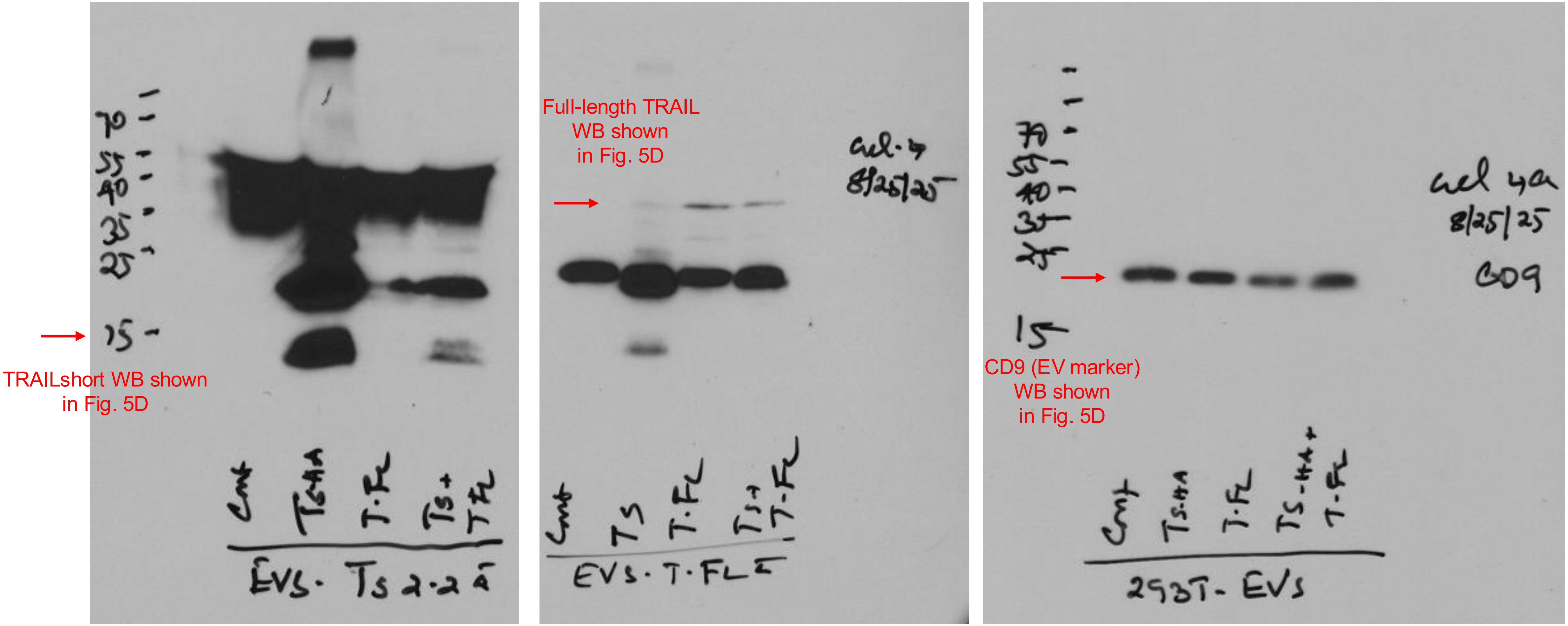
TRAILshort WB shown in Fig. 5D
Full-length TRAIL WB shown in Fig. 5D
CD9 (EV marker) WB shown in Fig. 5D

Fig. 5E

p-SHP-1 in Fig. 5E

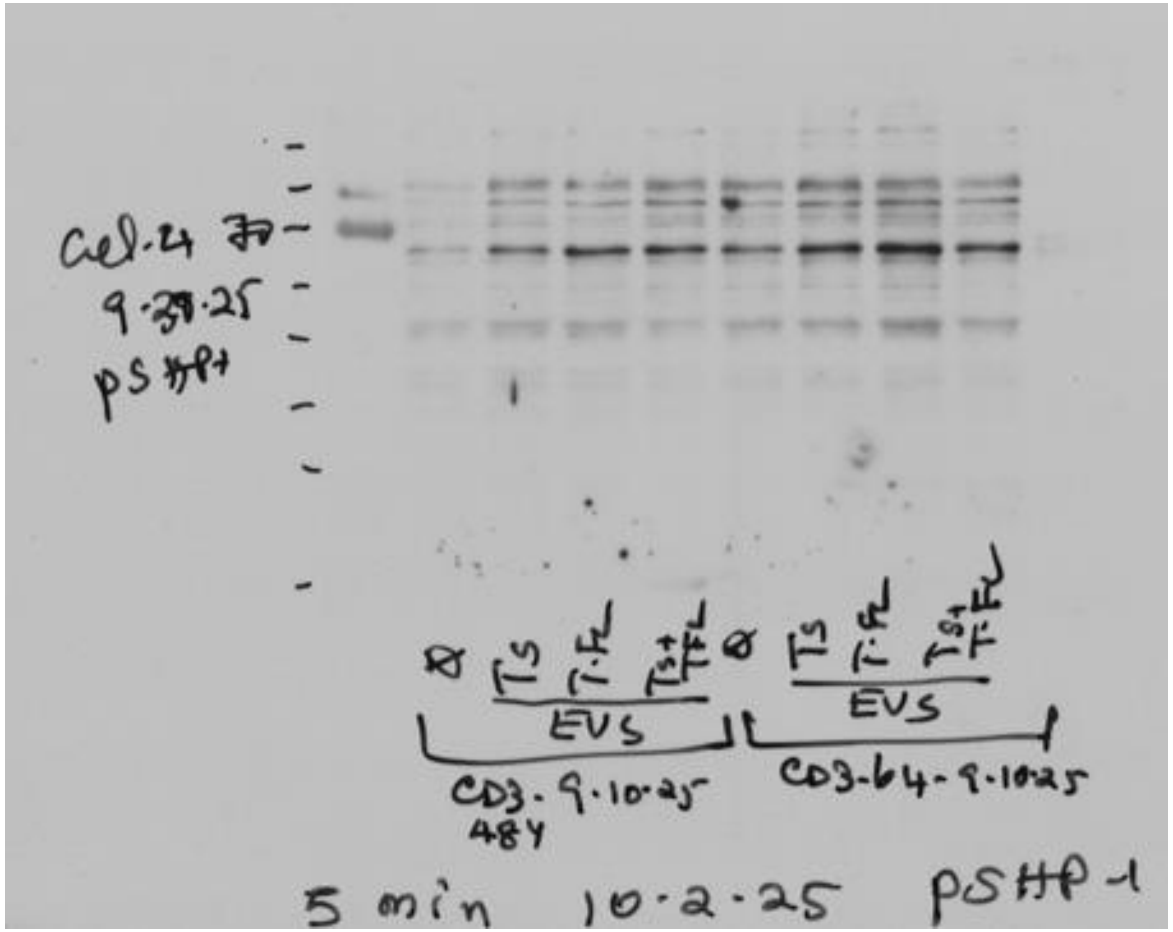

Total SHP-1 in Fig. 5E

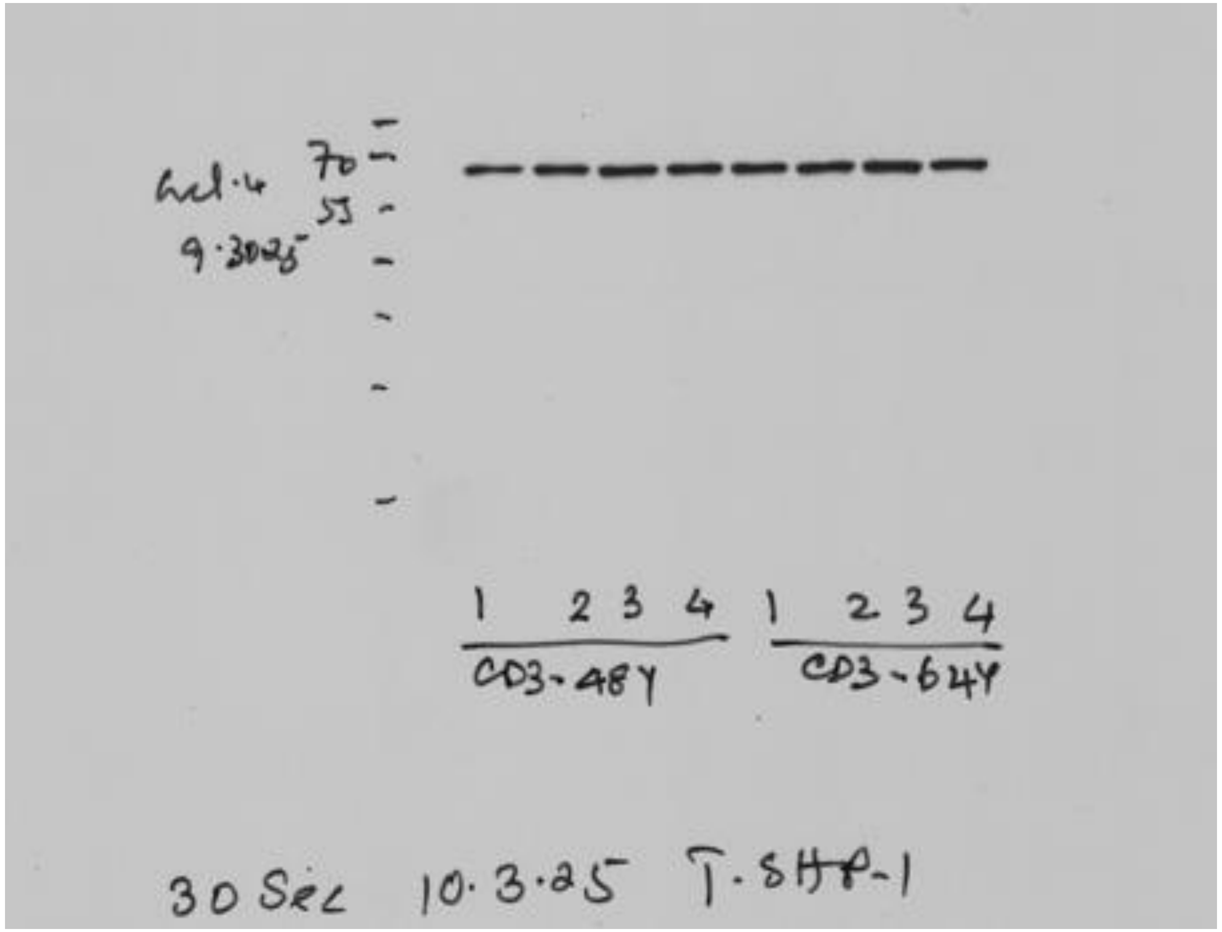

CD3 cells

Fig. 5G

Total SHP-1 | p-SHP-1 | DR5

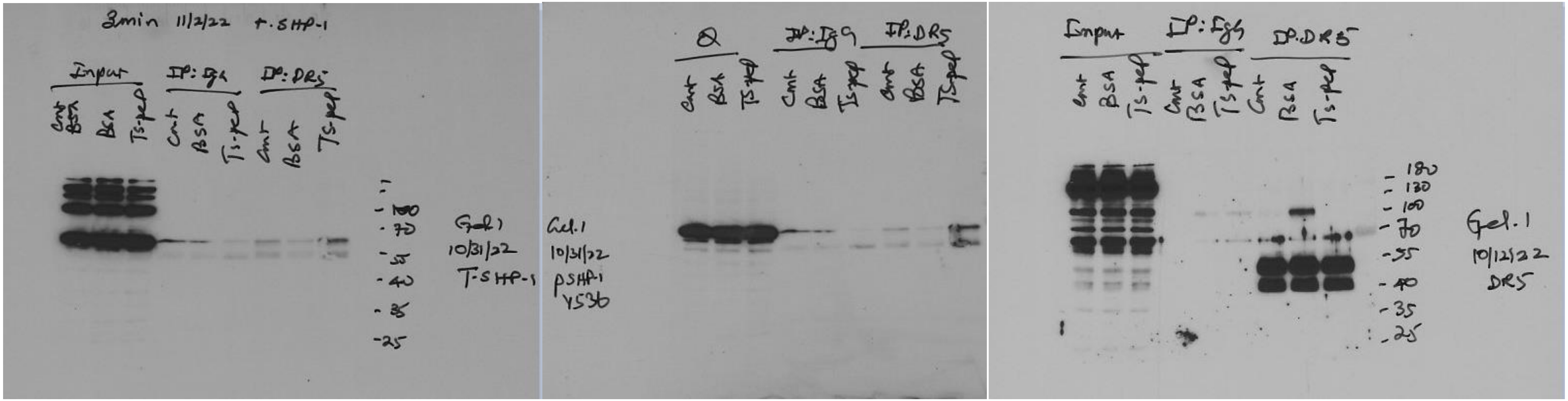

Jurkat cells

Fig. 5H

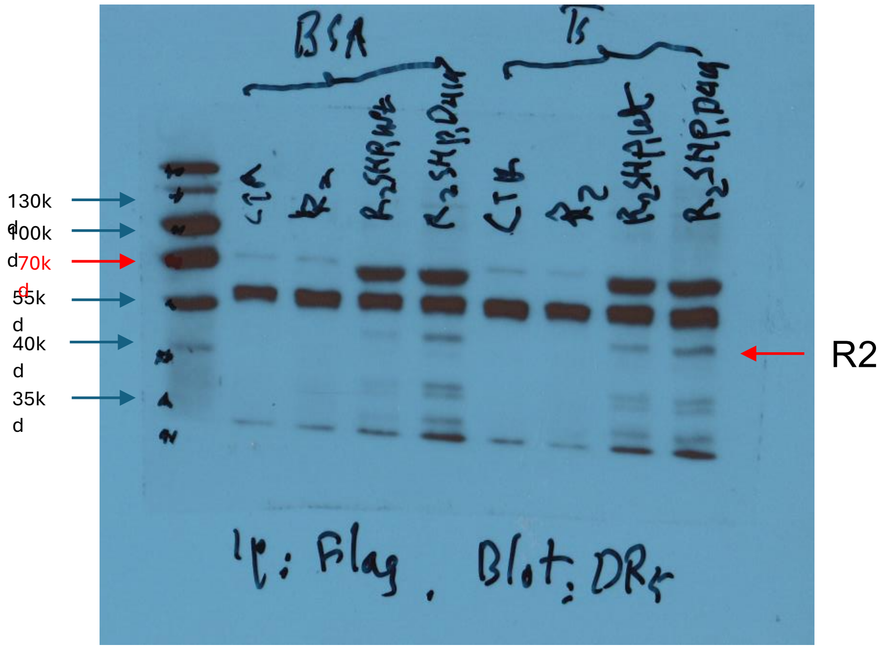

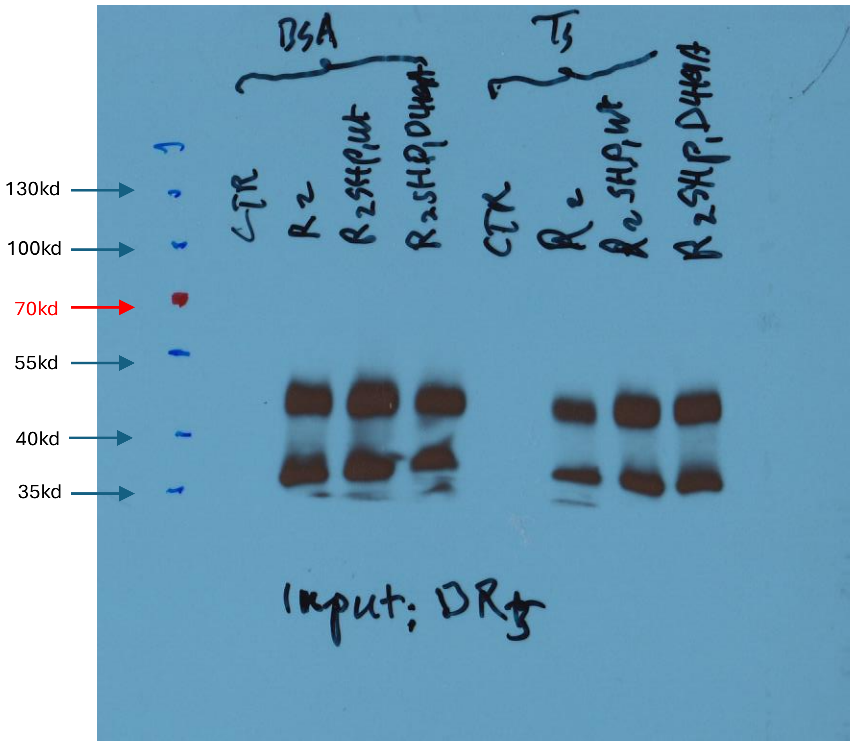

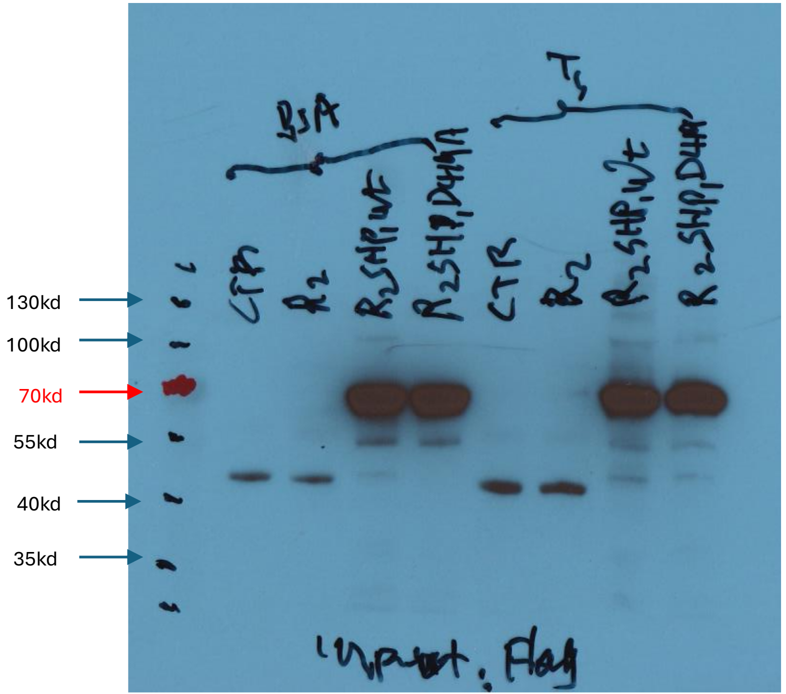

DR5 KO 293T cells cells

Fig. 6A and 6B

Total SHP-1
shown in Fig. 6A

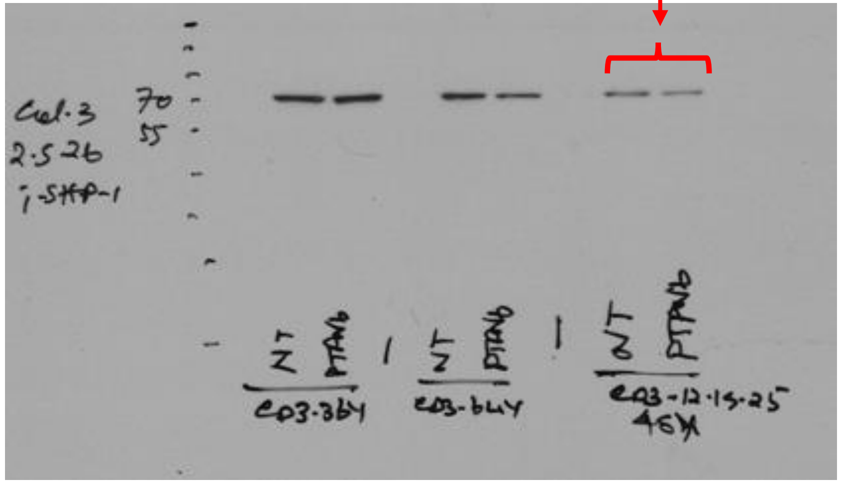

GAPDH
shown in Fig. 6A

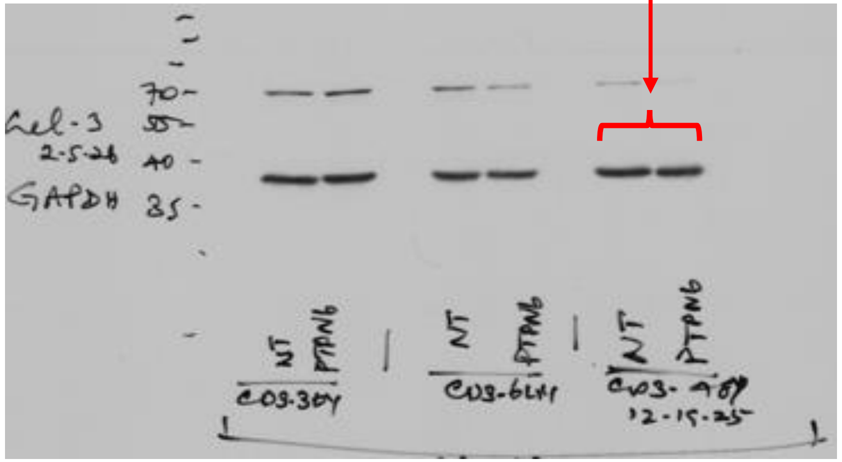

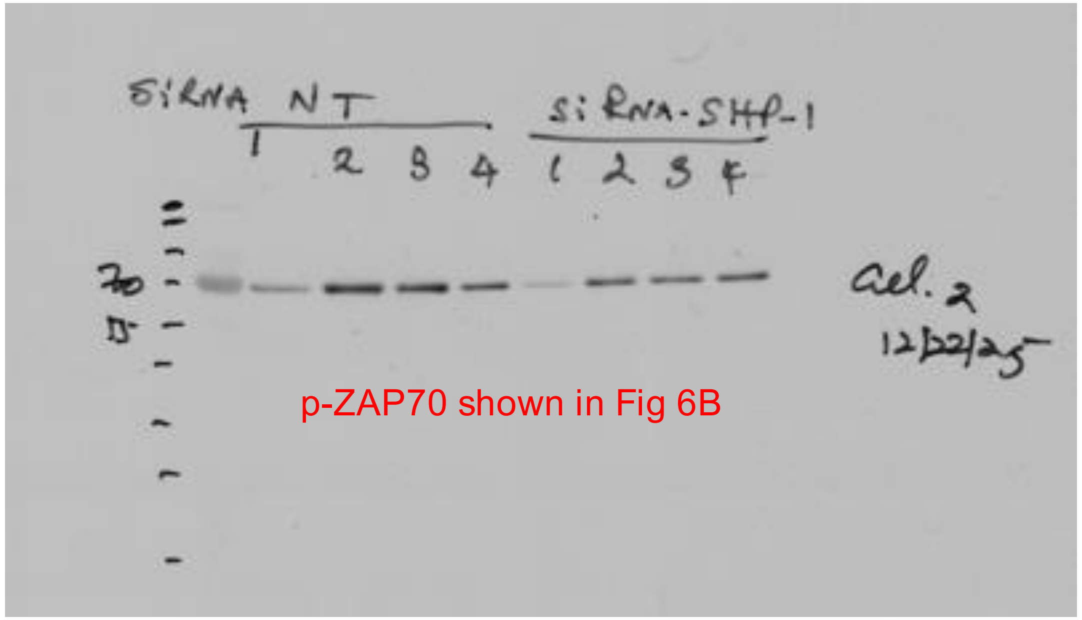

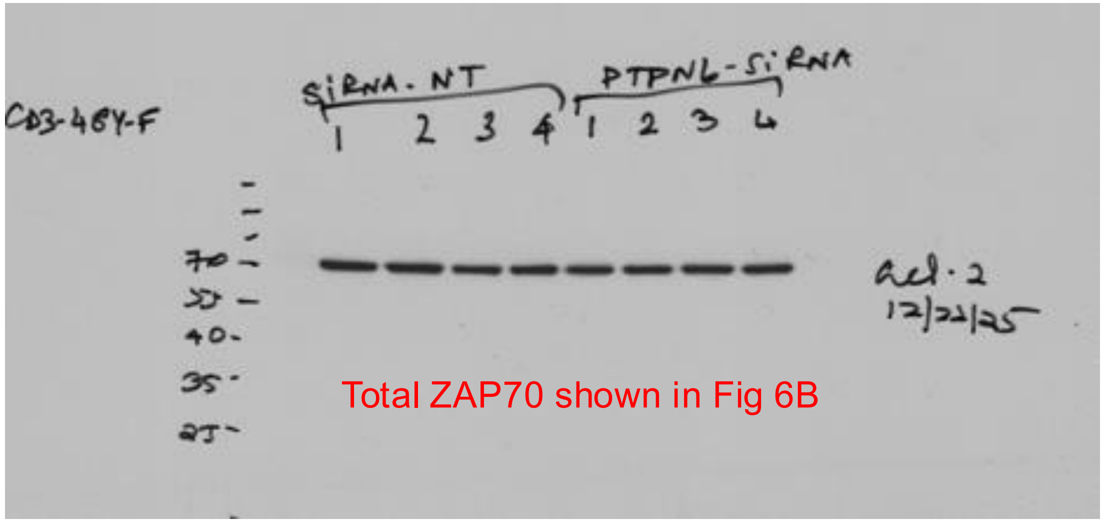

Fig. 6C and 6D

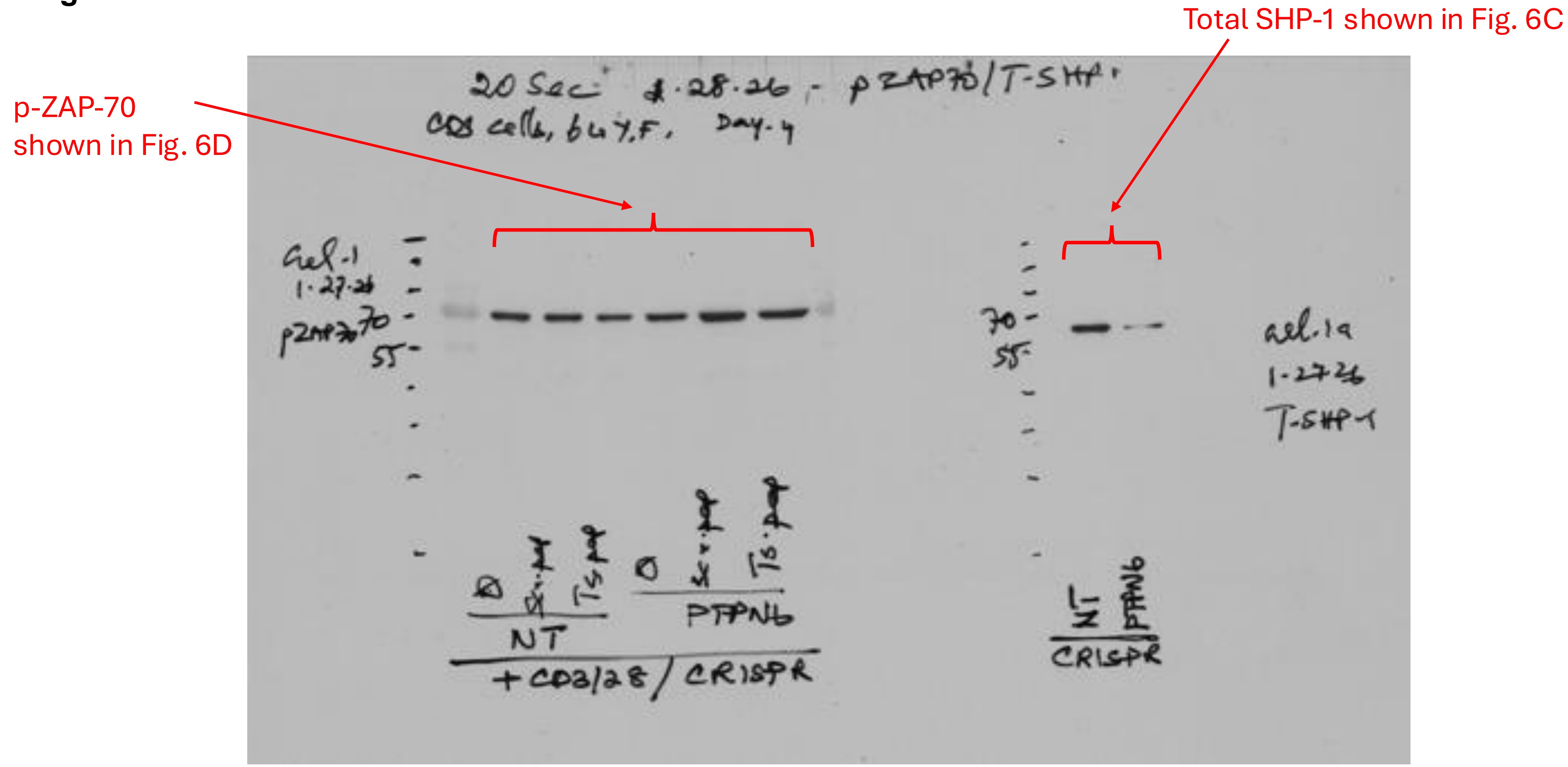

CD3 cells

Fig. 6C and 6D

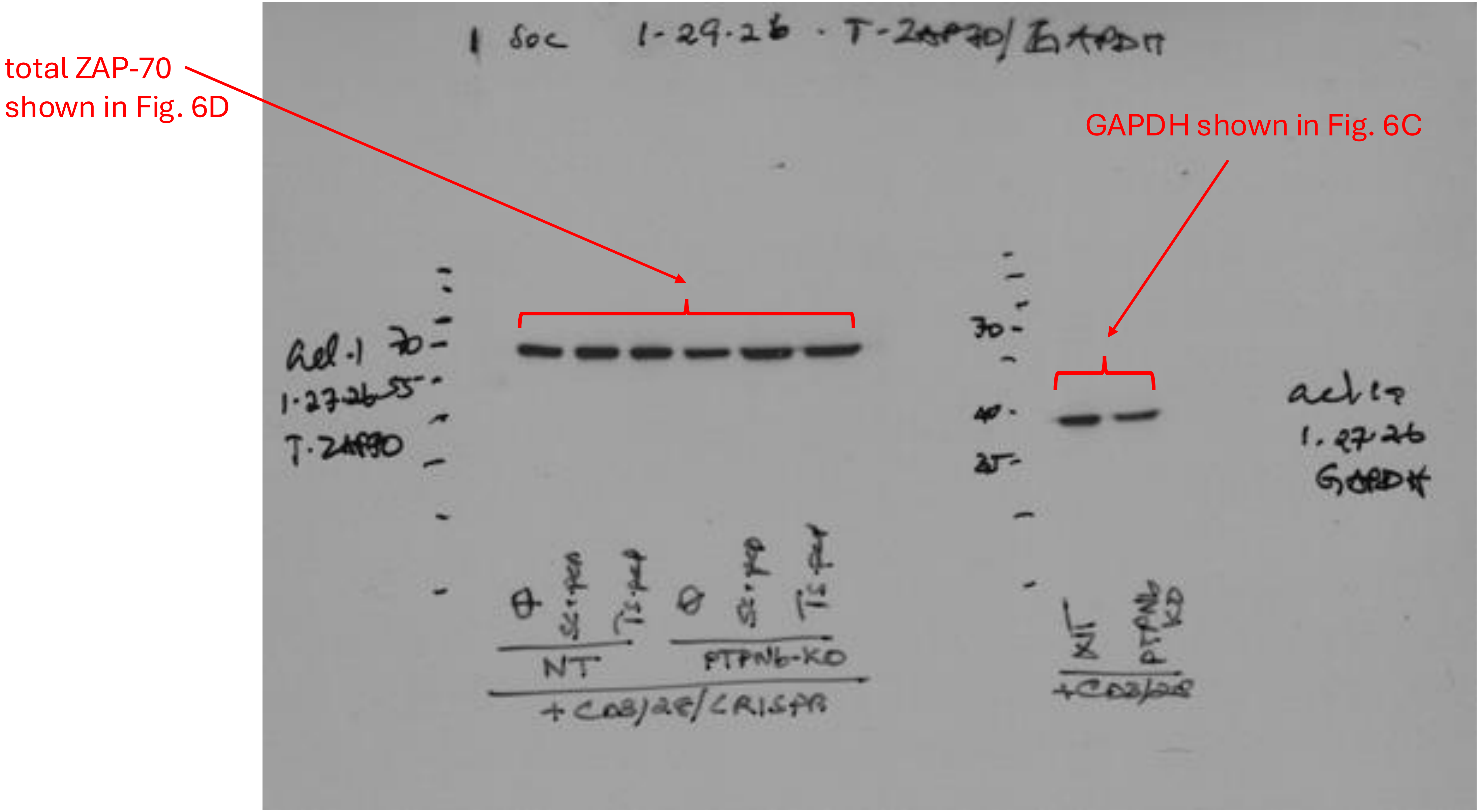

CD3 cells

Supplementary Fig. 1B

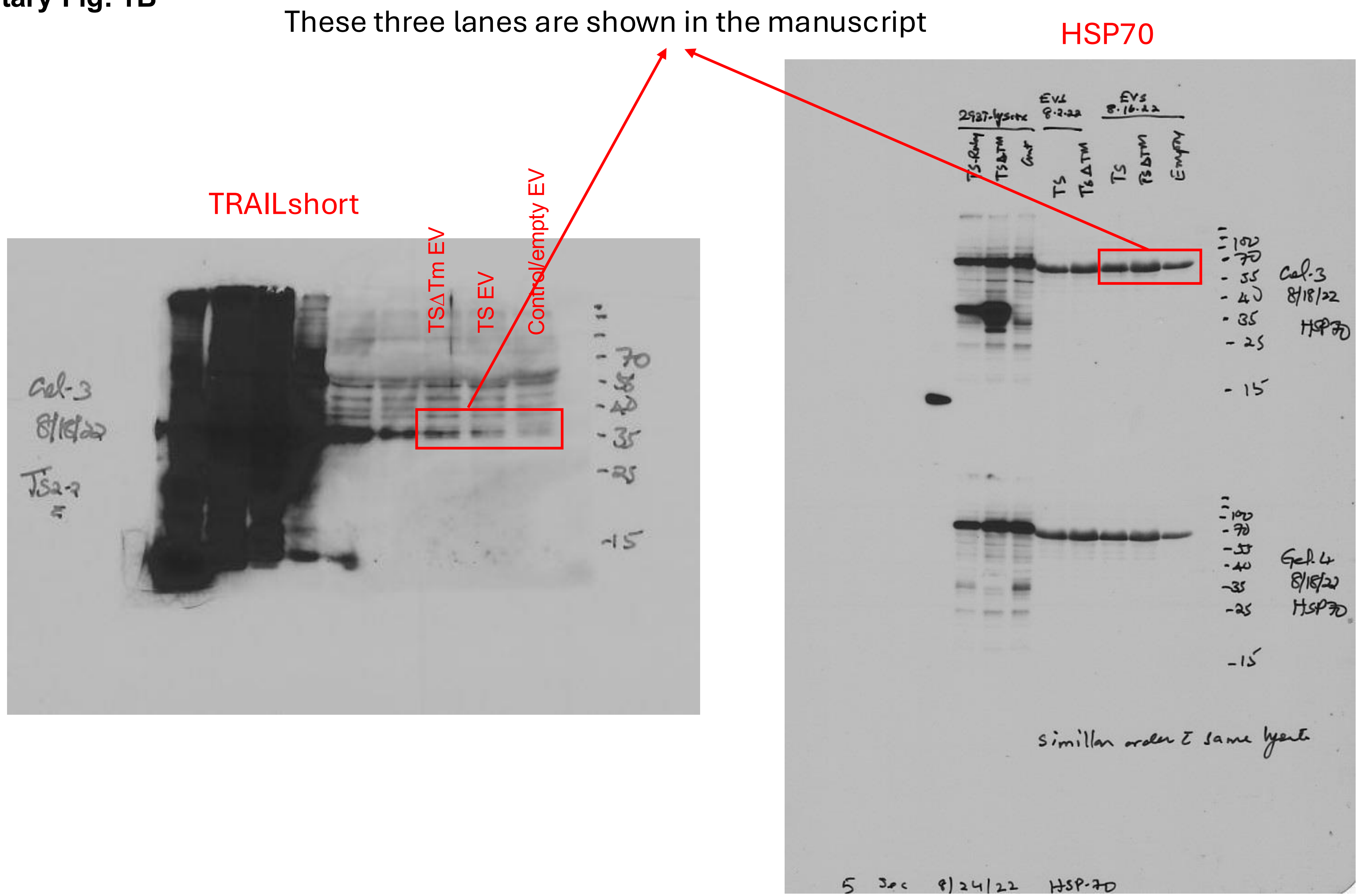

Supplementary Fig. 1C

The first two lanes are shown in the manuscript Fig.

GAPDH

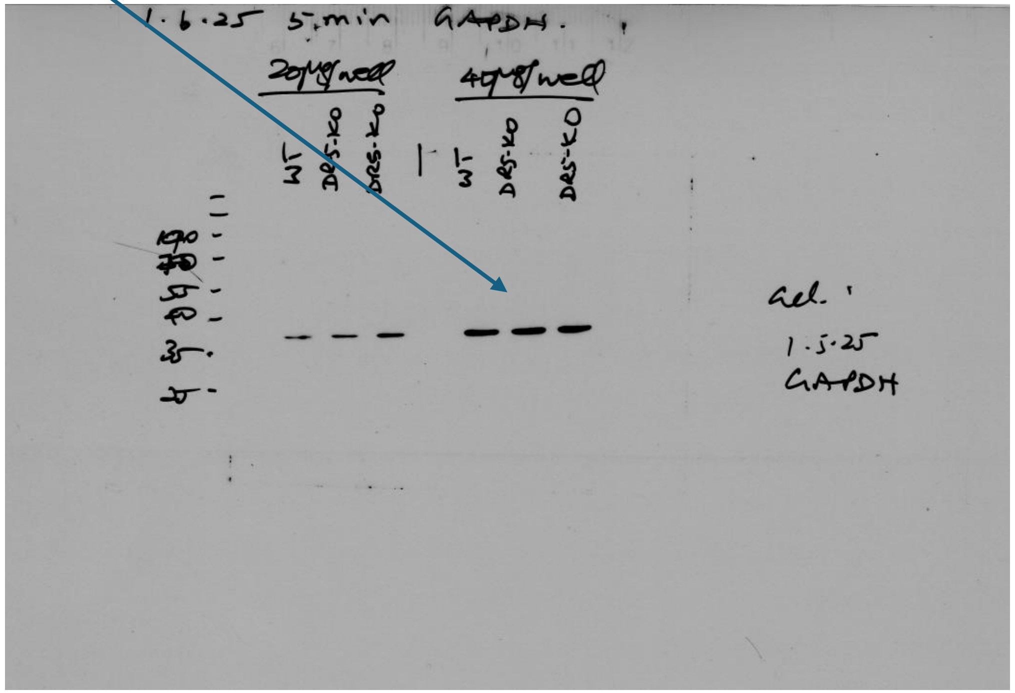

DR5

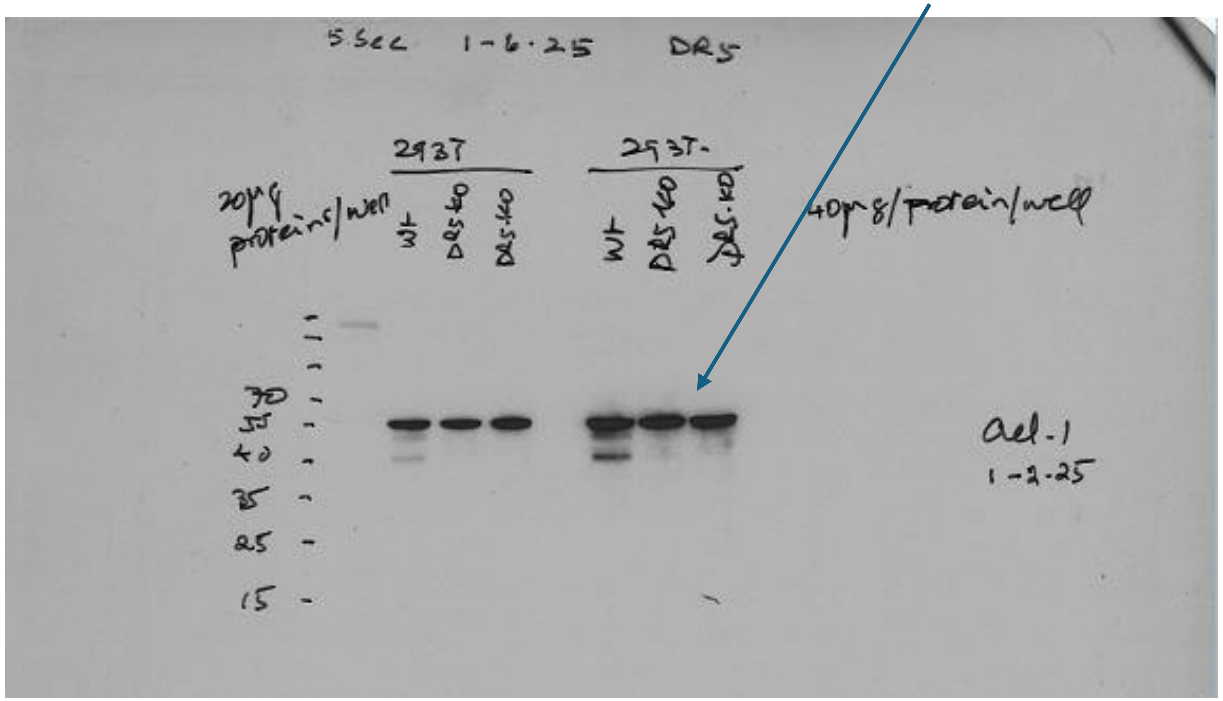

Supplementary Fig. 2C (phosphoprotein)

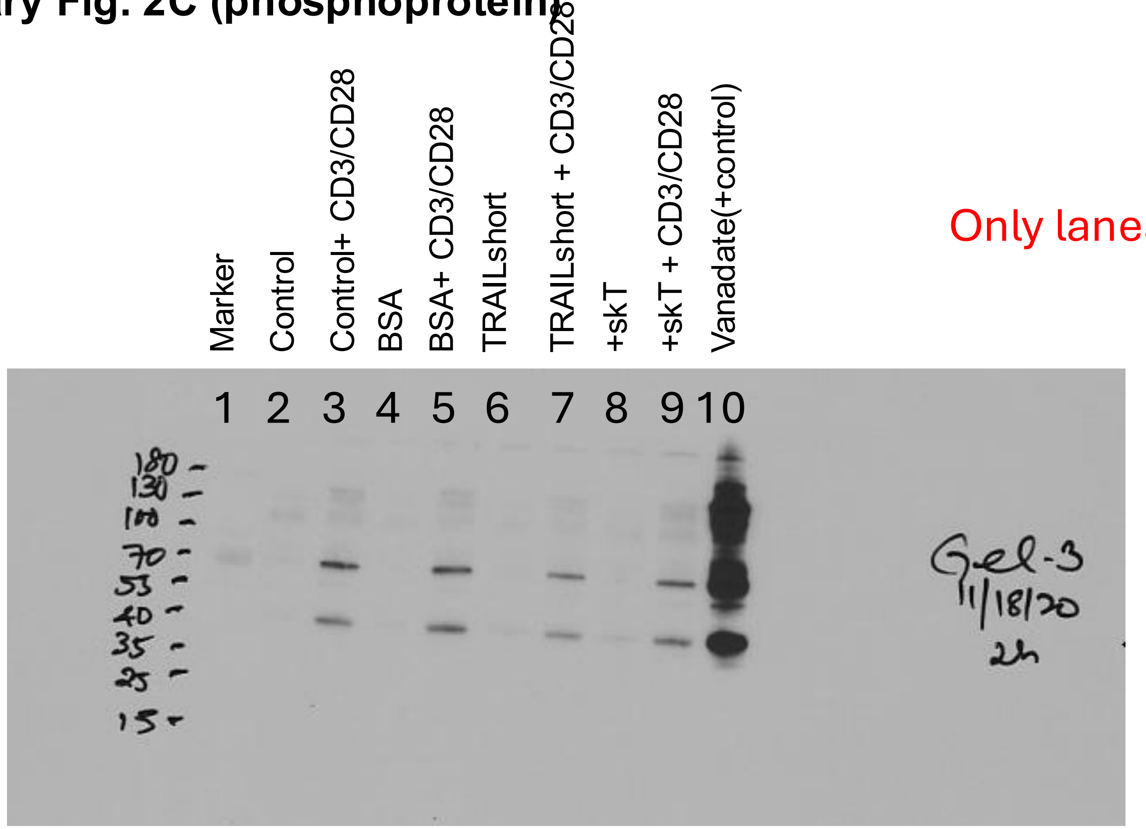

Only lanes #2 to #7 are shown in the Fig.2B of manuscript Figs

p-ZAP70 upper band (~70 kDa) and p-LAT lower band (~38kDa)

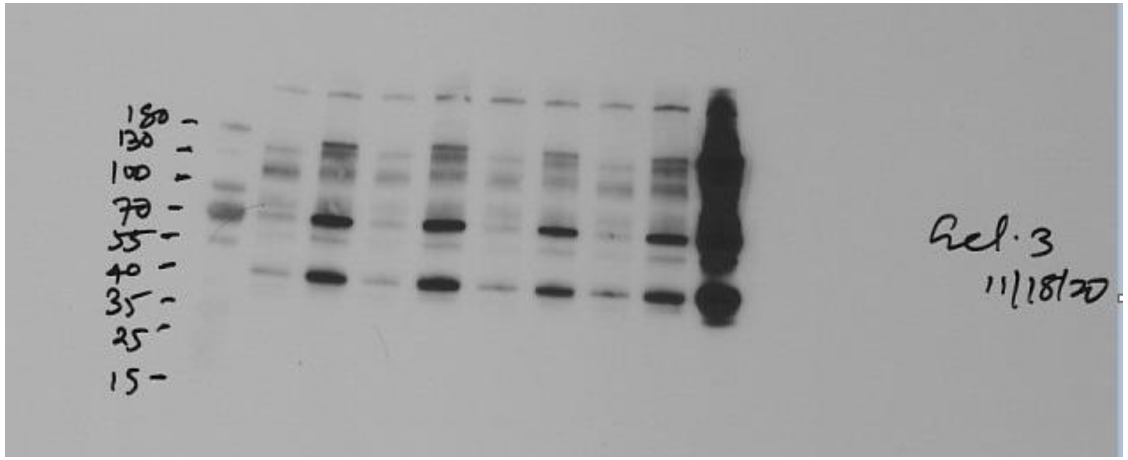

p-PLCgamma (upper band ~155kDa)

Supplementary Fig. 2C (total protein

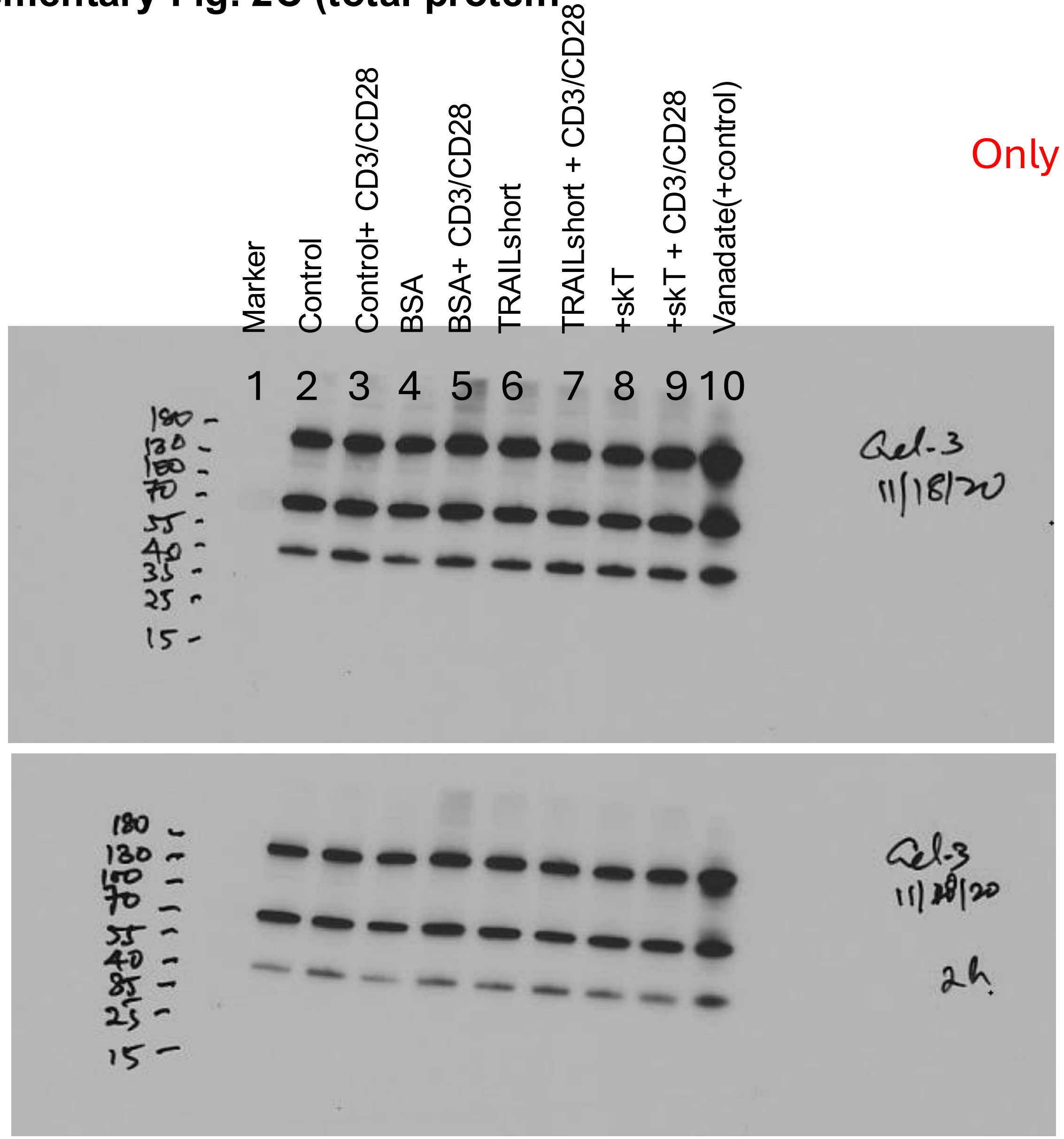

Only lanes #2 to #7 are shown in the Fig.2B of manuscript Figs

Used for total LAT (lower band)

Used for total ZAP-70 and total PLCgamma (upper and middle bands)

Supplementary Fig. 2D

WB for p-ZAP-70
in Fig. 2D

CD8 Effector memory T cells

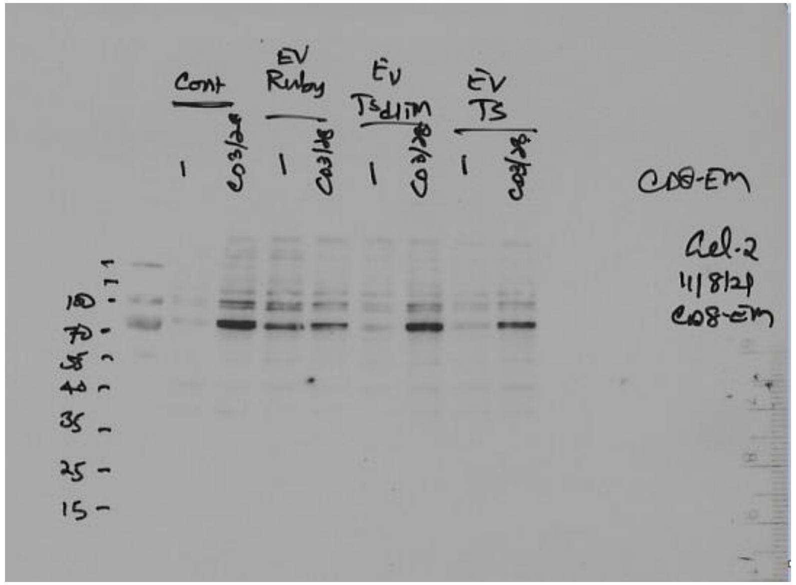

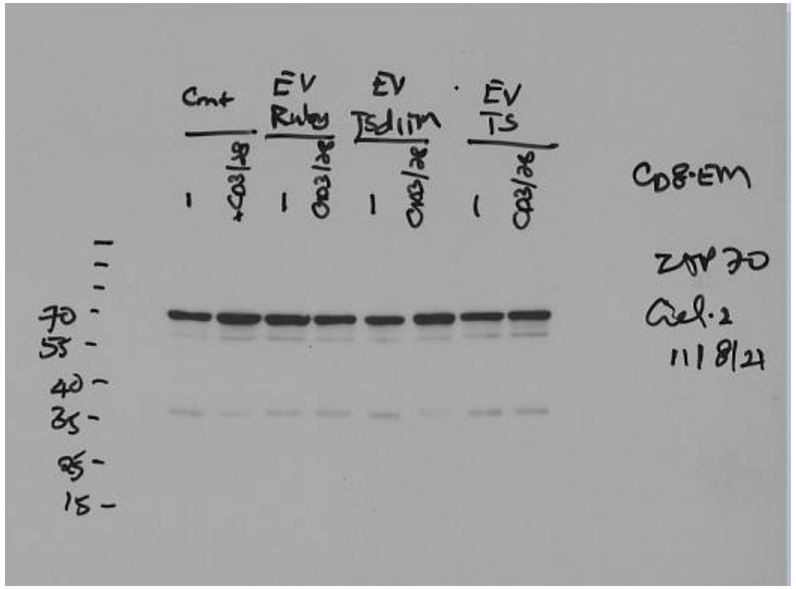

WB for ZAP-70
in Fig. 2D

WB for p-ZAP-70
In Fig. 2D

CD4 Effector memory T cells

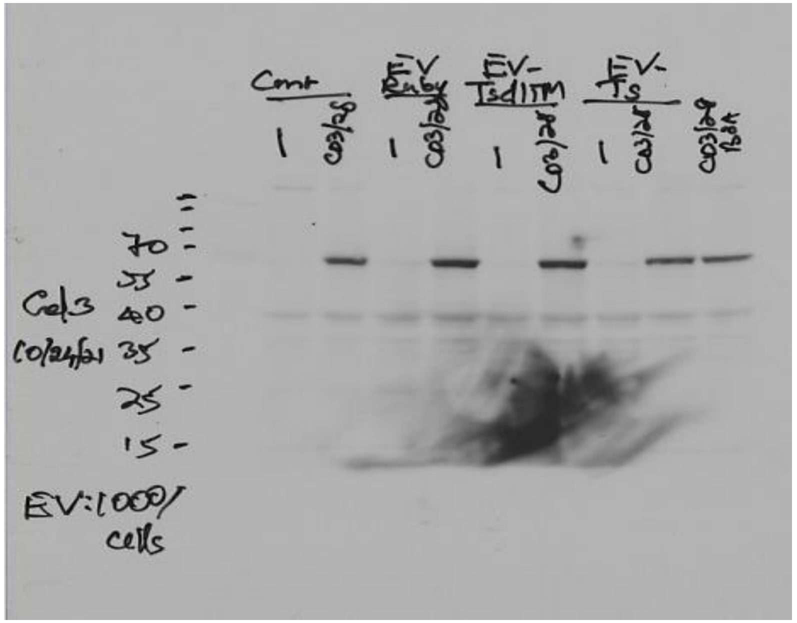

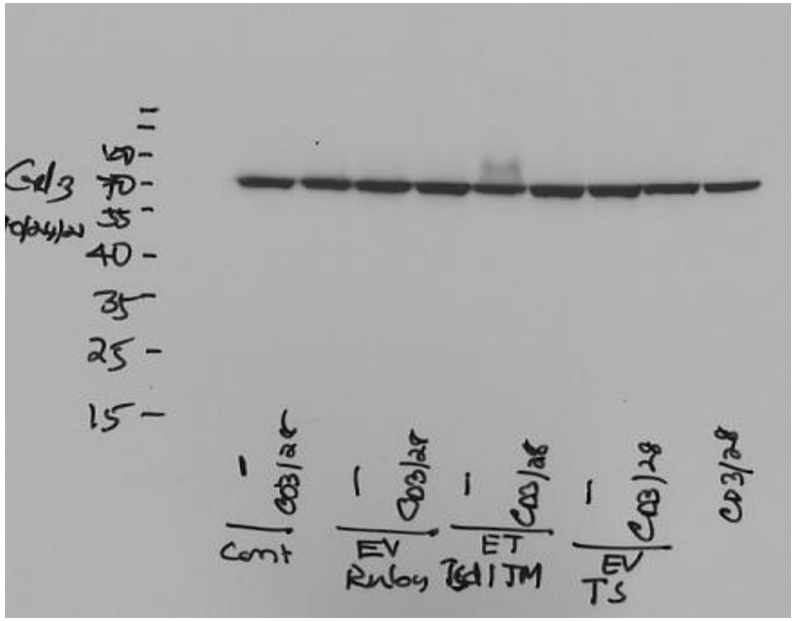

WB for ZAP-70
in Fig. 2D

Supplementary Fig. 4A

(Lanes 2-8 are cropped and shown in the manuscript).

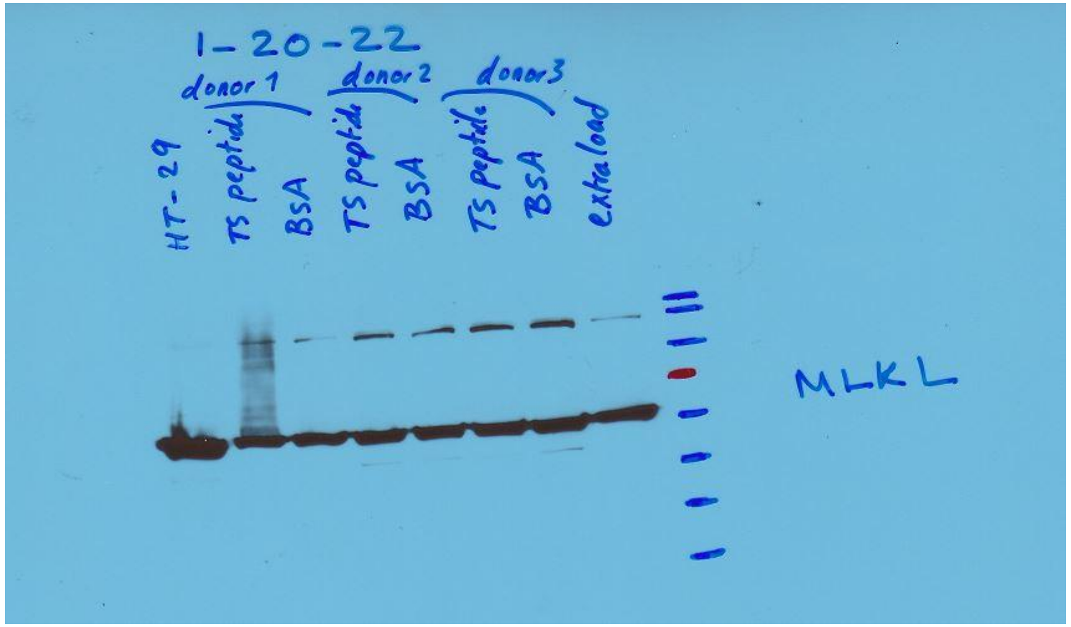

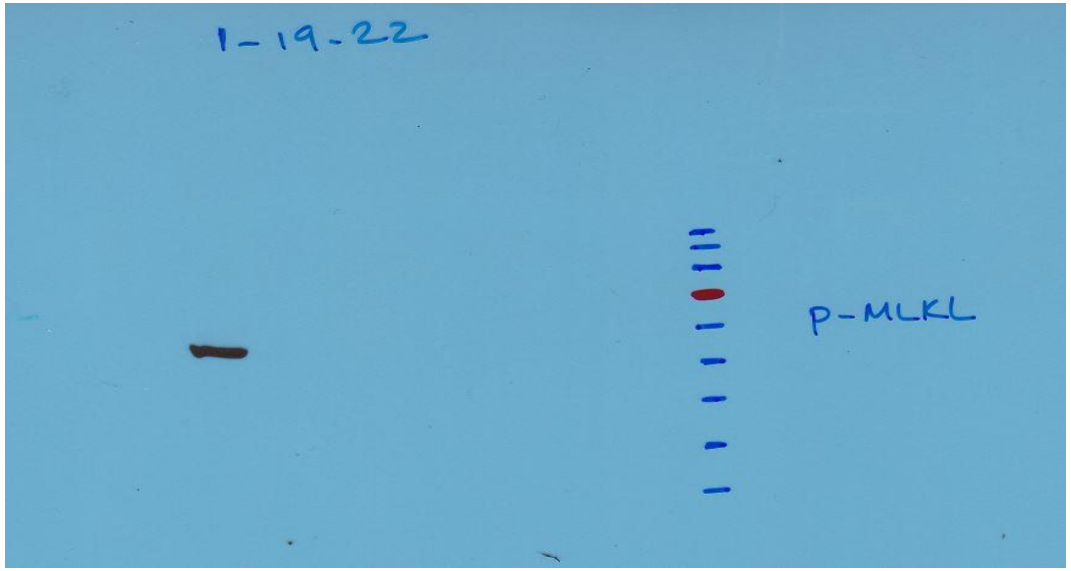

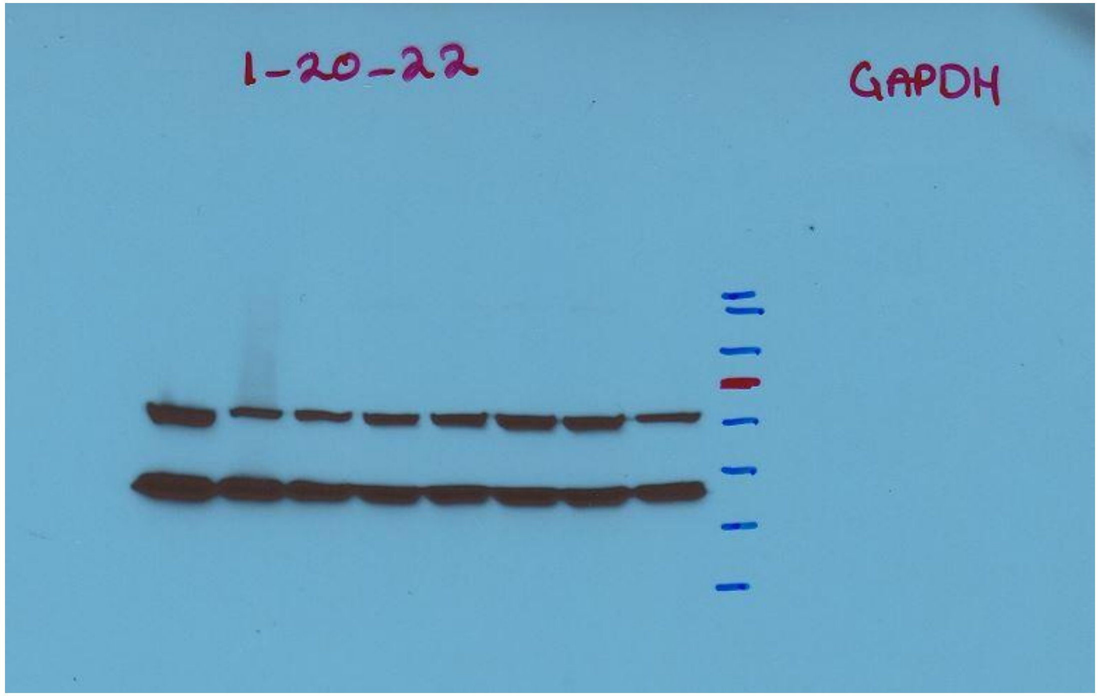

Supplementary Fig. 4B

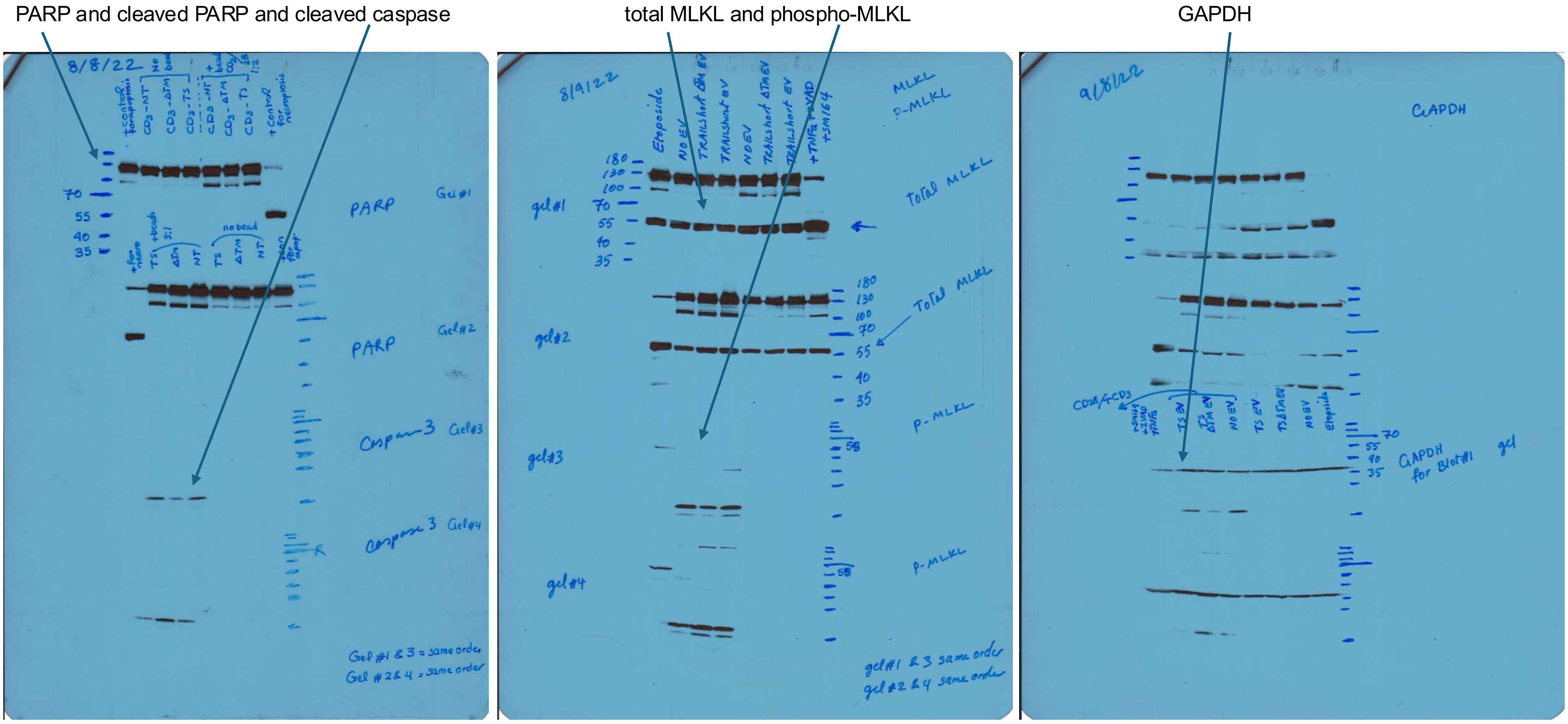

Supplementary Fig. 4C

p-SHP-1

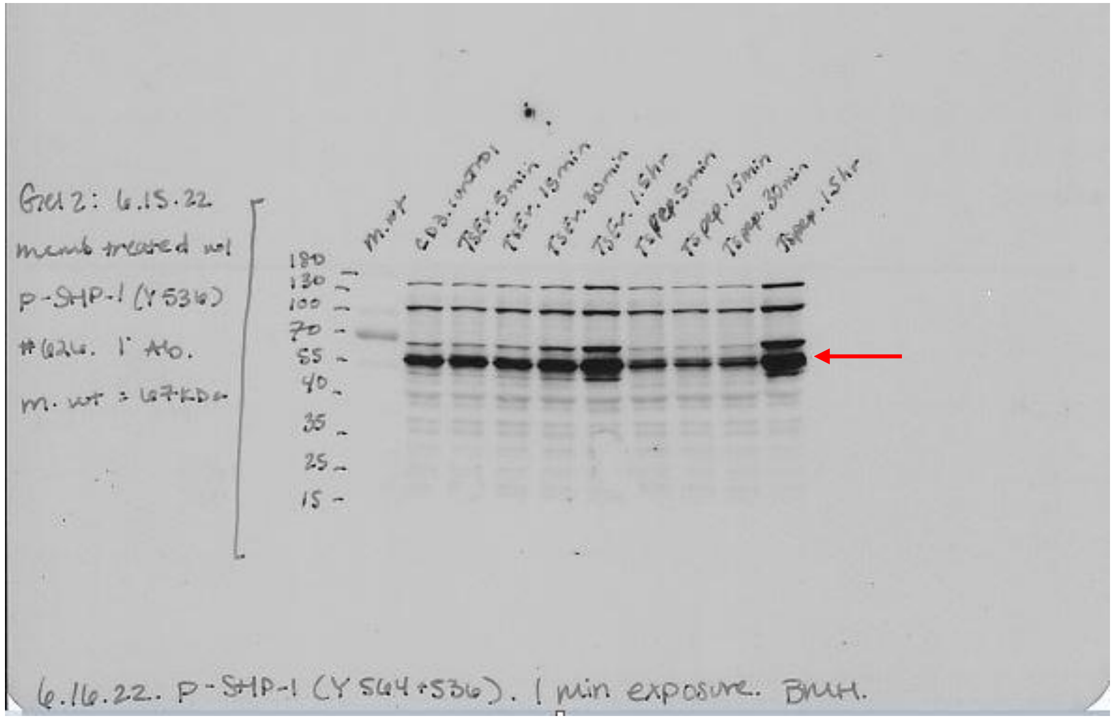

Total SHP-1

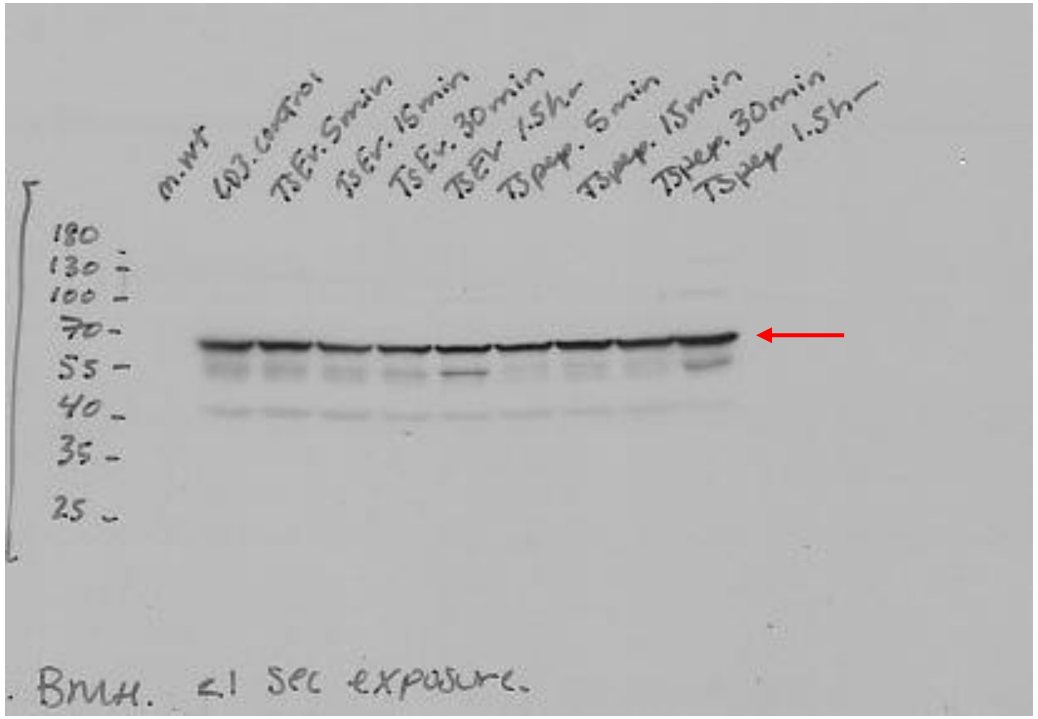

Supplementary Fig. 4D

p-SHP-1 for Suppl. Fig. 4D (left panel)

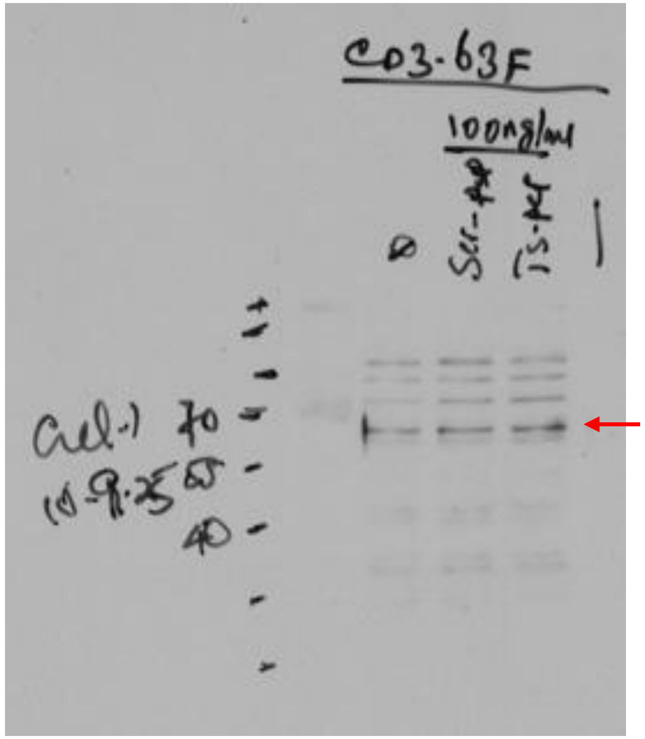

Total SHP-1 for Suppl. Fig. 4D (left panel)

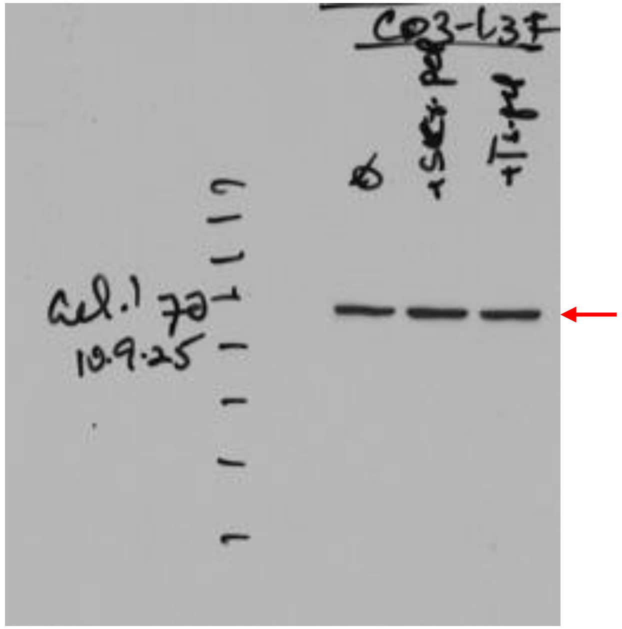

p-SHP-1 for Suppl. Fig. 4D (right panel)

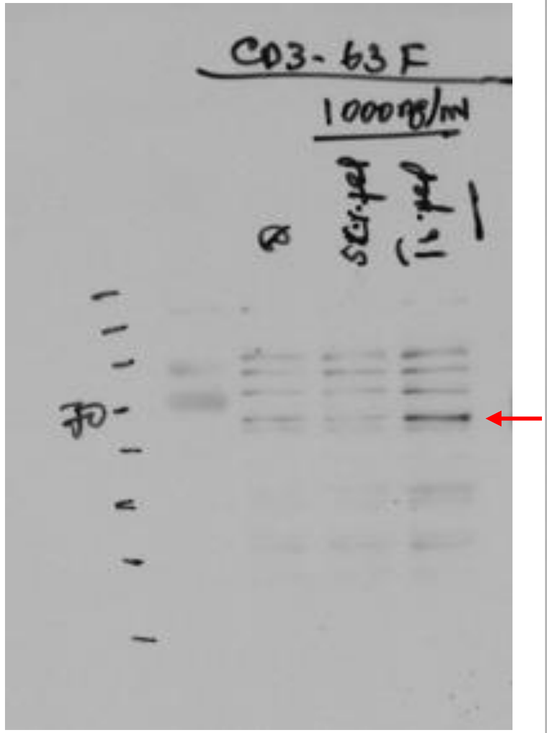

Total SHP-1 for Suppl. Fig. 4D (right panel)

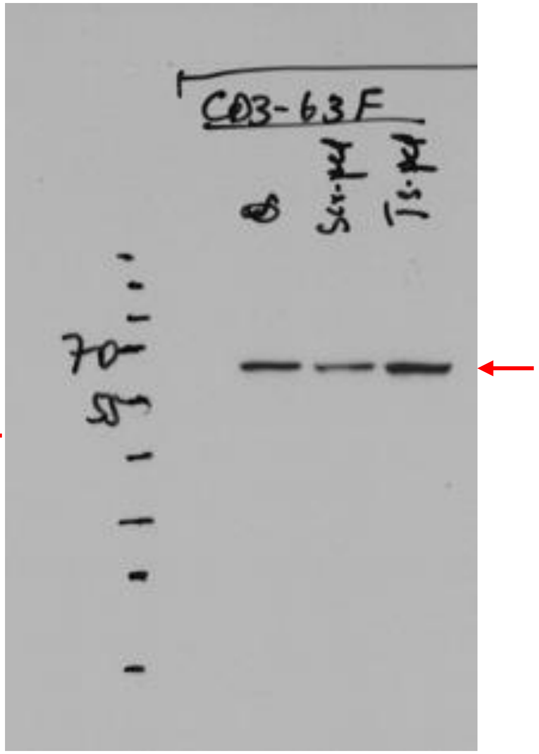

Supplementary Fig. 4E

Phospho-ZAP-70 for Suppl Fig. 4E

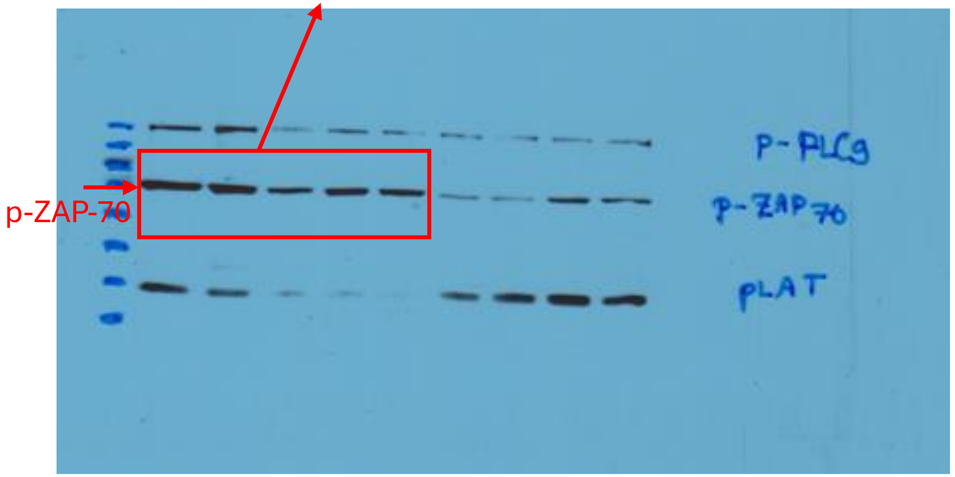

Total ZAP-70 shown for Suppl Fig. 4E

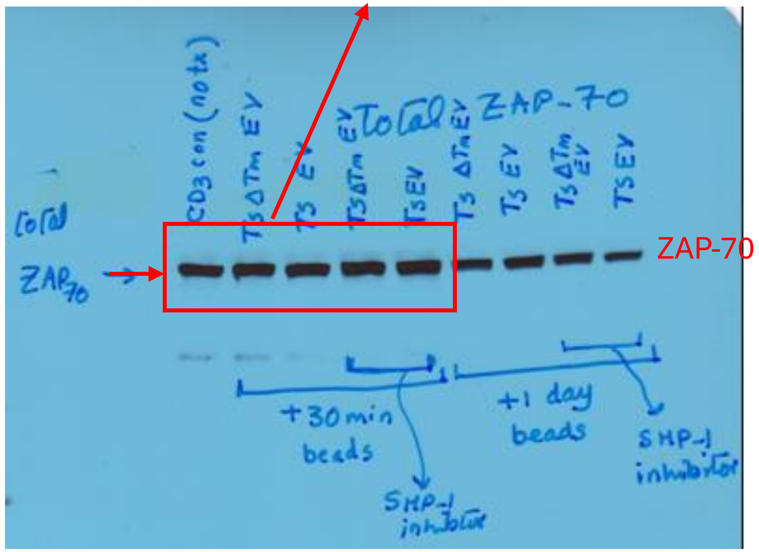

GAPDH shown for Suppl Fig. 4E

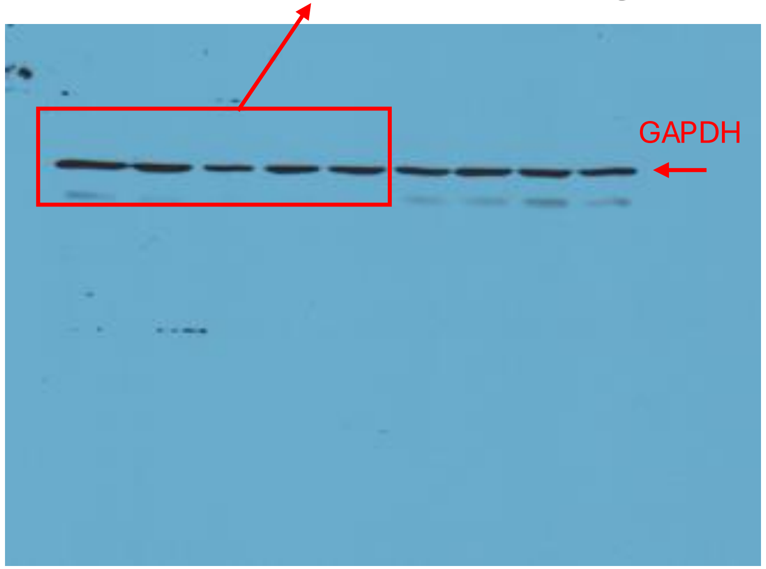

Supplementary Fig. 4F

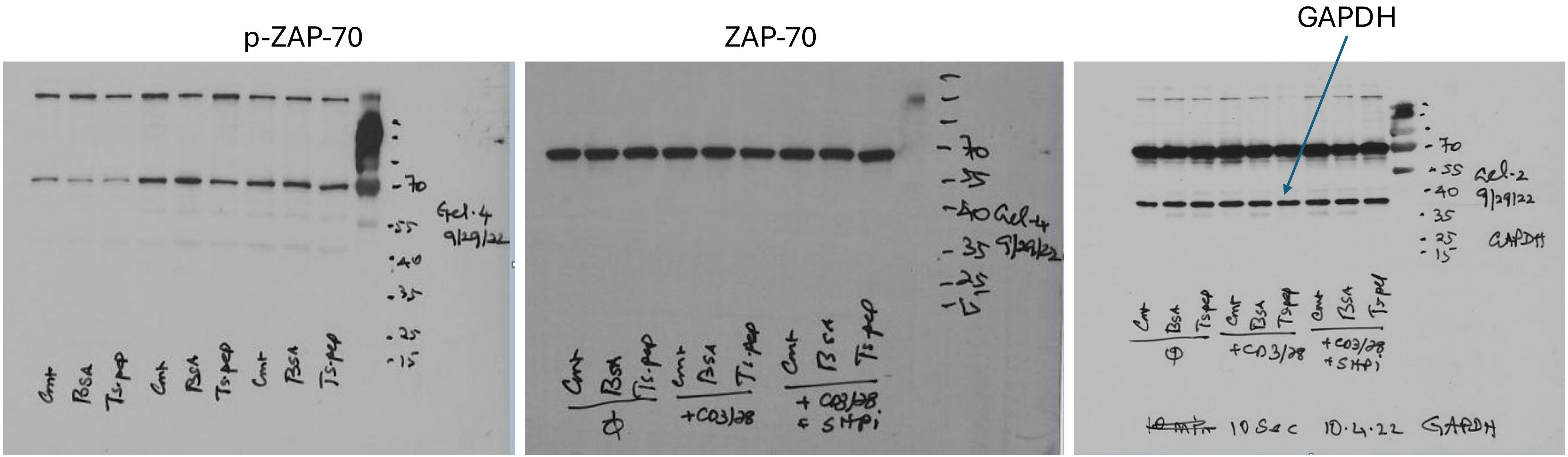

Supplementary Fig. 5B (First 4 lanes were cropped for the paper)

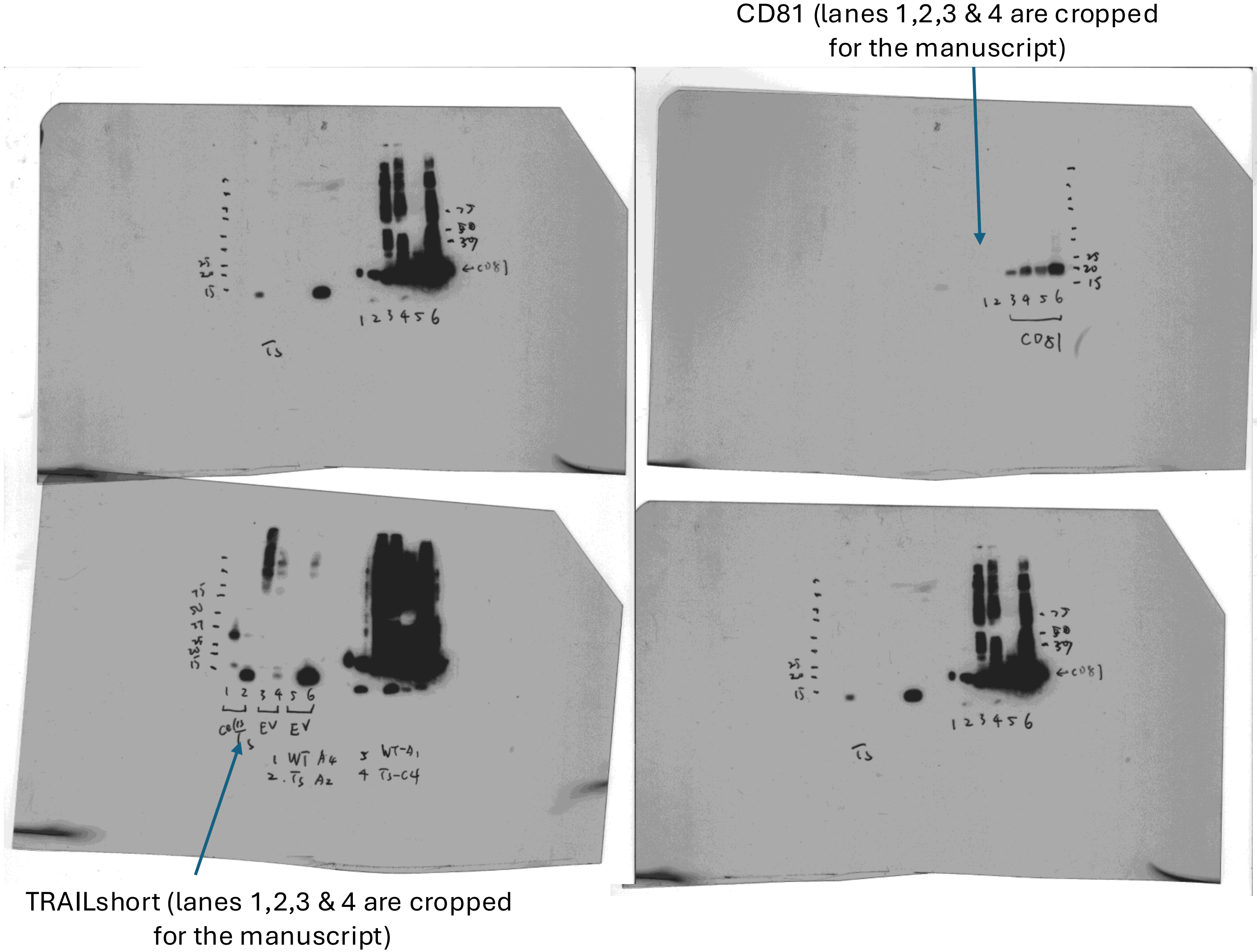

Supplementary Fig. 6B
(First 2 lanes were cropped for the paper)

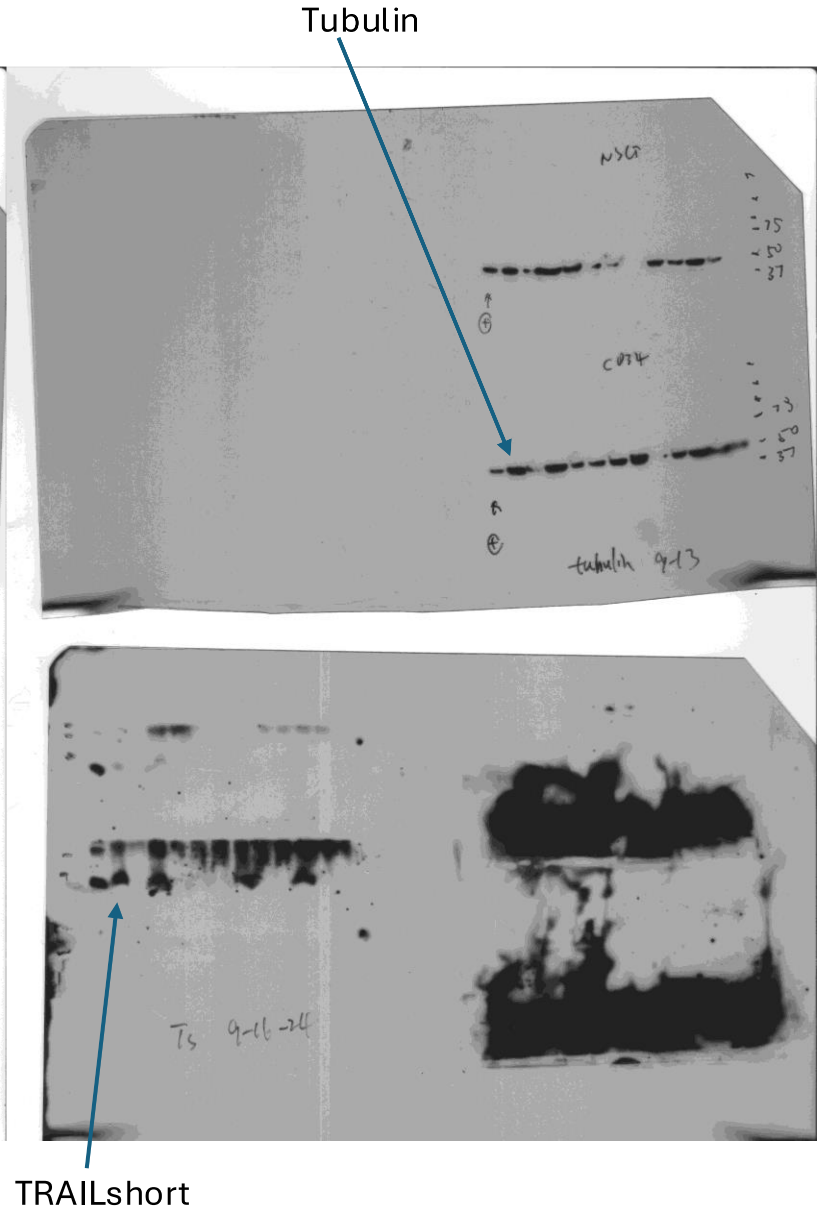

Supplementary Fig. 7

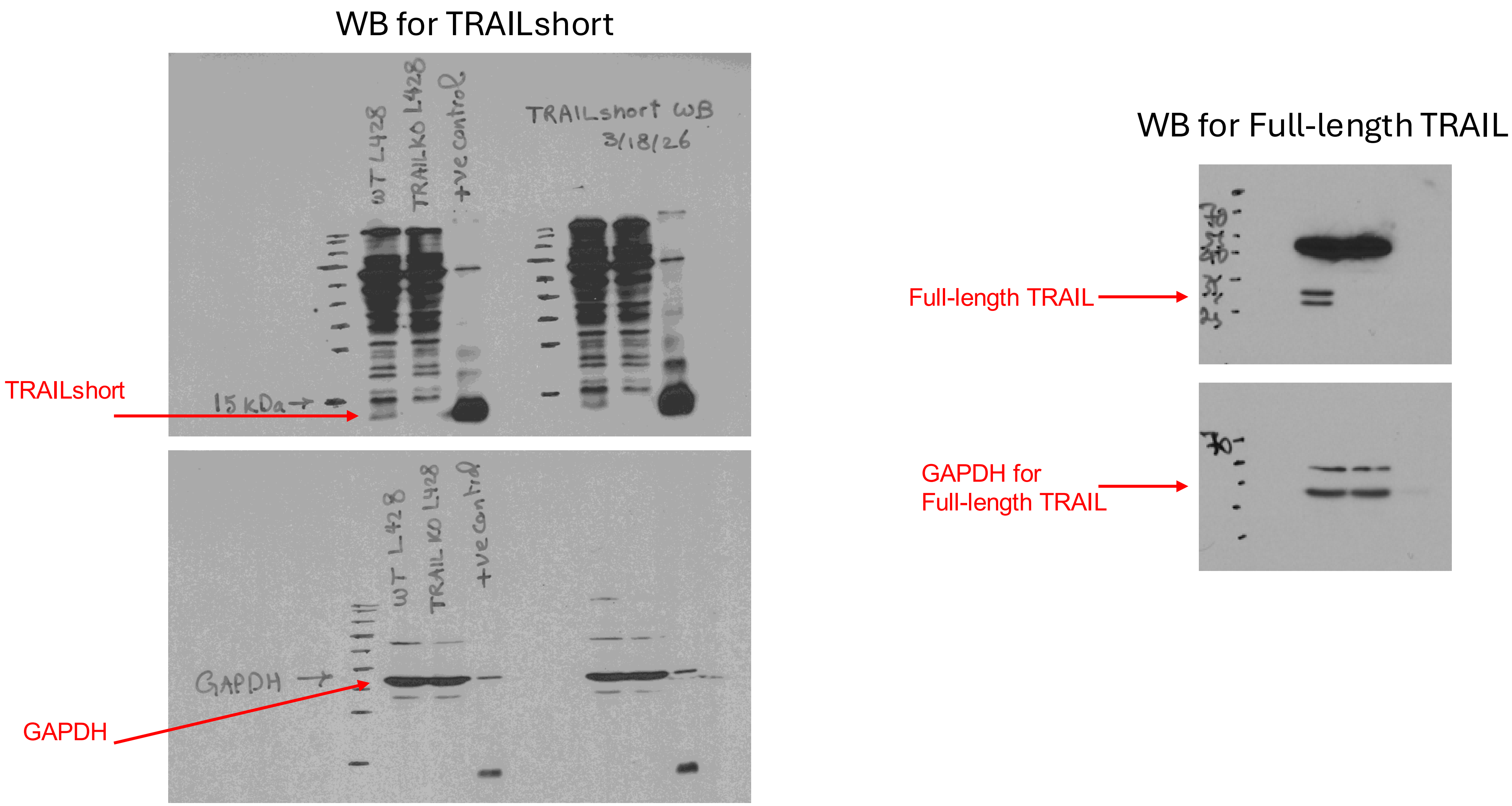

Supplement: Unedited blot and gel images [file jci-136-194449-s052.pdf]
